# The Role of Confinement in Biomineralization

**DOI:** 10.1021/acs.chemrev.5c00659

**Published:** 2025-11-18

**Authors:** Yifei Xu, Johanna M. Galloway, L. Jorin Hasselt, Fiona C. Meldrum

**Affiliations:** † School of Chemistry, 4468 University of Leeds, Leeds LS2 9JT, U.K.; ‡ State Key Laboratory of Molecular Engineering of Polymers, Department of Macromolecular Science, 12478 Fudan University, Shanghai 200438, China

## Abstract

This review focuses on an important but under-explored biogenic strategy used to control biomineralization processes—confinement—where compartmentalization is fundamental to the organization and function of all organisms. Biominerals combine the functionality of inorganic and organic solid-state materials and are constructed under precise biological control. Often exhibiting desirable properties, such as high strength, toughness, and complex morphologies that surpass those of synthetic materials synthesized under harsher conditions, biomineral formation processes are widely studied. Here we demonstrate the vital role that confinement plays in defining the key structural characteristics of biominerals and in controlling their mechanisms of formation. These range from well-accepted functions, such as stabilizing amorphous phases, isolating the mineralization site, and controlling morphologies, to more speculative roles, including controlling crystal nucleation, orientation and polymorphism. Examples from a range of organisms, mineral types, and length scales are provided, and further insight into potential biogenic mechanisms is gained through comparison with crystallization in complementary confined synthetic systems. Further opportunities for exploring confinement effects in biomineralization systems are discussed throughout, where these will ultimately act as an inspiration for the synthesis of sustainable materials, for medical innovations, as well as providing insights into evolution and environmental change.

## Graphical abstract

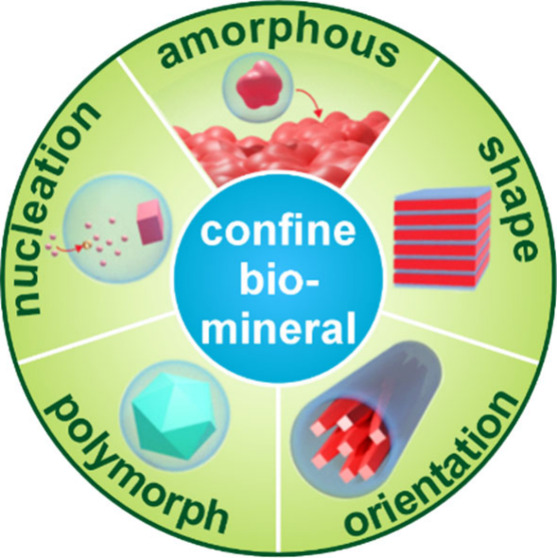

## 1 Introduction

Biominerals are found throughout the three domains of life in Archaea, Bacteria, and Eukarya, and at least 60 different minerals have been identified from the biosphere.^1,2^ These organic–inorganic hybrid materials are natural engineering marvels that have evolved to fulfill a wide range of functions, including predation, mastication, locomotion, light-guiding, gravity sensing, defense, camouflage, magneto-reception, and nutrient storage (Figure 1).^3–11^ Biominerals also often exhibit morphologies and properties that are entirely distinct from their synthetic counterparts.^12^ While synthetic crystals exhibit morphologies that reflect the symmetry of the underlying lattice, biominerals frequently adopt complex nongeometric forms. Similarly, nature tunes the toughness and hardness of biominerals by occluding small quantities of organic molecules within single crystals.^13–15^ The design strategies that underlie the construction of these specialized materials, and the mechanisms by which they form, have been widely studied,^1,2,12,15–21^ as they offer a unique inspiration for the development of bioinspired mineralization approaches.^18,22–26^

**Figure 1:**
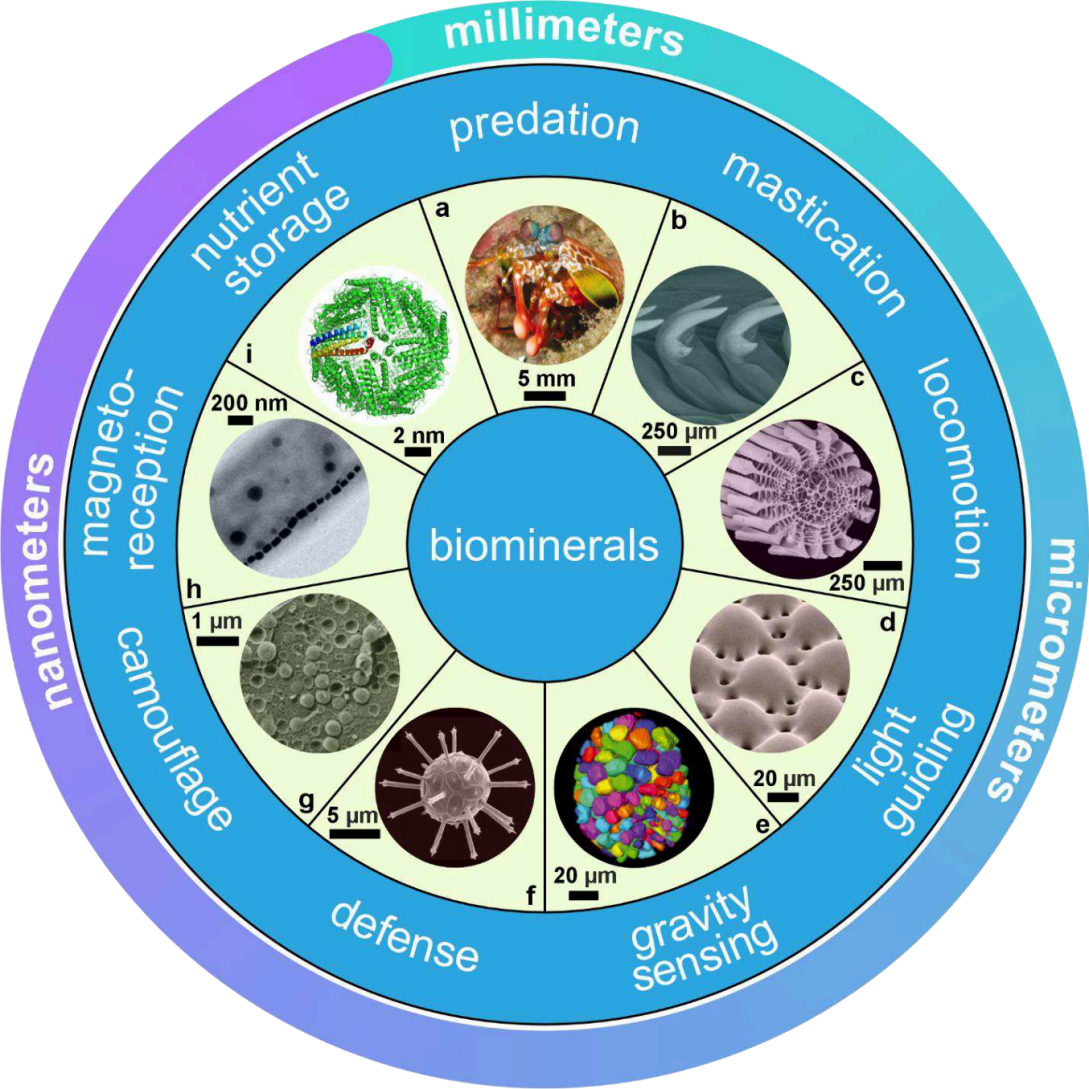
**Examples of sophisticated biomineral structures that perform a range of biological functions across multiple length scales.** (a) Predation: dactyl club of stomatopod *Odontodactylus scyllarus*. Reproduced with permission from Weaver et al.^5^ Copyright 2012 The American Association for the Advancement of Science. (b) Ingestion: radular teeth of chiton *Cryptochiton stelleri*. Reproduced with permission from Weaver et al.^4^ Copyright 2010 Elsevier Ltd. (c) Locomotion: fractured tip of a sea urchin spine from *Astropyga magnifica*. Reproduced with permission from Donovan.^27^ Copyright 2018 Akademie der Naturwissenschaften Schweiz (SCNAT). (d) Light-guiding: brittlestar lenses from *Ophiocoma pumila*. Reproduced from Aizenberg et al.^7^ Copyright 2001 Macmillan Magazines Ltd. (e) Gravity sensing: statocyst from jellyfish medusa *Sanderia malayensis*. Reproduced with permission from Heins et al.^10^ Copyright 2018 The Author(s) & Inter-Research with a CC-BY license. (f) Defense: coccolith *Rhabdosphaera clavigera*. Adapted with permission from Monteiro et al.^3^ Copyright 2016 The American Association for the Advancement of Science under a CC-BY license. (g) Camouflage: eyeshine reflector from *Macrobrachium rosenbergii* larval eyes. Reproduced with permission from Shavit et al.^8^ Copyright 2023 The American Association for the Advancement of Science. (h) Magnetoreception: magnetotactic bacterium *Gammaproteobacteria* strain SHHR-1. Reproduced with permission from Li et al.^28^ Copyright 2020 American Geophysical Union, all Rights Reserved. (i) Storing nutrients: ferritin protein structure produced in PyMOL^29^ from the PDB2FFX X-ray crystal structure.^30^

In the search for biomineralization strategies that can be readily translated to synthetic systems, soluble organic additives have received the lion’s share of attention. Taking the example of calcium carbonate, many organisms precisely control the location, size, shape, orientation, and polymorph of the calcium carbonate biomineral that they form. Early work demonstrated that proteins associated with this biomineral are typically highly acidic and are rich in aspartic and glutamic acid residues.^31–34^ While the search for specific proteins that could control the crystal polymorph and uniquely generate either calcite or aragonite has proven challenging, these acidic proteins are invariably active in controlling crystal morphologies.^35–39^ Small quantities of proteins also become incorporated within crystals,^40^ significantly enhancing their hardness and fracture toughness.^13–15^ Control of crystal orientation, in turn, has often been attributed to organized organic matrices. These may act alone to direct crystal nucleation, or soluble biomolecules may be adsorbed on insoluble matrices, creating nucleation sites.^41–48^ Again, this strategy can be readily extended to synthetic systems, where Langmuir monolayers^49–51^ and self-assembled monolayers (SAMs)^52–54^ have proven highly effective in controlling crystal orientations. Yet, despite these successes, no soluble additive can generate morphologically complex, large-scale structures comparable to the skeletal elements of organisms such as echinoderms.^55,56^ It has also proven extremely difficult to achieve polymorph control ex vivo for calcium carbonate using soluble organic additives alone,^57–60^ indicating that organisms likely use additional control measures to achieve perfect fidelity in biomineralization.

Of course, these bioinspired strategies, which operate in bulk solution, offer very simplistic models of biomineralization processes. The organization and function of all organisms is based on compartmentalization such that they can achieve spatiotemporal control and orchestrate complex, multistep processes over multiple length scales.^61–63^ Compartmentalization allows the organism to define and isolate the site of mineral deposition and to safely sequester the high concentrations of ions required to create a supersaturated state.^64^ Given the recognition that confinement can have significant effects on crystallization processes,^65–67^ for example, stabilizing amorphous and metastable phases, directing crystal orientation, and templating crystal morphologies, it is expected that compartmentalization will also contribute to biogenic control over biomineralization processes. Indeed, the ability of organisms to control the size and shape of compartments and to translocate material across their bounding walls (for example by using passive pores or active ion pumps) promises far greater control than can be achieved in synthetic systems.

Here, we explore the role of confinement in biomineralization processes. The effects described range from those in which the contribution of confinement is immediately apparent, such as in the templating of complex crystal morphologies, to those that are far more speculative, such as control over polymorph. Indeed, studying the role of confinement in the formation of biominerals offers significant challenges. Ideally, we would wish to perform a time-resolved study that follows the development of a system from nucleation, through growth to the final product biomineral, and determine the relationship between the developing mineral phase and its surrounding organic matrix. This is extremely challenging, but advances in techniques such as cryogenic scanning electron microscopy (cryo-SEM),^8,68^ cryogenic transmission electron microscopy (cryo-TEM) and tomography,^69,70^ cryo-focused ion beam milling for 3D reconstruction,^71–73^ and X-ray ptychography^74,75^ are starting to allow high resolution 3D images of biominerals and their surrounding tissue to be recorded. Developments in correlating imaging and spectroscopic techniques^76^ are also adding key chemical information to these studies, as highlighted throughout this review.

We begin with a summary of the principal effects of confinement on crystallization processes. This information is gained from synthetic systems that facilitate systematic studies of crystallization in well-defined confined environments over length scales ranging from the nanoscale, such as in carbon nanotubes, through to hundreds of micrometers, as offered by droplet-based systems. This forms our foundation and illustrates both the effects of confinement on crystallization and the physical origin of these effects. We then consider how organisms exploit confinement in the creation of biominerals, where we principally focus on some well-studied examples that offer significant insight into biomineral structure and control strategies. These include the formation of calcium carbonate mollusk shells, echinoderm skeletal elements and coccoliths, calcium phosphate-based bones and teeth and silica diatoms and sponges. Comparatively simple organisms such as magnetotactic bacteria^77,78^ and diatoms,^79,80^ whose genetic codes have been sequenced, also provide a rich source of insight.

The potential roles of confinement in defining key structural characteristics of biominerals and their mechanisms of formation are considered in turn and are complemented by observations made on crystallization in confinement in relevant synthetic systems. As shown in Figure 2, we first discuss how confined volumes concentrate precursor ions before stabilizing and transporting poorly ordered/amorphous phases to the site of mineralization (Section 3) and then consider how confinement contributes to the morphological control of amorphous and crystalline biominerals (Section 4). Crystal orientation is covered in Section 5, and the potential routes by which organisms use confinement to select biomineral polymorph are discussed in Section 6. Finally, we address control over nucleation in Section 7, and highlight several systems, including the iron storage protein ferritin, that offer nanoscale reaction environments and are accessible for detailed characterization of biomineralization mechanisms.

**Figure 2:**
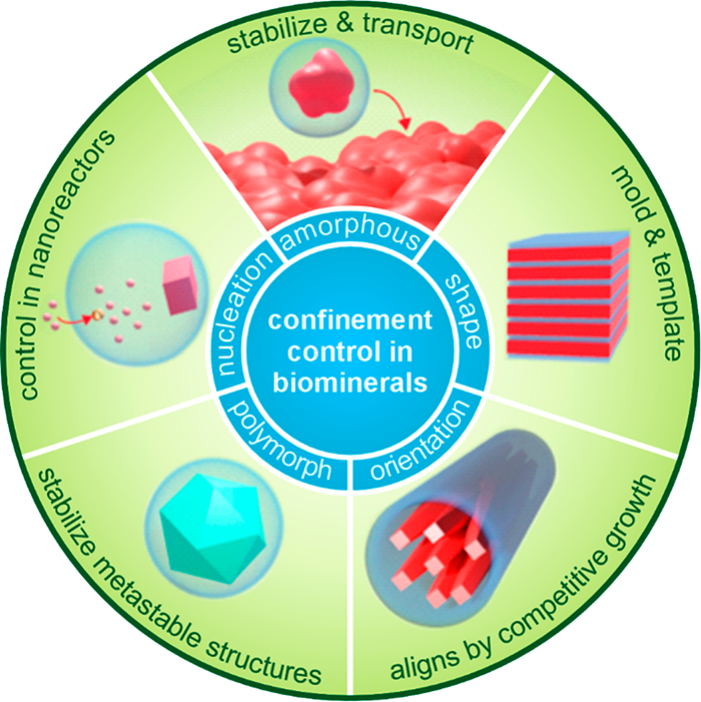
Illustration of the roles that confinement plays in biomineralization.

## 2 Effects of Confinement on Crystallization Processes

Confinement is now recognized to exert a wide range of effects on crystallization processes, where these can operate over length scales ranging from micrometer-scale to the nanoscale.^65–67^ We here provide an overview of the principal effects of confinement on crystal nucleation and growth, where this forms a foundation for the subsequent discussion of the role of confinement in biomineralization processes. An interested reader can find an in-depth treatment of this topic in other recent reviews.^65,66^

### 2.1 Nucleation

Nucleation in small volumes is generally associated with longer induction times due to a number of factors. (i) As a kinetic effect, the probability of a nucleus forming within a given time depends on the total volume of the supersaturated medium.^81–83^ If a volume is divided into an array of small droplets, then the probability that an individual droplet contains a crystal after time *t* is given by

1 $$P\left(t\right)=1-\exp\left(JV_{d}N_{d}t\right)$$

where *J* is the nucleation rate, *V*_d_ is the droplet volume, *N*_d_ the number of droplets, and *t* is the time.^82^ The time taken to observe nucleation within a droplet is therefore inversely dependent on the volume, such that a 10-fold reduction in the droplet diameter reduces the mean nucleation time by a factor of 10^3^. (ii) If crystallization occurs within an isolated finite small volume, then the nucleation of a crystal will be accompanied by a continual depletion of the supersaturation.^84–88^ This causes a concomitant reduction in the driving force for the formation of a critical nucleus, where the size of a critical nucleus, *r*_c_ is related to the supersaturation *S* as

2 $$r_{c}=\frac{2\gamma V_{m}}{kT\ln S}$$

where the molecular volume in the bulk crystal is *V*_m_, γ is the interfacial free energy, *k* is the Boltzmann constant, and *T* is temperature. The size of a critical nucleus that the system needs to achieve therefore increases as the supersaturation decreases, such that the system can continually “chase”, but ultimately fail to achieve nucleation.^85^ This thermodynamic effect can therefore prevent nucleation or limit the size that a crystal nucleus can achieve. Biology can overcome this isolation with pumps and pores that allow materials to cross the boundaries of confined systems so that chemical balance can be controlled or maintained as minerals nucleate and grow.

(iii) The formation of small volumes excludes impurities that promote nucleation in bulk solutions, and their removal leads to longer induction times in small volumes.^89^ This effect is often seen when crystallization is studied in an array of droplets, where a bimodal distribution of induction times corresponds to droplets that contain or do not contain impurities.^90^ (iv) As a final effect, modeling studies have shown that nucleation can be enhanced in confined environments with specific geometries.^91,92^ Simulations of the nucleation of solids with face-centered cubic (FCC) structures in atomically sharp wedges showed that nucleation rates vary according to the wedge angle and that they are significantly enhanced when an FCC crystal bounded by {111} planes ideally fits within the wedge.^91^

### 2.2 Polymorph

Confinement can also affect the crystal polymorph, where metastable phases can be significantly stabilized in small volumes^93–95^ and the crystallization pathway can be altered.^96^ This results in the formation of polymorphs that would not be produced from a bulk solution of the same composition.^94,97,98^ Notably, although the polymorph must be determined at nucleation, these effects can be observed at length scales that are orders of magnitude greater than those of a critical nucleus.^99^

The effects of extreme confinement on crystallization have been evaluated using carbon nanotubes, where diameters of just a few nanometers can approach the dimensions of lattice parameters.^100,101^ Simple inorganic compounds precipitated within these environments often exhibit distorted structures, polymorphs that differ from those formed in bulk control experiments, or novel crystal structures.^102–105^ The interactions of the atoms within the nanotube with the nanotube wall and the melt outside the nanotube have been shown to contribute to these effects.^106–108^ Multiple experiments have addressed the crystallization of organic compounds within small volumes, such as in the cylindrical pores of polymer membranes or in nanoporous glasses. These have revealed that polymorph varies with pore size^67^ and that amorphous phases (lacking long-range order) often form in the smallest pores.^109,110^ This has been attributed to the pores being too small to accommodate the critical nuclei of crystalline phases in some systems,^110,111^ as different polymorphs have different sizes of critical nuclei. Data have also suggested that the stabilization of metastable polymorphs may be thermodynamic in origin in some systems^112^ but due to slow kinetics in others.^111^

Notably, similar effects are seen in comparable experiments with soluble^93^ or sparingly soluble^113–116^ inorganic compounds. However, the influence of confinement on the polymorph of sparingly soluble inorganics is poorly understood, where critical nuclei are far smaller than the confined volume, so size exclusion cannot contribute to polymorph selectivity. Factors that could contribute to polymorph selectivity include the buildup of high supersaturations in impurity-free confined systems and reduced rates of transformation to more stable polymorphs. Reduced flux rates in confinement can contribute to the latter effect.^117,118^ The ratio of surface to volume also increases as the confining volume becomes smaller, such that the surface of the confining medium could play an increasing role in polymorph selection.^98^ The surface area to volume ratio increases as volume decreases with increasing confinement, so it is expected that the influence of the surface on controlling mineralization increases with increasing confinement.

Systems that offer extreme confinement can also select for the most stable polymorph. Stable polymorphs are usually associated with smaller critical nuclei than their metastable counterparts. A confined volume containing a fixed number of ions can therefore prevent the formation of metastable polymorphs if there are insufficient ions present to generate critical nuclei. This phenomenon has been observed for the crystallization of mefenamic acid, glycine, and ROY (5-methyl-2-[(2-nitrophenyl)­amino]-3-thiophenecabonitrile) within microemulsions.^86,119,120^

### 2.3 Morphology

The precipitation of crystalline materials within the confines of suitable templates is a highly effective means of controlling the morphologies of both polycrystalline materials and single crystals.^12,121,122^ This approach is quite general and is effective over multiple length scales, provided that the experimental conditions generate crystals or assemblages whose sizes exceed the dimensions of the template. The morphology can then be molded by the template and can even generate single crystals with complex, noncrystallographic morphologies and curved surfaces.^123,124^ Considering that nonbiogenic crystals generally exhibit geometric forms that reflect the underlying symmetry of the crystal lattice, organisms often go to great lengths to construct elaborate, nonequilibrium morphologies. Specific examples from magnetosomes, mollusks, coccoliths, echinoderms, chitons, and stomatopods are discussed in Section 4.

### 2.4 Orientation

Crystallization in confinement often gives rise to preferred orientations, especially when compounds with anisotropic crystal structures form within anisotropic environments.^98,125–129^ This has been most observed when crystals are grown within cylindrical pores, such as those found in porous anodic aluminium oxide membranes.^130,131^ This is usually attributed to competitive growth effects, where crystals oriented with their fast-growing directions parallel to the long axis of the pore grow at the expense of those oriented in other directions.^126–128^ Favorable interactions between specific crystal planes and the pore walls may also give rise to preferred orientations.^118,132^

### 2.5 Stabilizing Amorphous Phases

A number of in vitro experiments have demonstrated that amorphous phases can be stabilized by confinement.^99,113–115^ Amorphous phases are extensively used in biomineralization processes, either as precursors—an example being the transformation of amorphous calcium carbonate (ACC) into calcite spicules in sea urchin larvae^133,134^ —or as product biominerals in their own right, such as the siliceous cell walls of diatoms and in certain sponges.^135–138^ These amorphous phases may facilitate rapid mineralization, and it has been suggested that they can be sculpted more readily into nonequilibrium mineral morphologies.^139^ Confinement offered by organic structures such as vesicles is key to stabilizing and controlling the formation of amorphous minerals in biology.

## 3 Amorphous Biominerals

A significant number of biominerals are amorphous (i.e., they lack long-range order). These include minerals that will never crystallize (e.g., silica), those that act as precursors to crystalline phases (e.g., ACC and amorphous calcium phosphate (ACP)), and those that are stabilized against transformation by the organism (e.g., ACC). As precursors, amorphous phases are often employed as ion stores that can be rapidly formed and dissolved as required, facilitating high mineral growth rates and making organisms more responsive to changes in their environment. For example, abundant vesicles containing calcium-rich, electron-dense granules are seen in the intramolt stage in crustaceans.^140,141^ During intramolt, calcium is resorbed from the exoskeleton prior to a molt during ecdysis and redistributed to the newly mineralizing carapace.^142^ ACC also acts a precursor to calcite in avian eggshells, where it facilitates extremely rapid growth rates of ≈6 g of CaCO_3_ in just 18 h when chickens mineralize their eggshells.^143–145^

Amorphous biominerals are frequently used as direct precursors to crystalline biominerals. Ion-by-ion growth was long considered the dominant mechanism by which crystals formed in organisms, where it was envisaged that the constituent anions and cations were transported to and then concentrated within the compartment in which mineralization occurs.^146^ Observations made of spiculogenesis in sea urchin larvae in the late 1990s challenged this view and demonstrated that ACC is concentrated within the spicule compartment before undergoing a pseudomorphic transformation to a single crystal of calcite.^133,134^ That ACP can act as a precursor to the formation of biogenic hydroxyapatite (HAp) was proposed as early as the 1960s,^147^ and was later confirmed for both bones^148^ and teeth.^134^ Evidence now suggests that organisms employ a range of processes, including ion-by-ion growth and the use of amorphous phases during biomineralization.^149–151^

Amorphous biominerals have also been implicated in the ability of organisms to control mineral morphologies, where their lack of preferred form makes them easy to mold. Silica is therefore an excellent choice for the fabrication of the intricate nanoporous structures of diatoms.^135,136^ Similarly, single crystals with noncrystallographic morphologies can be created following the pseudomorphic transformation of a shaped amorphous mineral,^152^ although in vitro experiments have shown that amorphous precursors are not required to template calcite crystals with complex morphologies.^153^ As a final example, amorphous and crystalline phases are often combined in a single biomineral, as observed in the calcium carbonate spicules produced by some ascidians.^154,155^ This can enhance fracture toughness as amorphous phases do not possess the cleavage planes that make many single crystals brittle.

The structures of amorphous minerals are hard to characterize, particularly if they appear in association with crystalline phases.^156,157^ For example, biogenic ACC varies in its degree of hydration, where ACC that acts as a precursor to the crystalline polymorphs (transient ACC) often has a low water content, while long-lived ACC (stable ACC) is hydrated and has a typical composition of CaCO_3_·H_2_O.^158,159^ ACC is distinct from widely observed ion-rich granules that contain high concentrations of organic molecules and ions such as calcium or phosphate, and from polymer induced liquid precursor (PILP) phases that form in the presence of low concentrations of polyelectrolytes. PILP phases exhibit some liquid-like properties, and the possibility that they participate in biomineralization processes has been widely discussed.^152^ Many questions therefore remain regarding the mechanisms by which organisms employ amorphous materials in biomineralization.

This section discusses the role of confinement in generating and stabilizing amorphous phases in biomineralization, transporting them to the site of mineralization, molding them, and controlling their transformation (if the product biomineral is crystalline). Notably, amorphous particles may be added directly to the mineralization compartment, or this precursor material may dissolve, and the constituent ions are then transferred to the developing biomineral. To achieve this, organisms must first concentrate the constituent ions to levels well above those found in their local environment, prevent these from crystallizing until they are delivered to the mineralization site, and control the transformation of the amorphous phase. We first focus on the well-studied calcium carbonate and calcium phosphate systems, using synthetic systems to illustrate the effects of confinement on the formation and stabilization of ACC and ACP and on the behavior of PILP phases, before then considering biomineralization processes. Similar confinement-based mechanisms are then described in organisms that produce amorphous silica.

### 3.1 ACC and ACP in Synthetic Systems

ACC and ACP readily precipitate from bulk solution and are often seen as precursors to the crystalline polymorphs of calcium carbonate and calcium phosphate when their solubilities have been exceeded. ACC is typically short-lived in bulk aqueous solution^152,160^ but can be stabilized using inorganic ions such as magnesium or phosphate,^161^ organic additives such as citric acid,^161–163^ or by washing in ethanol.^164^ These inorganic ions principally inhibit the nucleation and growth of the more stable crystalline phases, which increases the lifetime of the ACC. ACP is generally more stable than ACC in aqueous solution.^165^ Like ACC, the conversion of ACP into crystallites can be delayed by inorganic ions (e.g., Mg^2+^),^166^ organic additives (e.g., adenosine triphosphate),^167^ or by washing in ethanol.^168^ A further feature of ACP is that it often forms ultrasmall clusters with diameters of only ≈ 0.9 nm (Posner’s clusters),^169,170^ while ACC usually exists in the form of colloidal particles with sizes ranging from 20 to 200 nm.^171,172^

Confined environments can significantly stabilize amorphous precursor phases in vitro. An annular wedge created between two crossed cylinders was used as a crystallization environment, where the surface separation varies with the distance from the contact point of the cylinders (Figure 3a). Precipitation of calcium carbonate in this apparatus resulted in the formation of ACC at submicrometer surface separations that was stabilized for days (Figure 3b),^99^ while the lifetime of ACP was extended by an order of magnitude in comparable experiments with calcium phosphate at surface separations of 200 nm.^114^ This system is open and confinement is provided normal to the surfaces only. Amorphous precursors of calcium sulfate^113^ and calcium oxalate^115^ have been stabilized in confinement in the same system, which is indicative of a common effect. Reduced material transport between the amorphous precursor and any growing crystal nucleus may contribute to the retardation of crystallization.^65^

**Figure 3:**
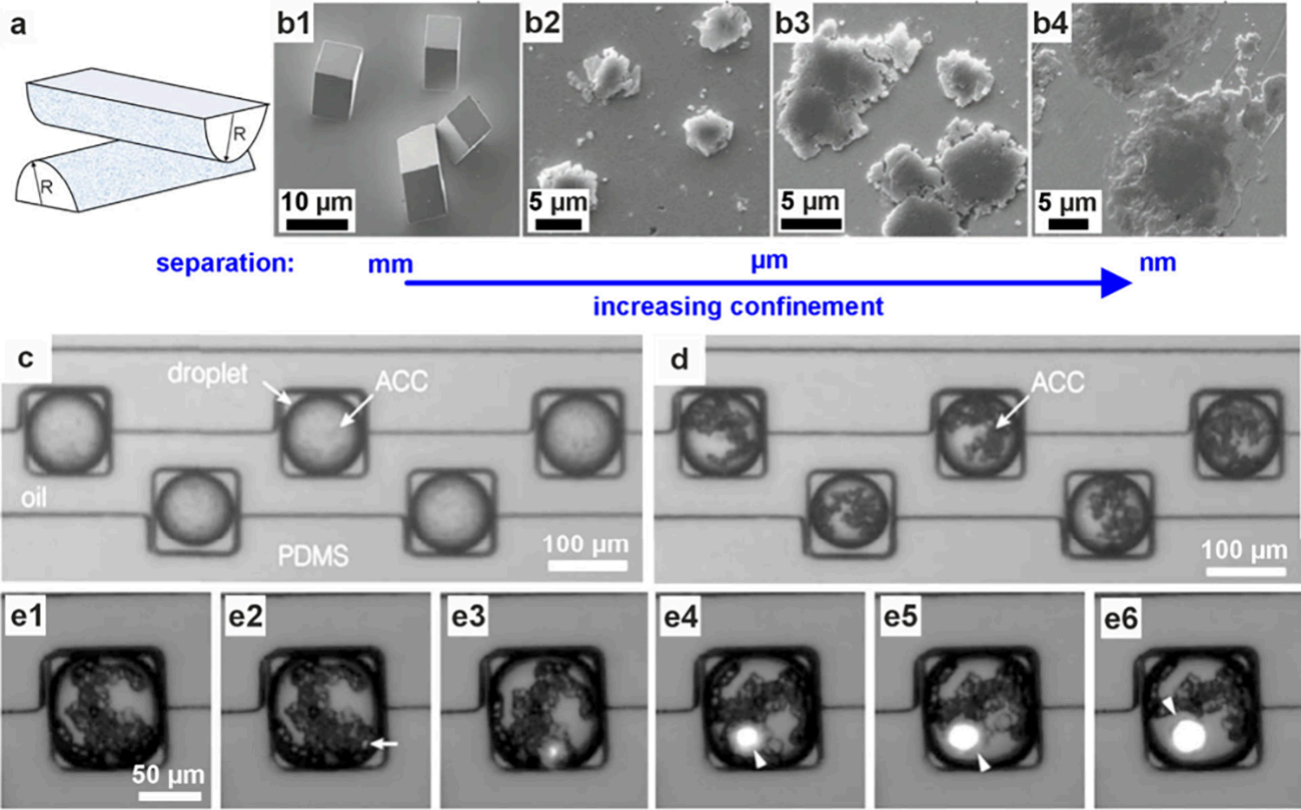
**ACC formation in confinement in vitro.** (a) Sketch of crossed cylinders with radius *R*. (b) SEM images of calcium carbonate precipitated between crossed cylinders at surface separations of (b1) >1 mm, (b2) 10 μm, (b3) 2 μm, and (b4) 500 nm, showing that increasing confinement forms particles that were more amorphous. (a, b) Reproduced with permission from Stephens et al.^99^ Copyright 2010 WILEY-VCH Verlag GmbH & Co. KGaA, Weinheim. (c–e) Optical microscopy images of a “droplet orchard” device, showing ACC precipitated when droplets containing CaCl_2_ solution were exposed to (NH_4_)_2_CO_3_ solution after (c) 5 min and (d) 25 min. (e) The transformation of ACC to a birefringent crystal (arrows) (e1) at 14 h 20 min and subsequent images at 5 min intervals. Reproduced with permission from Cavanaugh et al.^173^ Copyright 2019 The Author(s) & The Royal Society of Chemistry with a CC-BY-NC-3.0 license.

Closed systems such as vesicles also offer significant stabilization of amorphous phases.^174–176^ This can be attributed to the low probability of nucleation in small volumes^99,114,115,173,175^ and the elimination of impurities that can act as heterogeneous nucleators.^177^ Experiments were conducted by immersing 100–1000 nm^174,175^ and 20–50 μm diameter^176^ liposomes encapsulating 1 M calcium chloride solution in 1–1.5 M ammonium carbonate solution, generating ACC within the vesicles. No crystallization was observed in the giant 20–50 μm vesicles within 8 days, but immediate transformation to calcite was observed when the vesicles were lysed with detergent.^176^ The crystallization of ACC within droplet environments was also investigated using 100 μm droplets created in a microfluidic device and stored in a “droplet orchard”,^173^ where crystals formed in only 16% of the droplets over 100 h (Figure 3c–e).^173^

These phospholipid liposomes and droplets offer effective mimics of the vesicles that encapsulate ACC or ACP in organisms, although it is noted that they will contain very high concentrations of counterions. This will not be the case in biology, where organisms can precisely regulate ionic concentrations using ion pumps.^178^ Indeed, vesicular confinement has been implicated in creating high concentrations of Mg, Ca, Sr, and Ba in cyanobacteria in order to stabilize amorphous carbonates.^179,180^ Organisms can also completely fill vesicles with an amorphous precursor,^145^ which is again not yet possible in synthetic vesicles. Coating ACC with a surfactant layer, and reducing free solution is again likely to stabilize ACC by preventing it from dehydrating and undergoing dissolution/reprecipitation.^181^

### 3.2 Polymer-Induced Liquid Precursor (PILP) Phases

Precipitation of calcium carbonate and calcium phosphate in the presence of low concentrations of charged polyelectrolytes such as poly­(acrylic acid) (pAA),^152,182^ poly­(aspartic acid) (pAsp),^183,184^ poly­(allylamine hydrochloride) (pAH),^185,186^ double-stranded deoxyribonucleic acid (DNA),^187^ and proteins including alkaline phosphatase^188^ and ovalbumin^189^ can result in the formation of a dense, liquid-like phase that is intermediate in ion concentration between an ionic solution and an amorphous solid (Figure 4a). This has been termed a PILP phase,^152,183^ and it exhibits properties characteristic of a liquid, such as film-formation and infiltration into confined spaces.^190,191^ Calcium phosphate PILP shows some significant differences to calcium carbonate PILP, where for example it does not form thin films on substrates.^191^ Interestingly, both calcium carbonate and calcium phosphate PILP phases appear to contain 1–2 nm nanoclusters when visualized using cryo-TEM, suggesting that they are collections of small ACC or ACP clusters stabilized by polyelectrolytes (Figures 4b).^192–194^ However, when visualized using liquid-phase TEM at a ≈10 nm resolution, calcium carbonate PILP behaves like flowing liquid droplets.^195^

**Figure 4:**
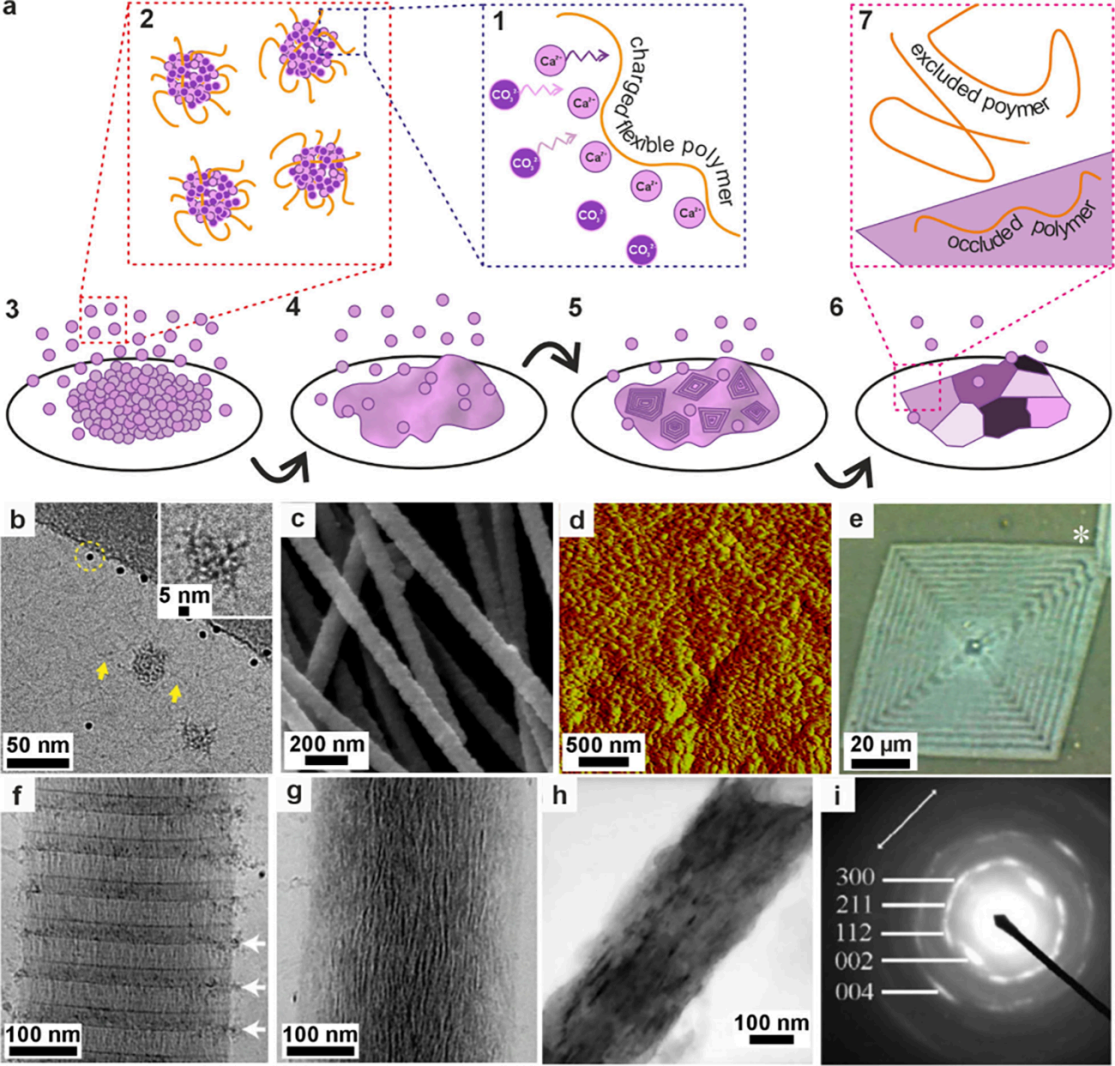
**The formation and crystallization of a polymer-induced liquid precursor (PILP).** (a) Illustration of the PILP process for calcium carbonate formation. Adapted in part with permission from Gower and Odom^183^ Copyright 2000 Elsevier Science B.V., all rights reserved. Adapted with permission from Xu et al.^192^ Copyright 2018 of the Author(s) and Springer Nature with a Creative Commons Attribution CC-BY 4.0 license. (a1) A negatively charged polymer complexes with positively charged calcium, which in turn attracts carbonate. (a2) These condense into ≈2 nm diameter clusters of ACC, then these smaller particles gather to form 30–50 nm intermediate diameter clusters, which then agglomerate further into micrometer-sized particles. (a3) The particles accumulate on substrates and (a4) coalesce to form continuous films. (a5) Crystals nucleate within the film and (a6) grow until the entire film is crystalline. (a7) The polymer can be excluded from the crystal or occluded within it. (b) Cryo-TEM images of double-strand DNA-induced calcium carbonate PILP. Reproduced with permission from Xu et al.^192^ Copyright 2018 of the Author(s) and Springer Nature with a Creative Commons Attribution CC-BY 4.0 license. (c) SEM image of calcite particles precipitated in the pores of track-etched (TE) membranes with calcium carbonate PILP. Reproduced with permission from Kim et al.^190^ Copyright 2011 WILEY-VCH Verlag GmbH & Co. KGaA, Weinheim. (d) Atomic force microscopy (AFM) image showing the nanogranular texture of a calcite thin film transformed from pAA calcium carbonate PILP on a COOH-terminated SAM. Reproduced with permission from Kim et al.^196^ Copyright 2007 American Chemical Society. (e) Polarized light micrographs of CaCO_3_ crystals grown in pAsp calcium carbonate PILP ACC film, showing transition bars. Reproduced with permission from Dai et al.^197^ Copyright 2008 American Chemical Society. (f–g) Cryo-TEM images of collagen after mineralization in vitro with calcium phosphate PILP with pAsp. Reproduced with permission from Nudelman et al.^193^ Copyright 2010 Springer Nature Limited. White arrows in (f) highlight nanoclusters. The mineral infiltrates the collagen, which becomes more crystalline, as shown in (g) after 72 h. (h) TEM image of a collagen fibril mineralized in vitro using a calcium phosphate PILP and (i) SAED image of the mineralized fibril indexes to HAp aligned with the fibril. Reproduced with permission from Olszta et al.^184^ Copyright 2007 Elsevier B.V., all rights reserved.

The ability of PILP to infiltrate into small volumes has been demonstrated in experiments with track-etched (TE) membranes, whose cylindrical pores offer well-defined crystallization environments with pore diameters ranging from 10 nm to tens of micrometers (Figure 4c). Comparison of crystallization carried out (i) via an ACC phase formed in the absence of additives and stabilized at 4 °C and (ii) in the presence of a PILP phase formed with pAA or pAsp showed that intramembrane crystals were generated in the vast majority of 200 and 50 nm pores using the PILP phase, as compared with 50% (200 nm pore) and 10% (50 nm pores) using the additive-free ACC.^190^ Further evidence for PILP facilitating infiltration into confinement was also provided when the 200 nm membrane pores were sealed at one end only. In this case, limited infiltration occurred, generating a thin layer coating the internal pore walls that resembled a liquid rising up a tube by capillary action.^192^ A number of in vitro experiments have shown that calcium phosphate PILP can infiltrate into collagen fibrils before transforming to crystalline HAp.^184,193,194^ This leads to mineralized fibrils with structures and mineral alignment comparable to those found in native bone (Figure 4f–i)^184^ and Section 5.2.1.

There has been significant discussion about the potential role of PILP phases in biomineralization processes, but no definitive proof of their existence has yet been obtained. However, a range of observations provide indirect evidence. For example, calcite platelets formed on transformation of PILP films often possess a granular texture (Figure 4d),^196^ and they can show transition bars (Figure 4e) that are formed by diffusion-limited exclusion of the polymeric impurity from the crystal,^183^ where these delineate sectors within single crystals or appear as concentric rings within spherulitic films.^197^ Similar features are often observed in calcium carbonate biominerals such as nacre or coral.^152,184,198–200^ A dense, liquid-like material consistent with a PILP phase has also been observed in the pores at the repair site of regenerating sea urchin spines.^201^ Finally, biomolecules such as Pif80 proteins from mollusks have been shown to complex with calcium ions to form coacervates in vitro.^202^ These are destabilized when exposed to the salt levels found in extracellular fluid or seawater, indicating that the calcium-concentrating biomolecules need to be isolated from the extracellular medium, probably inside vesicles, in order to form stable PILP complexes.

### 3.3 Calcium Carbonate Crystallization via Amorphous Precursors

Confinement plays a key role in the formation of amorphous calcium carbonate precursors in vivo and their transport to the site of mineralization. In marine organisms such as sea urchin larvae^203^ and foraminifera,^204–206^ calcium and carbonate/bicarbonate ions are obtained by the invagination of seawater into larger vacuoles, and the calcium ions are then concentrated to 1–15 M Ca^2+^ in smaller vesicles within these vacuoles.^75,207–210^ This concentration is 2–3 orders of magnitude higher than found in seawater (≈10 mM Ca^2+^) but lower than hydrated ACC (19 M) or calcite (27 M).^75^ Alternatively, ions are obtained from the environment and concentrated within vesicles using ion channels and pumps, as observed in the formation of hen eggshell, where carbonic anhydrase catalyzes the formation of bicarbonate ions from carbon dioxide.^145^

Vesicles containing amorphous precursors to calcium carbonate have been observed in many organisms, where they localize high concentrations of calcium ions, isolate the amorphous material from the rest of the organism, and stabilize it against crystallization. Current imaging and characterization techniques are often unable to rigorously characterize these amorphous materials. For example, it is not possible to definitively distinguish between ACC and calcium-rich dense phases in the ≈25 nm puncta that are observed near to the coccolith deposition vesicle in *Pleurochrysis carterae* and *Hymenomonas carterae*.^211,212^ The calcium-rich dense phases are considered to be complexes of calcium ions and coccolith-associated polysaccharides (CAPs). The CAPs are proposed to facilitate the formation of a dense, Ca-rich precursor phase that plays a key role in the transportation of calcium ions to the mineralization site and to regulate the abundance and solubility of this phase.^73^ These dense precursors appear to dissolve adjacent to the crystallization compartment, releasing calcium ions for crystal growth.^73,151^

So far, vesicles containing ACC have only been unambiguously detected in bacteria,^213,214^ avian eggshells,^145^ mollusks^215,216^ and sea urchins,^203,207^ and it has been suggested that the granular ACC detected in corals may be stabilized in vesicles.^210,217^ The confinement offered by the vesicles acts in conjunction with soluble additives to stabilize the ACC, and the close proximity of the liposome membrane to the ACC surface limits dehydration and dissolution of the ACC, retarding its transformation to a crystalline phase.^181^

Delivery vesicles are thought to transport precursors to the mineralization site, where they could either come close to the site and individual ions can be transferred, or they could fuse with the mineralization compartment, releasing ACC particles to the growing crystal. Extracellular vesicles (EVs) fusing with target cells and organelles is a key mechanism underlying the delivery of cargos in biology.^218^ Proteomic, mRNA, energy dispersive X-ray (EDX), and electron energy loss spectroscopy (EELS) analysis of EVs in chickens showed that they contained ACC and were targeted to fuse to the egg mineralization site.^145,219^ Direct introduction of ACC to the mineralization compartment has been proposed for mineralization in sea urchin larvae^203^ and adult stony coral *Stylophora pistillata*, where X-ray photoemission electron spectromicroscopy (X-PEEM) and X-ray absorption near-edge structure spectroscopy (XANES) of adult coral skeletons suggested that ACC is stabilized in vesicles that are then transported to the surface of coral skeletons.^217^ These 400 nm particles attach to the coral surface, where they remain amorphous for hours before transforming into aragonite.^217^ Analysis of the structure of the mineralization front in another five coral species further supported this mechanism.^210^ This coalescence mechanism must occur despite soluble additives that stabilize the ACC being present, suggesting that a change in environment triggers crystallization of the ACC.^149^ ACC particles are expected to crystallize when they are added to an existing crystal, where this could potentially occur via secondary nucleation or localized dissolution/reprecipitation (where the crystal is less soluble than the ACC).

It is noteworthy that the ACC coaescence mechanism was observed near the center of calcifcation of the coral, which is enriched in Mg^2+^.^217^ In contrast, no evidence for dense Ca^2+^ storage compartments or ACC particles was found during the analysis of later biomineralization stages of the primary polyps of the coral *S. pistillata*.^220^ At this stage, the EVs seem to directly deliever sea water to the mineralization site and precipitate into micron-sized crystals, as suggested by cryo-SEM and EDX observations. This variation of mechanisms suggest that different pathways may operate at different stages of biomineralization.

In bulk solution, ACC typically crystallizes via a dissolution/reprecipitation mechanism such that the morphology of the product crystal bears no resemblance to the shape of the ACC precursor aggregates.^221^ As an unusual exception, ACC containing magnesium ions was observed to transform to calcite with shape preservation under certain experimental conditions.^222^ However, both modern and ancient calcium carbonate biominerals often exhibit nanoparticulate ultrastructures, which are indicative of amorphous precursor nanoparticles, and a solid-state transformation rather than full dissolution/reprecipitation.^198,223,224^ This gives rise to a so-called “mesocrystal” structure, where the mineral diffracts as a single crystal but exhibits a defined nanoparticulate ultrastructure. This is seen, for example, in avian eggshells, where large quantities of ACC are delivered to and accumulate at the sites of mineralization^143,145^ before transforming to columnar calcite crystals that have granular ultrastructures.^143^ Comparable granular ultrastructures have been observed in vitro when calcite crystals were grown from ACC nanoparticles in the presence of biomolecules extracted from the sea urchin *Paracentrotus lividus*.^225^ Particulate aragonite structures were also detected in corals, which are thought to arise from the attachment of ACC nanoparticles to the crystal growth front,^217,225^ and an ion-by-ion growth mechanism filling in the space between the particles.^210^

Biomineralization from ACC precursors has been studied in the most detail in sea urchin larvae. These are excellent model systems for studying biomineralization, as the eggs are readily fertilized in vitro and their development (including the initial stages of biomineralization) can be observed in real time using optical microscopy. Spicule formation occurs within a membrane-bound compartment (a syncytium) and begins with the formation of a faceted single crystal of calcite (see Section 4.3.3).^133,207^ Spicule growth is achieved by delivery of ACC particles to the mineralization site,^75^ and continues by the development of rod-shaped outgrowths to generate a triradiate spicule.^133^ The spicule remains within the confines of the syncytium throughout its growth,^207^ and no free liquid has been observed between the organic membrane and the developing spicule. This ACC then transforms to calcite without a loss in volume.^208^ No defined crystallization front is observed, and the transforming spicule contains domains of amorphous and crystalline material, which is consistent with the crystallization front propagating via a tortuous pathway through secondary nucleation.^208^

In sea urchin larvae, calcium ions for biomineralization are concentrated within primary mesenchymal cells (PMCs).^209^ Sea water enters the PMCs in large vacuoles and vacuolar networks that are subsequently transformed into vesicles.^203^ Cryo-SEM showed that the PMCs contain 0.5–1 μm electron dense vesicles packed with 20–30 nm calcium carbonate nanoparticles,^203^ whose appearances are consistent with ACC (Figure 5a–c).^225^ Similar electron-dense structures were observed in freeze-fractured samples^207^ and cryo-soft X-ray transmission microscopy,^75^ although a surrounding membrane was not always observed. The contents of these calcium-rich bodies vary in crystallographic order from levels comparable to seawater, through hydrated ACC, to the more ordered dehydrated ACC, demonstrating a continuum in composition as the precursor ions are converted to ACC (Figure 5c).^75^ This solid-state material is then likely transferred from the PMCs to the syncytium, possibly by fusion of vesicles to the syncytium membrane.^203,207,226^ Calcium-rich vesicles undergo slower active diffusion when the vesicles near the spicule deposition site in the skeletogenic tissues in echinoderms, which is assumed to be due to the binding or fusing of the vesicles to deliver their mineralization cargos.^226^ Here, fully dehydrated ACC is formed before undergoing a solid-state secondary nucleation process to transform to calcite at the forming spicule.^55,203,207,208^ It has also been postulated that ACC crystallization may be facilitated by enzymatic degradation of stabilizing macromolecules.^207^

**Figure 5:**
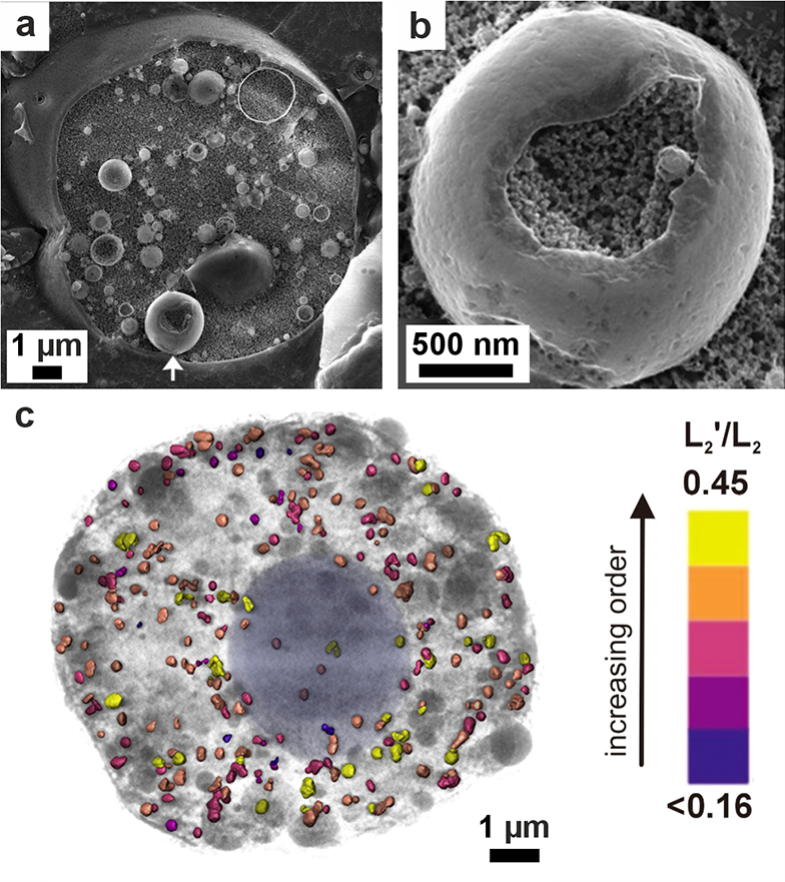
**Vesicles containing amorphous precursors in sea urchin embryos**. (a, b) SEM images of freeze-fractured embryos ≈40 h post fertilization. Reproduced with permission from Vidavsky et al.^203^ Copyright 2014 National Academy of Science under the PNAS license. (a) Fractured PMC containing large vesicles/vacuoles adjacent to a spicule labeled *s*. (b) Vesicle (labeled with an arrow in (a)) containing 20–30 nm diameter nanospheres. (c) PMC plunge frozen at ≈36 h postfertilization reconstructed from a cryo-soft X-ray tomogram. Color is greyscale with the nucleus shaded blue, then overlaid with Ca particle L_2_′/L_2_ peak intensity ratios from Ca L_2,3_ edge XANES spectra. This ratio is used as a proxy for the degree of order found in the calcium ions (low = disordered, high = more ordered). There is no L_2_′ peak in seawater, and the ratio for hydrated ACC is ≈0.26, ≈0.40 for anhydrous ACC, and ≈0.45 for calcite. The Ca particle L_2_′/L_2_ peak intensity ratios of the vesicles are randomly distributed throughout the cell.

### 3.4 Stable Amorphous Calcium Carbonate Biominerals

It is also valuable to consider ACC that shows long-term stability in vivo. This has been observed in a number of organisms, where the ACC can appear as the unique component of biominerals^155,227^ or in combination with crystalline calcium carbonate^155,227,228^ or other minerals.^229^ Examples include the dogbone spicules generated by the ascidian sea squirt *Pyura pachydermatina*, which comprise an ACC core and calcitic envelope,^154^ and the cuticles of some crustacea, where calcite is present in the outer exocuticle and ACC within the endocuticle.^230,231^ The triradiate spicules produced by the calcareous sponge *Clathrina* comprise a calcite core and ACC outer layer and appear to be covered by a thin calcite coating.^155,227^

There is strong evidence that the long-term stability of biogenic ACC is associated with the presence of macromolecules and additional inorganic ions including magnesium and phosphate.^274,901^ Proteins associated with ACC are typically glycoproteins rich in hydroxyamino acids and glutamic acids, while those extracted from crystalline calcium carbonate phases are characteristically rich in aspartic acid and glutamic acid.^154^ However, confinement has also been suggested to contribute to the stability of the ACC in these mixed-phase biominerals,^154,229^ where an organic sheath separates the ACC and calcite layers in ascidian dogbone spicules, potentially stabilizing the ACC against transformation.^154^ That the stable ACC component of plant cystoliths crystallizes in the presence of water further suggests that the environment in which the ACC is located can enable the organism to control the activity of water and thus the stability of the ACC.^229^

The formation of ACC by micro-organisms has also attracted significant interest, where bacteria and slime molds have been shown to induce the formation of extracellular calcium carbonate.^232–236^ Some of these generate ACC,^232,237^ whose stability has been attributed to extracellular polymeric substances that coat the ACC and retard its transformation.^237^ More interesting are observations of intracellular ACC in both prokaryotes and eukaryotes, where it is now recognized that a broad phylogenetic diversity of bacteria affiliated with *Cyanobacteria*,^238–241^ *Alphaproteobacteria*,^213,242,243^ and *Gammaproteobacteria* ^244^ can generate intracellular ACC particles a few hundred nanometers (and sometimes larger) in size that can occupy up to two-thirds of the total cell volume.^245^ The ability of some magnetotactic bacteria (*Gammaproteobacteria* ^244^ and *Alphaproteobacteria* ^242^) to form both magnetite and ACC is particularly interesting. Intracellular ACC inclusions enriched with strontium and barium have also been observed in green algae.^246–248^

The mechanisms by which these organisms produce intracellular ACC are poorly understood. The ACC masses are generated under conditions where the extracellular solution is undersaturated with respect to ACC, demonstrating that the process is associated with an energy expense.^245^ As further evidence that the ACC mineralization occurs under biological control, two different distribution patterns of ACC particles are observed within cyanobacteria.^238^ They are either distributed throughout the cytoplasm (Figure 6a) or located at the cell poles (Figure 6b),^238^ and the relationship between the calcium concentration in the extracellular solution and the rate of production of ACC is complex.^249^ Application of comparative genomic techniques to ACC biomineralization in cyanobacteria has also identified a new gene (*ccyA*) and protein family (calcyanin) possibly associated with these ACC storage processes.^240^

**Figure 6:**
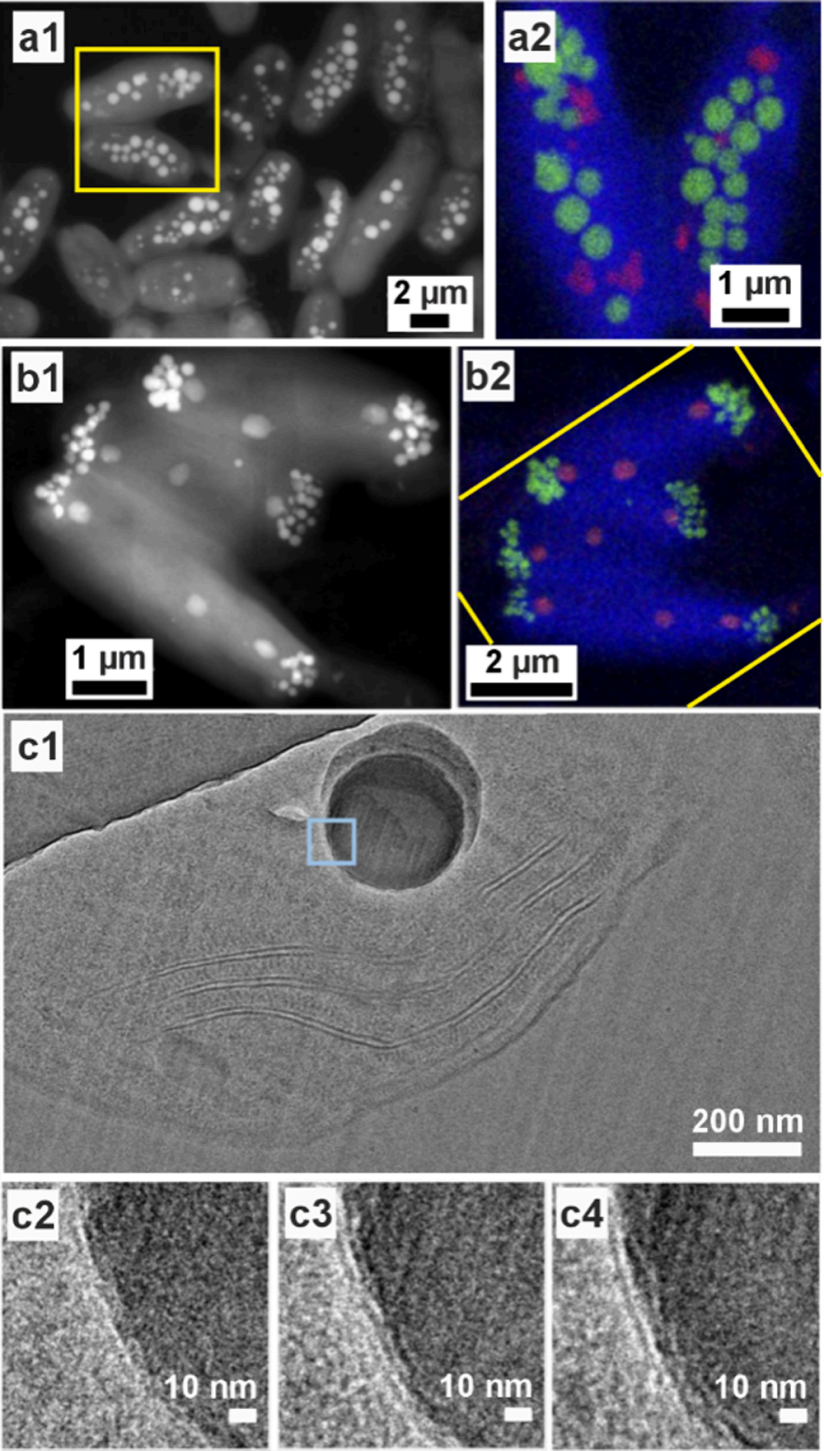
**ACC in cyanobacteria**. (a, b) STEM images and STEM-EDX elemental mapping of cyanobacterial species. Reproduced with permission from Benzerara et al.^238^ Copyright 2014 National Academy of Science under the PNAS license. Images show (a) *Cyanothece sp*. PCC 7425 (from a Senegalese rice paddy^250^) with ACC distributed throughout the cell and (b) *Candidatus Synechococcus calcipolaris* strain G9 (from Lake Alchichica, a highly alkaline and saline volcanic crater lake) with ACC particles concentrated at the cell poles. Yellow boxes outline the same area on the (1) STEM image and (2) STEM-EDX elemental map (blue carbon, red phosphate, green calcium). (c) Cryo-TEM image of a vitreous section (CEMOVIS) of an ACC inclusion in the cyanobacterium *Gloeomargarita lithophora*. Reproduced with permission from Frontiers in Microbiology, Blondeau et al.^214^ Copyright 2018 The Author(s) with Creative Commons Attribution CC-BY license. Insets below are of the pale blue marked area at different defocus heights (−1, −2, and −3 μm are (c2), (c3), and (c4) respectively) to highlight the organic structure around the ACC particle.

Imaging techniques have demonstrated that the ACC particles form within microcompartments that may contribute to their stability.^245^ Cryo-TEM of organisms that had undergone high-pressure freezing followed by cryo-ultramicrotomy of vitreous sections (CEMOVIS) revealed that the ACC inclusions formed by six cyanobacteria strains were coated by a 2.5 nm thick organic layer that was consistent with a protein shell (Figure 6c).^214^ This is comparable to the dimensions of a protein structure like a carboxysome or a lipid monolayer, as opposed to the lipid bilayer associated with magnetite crystals in magnetotactic bacteria,^214^ which is typically thicker at about 4–7 nm.^251^ Interestingly, similar phospholipid monolayers are often observed surrounding lipid droplets in eukaryote cells or polyhydroxyalkanoate inclusions in prokaryote cells, suggesting that the ACC inclusions may have PILP-like characteristics.^214^ In contrast, similar analysis of the ACC deposits formed within a magnetotactic bacterium showed that the particles were surrounded by a lipid bilayer and thus that they formed within membrane-delimited vesicles.^213^ Given the relative simplicity of unicellular organisms, these systems offer a great opportunity for further investigations into the mechanisms underlying the formation and stabilization of intracellular ACC.

### 3.5 Calcium Phosphate Biomineralization via Amorphous Precursors

ACP has been identified as the precursor to the major mineral component of bones and teeth^134,148,252–257^ and transforms to nonstoichiometric HAp doped with carbonate and other ions.^258–260^ Most mammalian biofluids (plasma, saliva, urine etc.) are supersaturated with respect to HAp^261^ but contain crystallization inhibitors such as mineral-binding proteins and polyphosphate, which inhibit HAp formation^262–264^ and thus stabilize ACP.^261^ Confinement is therefore not required to generate or stabilize ACP in mammals. However, membrane-bound vesicles are used to transport significant masses of ACP to the sites of mineralization, as seen during the formation of mouse bone and cartilage,^252,265^ zebra-fish fin rays,^253^ and chicken embryo bone.^266^ As of yet, ACP-bearing vesicles have not been identified in the formation of teeth, but the similarity in the composition and structure of dentin to bone suggests that they may be involved in mineral transportation.^267^

Chicken embryos contain micrometer-scale ACP-bearing vesicles in blood serum, as well as osteoblast and osteoclast cells (which are involved in bone formation and dissolution respectively).^266,268^ This indicates that ACP can form via the dissolution of bone minerals prior to its transportation to mineralization sites in vesicles, which minimizes the chance that blood vessels are hardened by pathological vascular calcification^266^ and prevents the organism from being exposed to toxic levels of calcium.^149^ Further insight into the mechanisms underlying ACP formation in vesicles has been obtained by culturing mouse osteoblast cells. These studies showed that ACP is loaded into vesicles by mitochondria^269^ and that this is initiated by the transportation of clusters containing calcium and phosphorus from the endoplasmic reticulum to the mitochondria.^270^ The Ca:P ratio of the ACP in the vesicles is lower than in bone HAp,^252,254^ and the ACP also contains polyphosphate,^252,271^ adenosine triphosphate,^167,272^ and acidic proteins,^273,274^ all of which are thought to stabilize ACP against crystallization. The ACP-containing-vesicles are then exocytosed from the cell as matrix vesicles^269,270,275–277^ that transport the ions and biomolecules required for bone mineralization to the biomineral deposition site.^278^

ACP particles have also been found between collagen fibrils in the extracellular matrix of bone and tooth dentin,^253^ but it remains unclear how this ACP transforms to HAp (see Section 7.2). In vitro studies have successfully mineralized collagen fibrils in the presence of noncollagenous proteins such as fetuin^279^ and polyelectrolyte mimics like pAsp and pAH.^184,194^ While it is tempting to suggest that calcium phosphate PILP or polymer-stabilized ACP clusters infiltrate the collagen fibrils before transforming into HAp platelets,^184^ this has not been conclusively shown, and polyelectrolytes that are too large to infiltrate collagen fibrils can still facilitate intrafibrillar mineralization by inhibiting extrafibrillar crystallization.^194,279^ Further in situ studies such as high-resolution cryogenic electron tomography (cryo-ET) of the early stages of collagen mineralization are required to fully understand the role of polymer-stabilized ACP in the intrafibrillar mineralization of collagen.

### 3.6 Silica Biomineralization: A Stable Amorphous Biomineral

A number of organisms use amorphous silica to construct protective, structurally supportive architectures, including phytoplanktonic diatoms,^135,136^ hexactinellid and demosponge “glass” sponges,^137,138^ and multicellular plants such as rice and horsetail grasses.^280,281^ These organisms employ confinement to deliver biotemplating molecules and high concentrations of silicic acid precursors to the site of mineralization, to control the chemical composition of the compartment where mineralization occurs, and to shape the silica (see Section 4.1). We here consider how two model organisms—diatoms and silicifying sponges—use confinement to control the formation of amorphous silica.

#### 3.6.1 Silica Formation in Diatoms

Diatoms are abundant aquatic unicellular algae that are encased within porous silica structures—termed frustules—as part of their external cell walls (Figure 7).^282^ These exhibit remarkable morphologies, providing mechanical protection to the cell that can act as photonic waveguides to enhance the collection of light at the absorbance wavelength of chlorophyll.^283–285^ As unicellular organisms, diatoms are comparatively straightforward to study, where they can be cultured, studied in situ, and are amenable to genetic manipulation. They are therefore the best-studied silicifying organism and provide a plentiful source of information on silica biomineralization.

**Figure 7:**
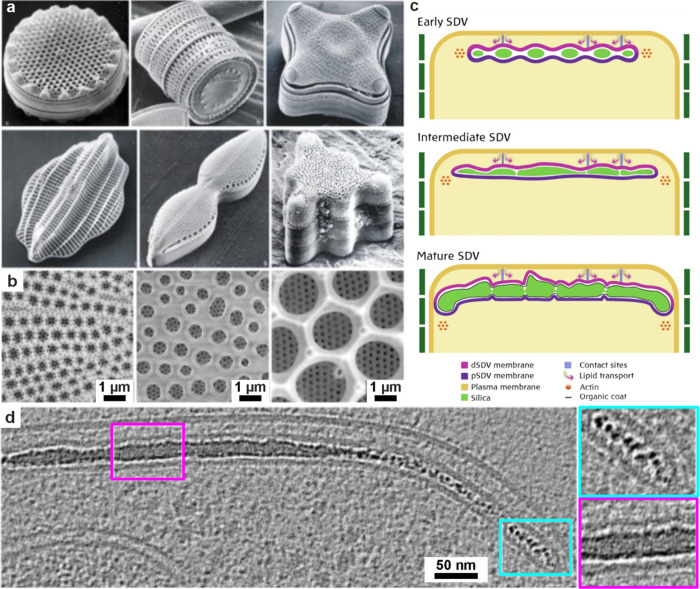
**Morphology of a diatom.** (a, b) SEM images of diatoms. Reproduced with permission from Kröger et al.^80^ Copyright 2007 Elsevier Ltd., all rights reserved. Panels show (a) full diatoms and (b) close-up images of some diatom silica cell walls. (c) Diagram illustrating the development of a diatom silica cell wall within the silica deposition vesicle (SDV) over time. (d) Cryo-ET slice and higher magnification images (colored boxes) showing different textures of silica within the SDV. (c, d) Reproduced with permission from Aram et al.^72^ Copyright 2024 of the Author(s) and Springer Nature with a Creative Commons Attribution CC-BY 4.0 license.

The diatom frustules form within silica deposition vesicles (SDVs), in which the organism can define the pH as well as the concentrations of the silica precursors, biomolecules, and additional ions. This control is critical to regulation of the rate of silica formation. Silicic acid rapidly condenses to silica at concentrations above ≈100 ppm in the absence of molecular stabilizers, where the rate of condensation is pH-dependent and occurs most rapidly at pH 7–8.^286^ The rate is accelerated at higher ionic strengths and in the presence of phosphate, hydroxyl, and fluoride ions, while metal ions retard polymerization.^286,287^ Diatoms maintain acidic conditions of about pH 5.5 within the SDV,^288–290^ which ensures that silicic acid polymerizes slowly^136,291^ unless triggered by local biomineralization motifs. Confining these processes within the SDV gives the diatom precise control over silicification.

Silicic acid membrane transporter proteins are likely involved in concentrating silicic acid within the SDV. These are mounted in the SDV-bounding membrane (the silicalemma) and are used to adjust the internal pH and the concentration of precursors for silica deposition .^292–295^ The silicalemma-associated proteins contain C-terminal sequences that are positively charged and phosphorylated, and these motifs project into the lumen.^296^ These are thought to catalyze the precipitation of silica within the SDV, where molecules with comparable functional groups have been shown to catalyze silica polymerization from silicic acid in bulk solution.^297^ Silicanin-1 from *Thalassiosira pseudonana* is also predicted to localize and organize positively charged long-chain polyamines (LCPAs) within the silicalemma, which may catalyze silica polymerization at specific sites.^290^ LCPAs increase the precipitation of silica in the presence of some soluble proteins, such as silaffins^79^ and silacidins.^298^

Many questions remain about the transport of silicon to the SDV in diatoms.^135,136,299–303^ Nanoscale particles have been observed within diatom cytoplasm, which have been termed silicon transport vesicles (STVs).^304^ These were hypothesized to localize high concentrations of silica precursors at an acidic pH that would retard the polymerization of silicic acid,^288,291^ and deliver them to the SDV for frustule mineralization. However, the low population of these particles suggests that they could not be solely responsible for silicon transport in diatoms,^135,300,305,306^ and molecular inhibitors including aliphatic alcohol-rich polymers^307^ and polyamines^308^ are believed to stabilize high concentrations of silica precursors within the diatom cytoplasm.^299,300^ Recent cryo-ET studies have shown that the silica deposited at early stages in the SDV of the diatom *T. pseudonana* has a granular structure, suggesting that it could form from the assembly of silica nanoparticles.^72^ That the silica is nonuniformly distributed throughout these cells rather than in compartments^302^ further supports hypotheses that the precursor silicon species are stabilized by binding to functional molecules.^303^

Diatom mRNA expression patterns show that genes involved in vesicle trafficking and cytoskeletal transport are upregulated at the same time as biosilicification proteins (e.g., silaffins).^299,309^ This demonstrates that vesicular confinement is important for diatom biosilicification, where it may play roles including the packaging and delivery of organic molecules to the SDV^291,296^ and transporting silica. Observing STVs in cryo-fixed or resin-embedded thin-sections is difficult, so fluorescent dyes and organic molecule labels could be used to follow these processes in vivo. It may be that STVs are transient species that only occur at certain times during silica sequestration (e.g., conditions of high or low environmental silica), silica transport (e.g., high flux), frustule assembly, and frustule growth. Therefore, timing of the observations during the cell cycle may be critical in fully characterizing the transport of silicon in diatoms.

#### 3.6.2 Silica Formation in Sponges

Silicifying sponges form the *Demospongiae* and *Hexactinellida* classes are sessile filter feeders that build hierarchically ordered silica-organic composite structures that protect the organism and anchor it to the sea or lake floor.^310,311^ The deposition of silica spicules is initiated within the axial canal.^312–315^ Silica precursors and catalytic silicification proteins called silicateins are stored within electron-dense and silica-rich vesicles called silicasomes,^316–318^ which are in turn contained within specialized biomineralization cells called sclerocytes. The latter also contain electron poor vesicles^317,318^ that may contain organic components that are also delivered to the site of silicification. The silicasomes are about 100–300 nm in diameter^313,317^ and are present throughout the lifetime of the growing spicule, such that they are much easier to identify than putative STVs in diatoms.

Silicification occurs by fusion of the silicasomes with the axial canal, where silica condensation is facilitated by biomineralization proteins within the confines of a thin deposition area bounded by silicateins.^316–318^ The vesicles enable the sponges to accumulate high concentrations of silica precursors until they reach the silicification zone at the growing spicule. The membrane delimiting the spicule mineralization maintains confinement as the silica polymerizes, helping to define its morphology (see Section 4.1).

## 4 Biomineral Morphologies

There are numerous examples of confinement controlling the shape of biominerals. Morphologies range from the simple, such as the nanosized single crystals of magnetite present in magnetotactic bacteria^319,320^ and some calcite sponge spicules,^35,321,322^ to exquisitely complex structures such as seashells^323^ and sea urchin tests.^324^ Indeed, the morphology of many biominerals is arguably their most distinctive feature.^2,12,325^ Some of the most elaborate are formed by organisms such as diatoms and radiolarians and comprise amorphous silica.^80,326^ With no preferred shape, an amorphous material is an immediate choice to build irregular forms. Complex morphologies can also be readily constructed from small crystalline building blocks, generating polycrystalline biominerals such as the elliptical coccoliths of *Emiliania huxleyi*.^327,328^ However, some of the most remarkable examples of morphological control are where biology shapes large single crystals. While synthetic single crystals exhibit gross morphologies that reflect the symmetry of the underlying crystal lattice, biogenic single crystals can exhibit noncrystallographic forms that bear no relationship to the atomic symmetry of the crystal. The fenestrated form of the calcite single crystal echinoderm skeletal elements offers the perfect example of this category of biomineral.^324^

Soluble additives are extensively used to control crystal morphologies in synthetic systems,^329–333^ and it has been widely postulated that they may also play a key role in the morphogenesis of biominerals, where the soluble macromolecules extracted from within biological crystals can tune crystal morphologies when used as additives in laboratory experiments.^35,333–335^ Modulation of single crystal morphologies can occur by selective binding of the additives to different families of crystal facets,^333,336^ or by stereochemical recognition to distinct step edges on equivalent crystal faces.^337^ This results in simple morphological changes that reflect the symmetry of the underlying crystal lattice. However, it cannot produce single crystal morphologies that break the underlying crystal symmetry, such as the elongated magnetite crystals (that have a cubic lattice) generated by magnetotactic bacteria,^319,338,339^ the curved calcite spicules (trigonal lattice) in the sponge *Sycon*,^35,322^ or morphologically complex single crystals such as the skeletal elements of echinoderms.^56,324,340^

Complex crystal morphologies such as convoluted fibers can also readily form via PILP phases,^182,183,341,342^ although the presence of PILP phases in vivo has yet to be definitively demonstrated.^201^ In certain circumstances, polycrystalline assemblies with extraordinary helical or leaf-like morphologies can form under purely inorganic conditions, such as the coprecipitation of SiO_2_ and BaCO_3_.^343–345^ These so-called “biomorphs” resemble biominerals so closely that they were even taken as microfossils until Garcia-Ruiz et al. synthesized them abiotically.^343^

That biominerals form within confined volumes opens the door to alternative mechanisms of morphological control. At a fundamental level, crystal morphologies can be directed by templating, which in the case of biology is achieved by organic compartments that restrict crystal growth. It is hard to envisage how single crystals with complex morphologies, such as the macroscopic, porous single crystals produced by echinoderms, could be produced by any other route, where the organic compartment can continue to evolve together with the growing biomineral. It has also been postulated that amorphous precursor phases can act in conjunction with templating to control biomineral morphologies, where the absence of a preferred form makes them easy to shape.^139,346^ However, crystals with curved surfaces and complex morphologies comparable to those seen in biology can be generated in synthetic systems by templating routes in both the absence and presence of amorphous precursor phases,^153^ demonstrating that an amorphous precursor is not a prerequisite to morphological control using templating.

Other mechanisms also become possible in confinement. If a crystal occupies an organic compartment such that there is little free volume between the crystal and surrounding membrane, then the introduction of precursor species at specific locations in the compartment could lead to preferential growth in those directions.^69^ The absence of free volume is key to this mechanism, as it ensures that the precursor species are incorporated into the crystal faces local to the inward flux before they can diffuse throughout the entire volume. It has also been postulated that certain proteins can be embedded at specific locations in the membrane, where they can inhibit or promote crystal growth to control shape.^347–349^

Recent studies have also explored the mechanisms underlying the morphogenesis of biominerals including mollusk shells and corals. As compared with traditional ideas that consider that morphogenesis of these biominerals derives from the action of sets of structure-directing macromolecules,^350–352^ these new approaches hypothesize that they can in fact be achieved by regulation of the physicochemical environment in which the mineral forms.^353–355^ The growth and morphological development of the biominerals can then be considered to be the product of the geometric and solution environment, as defined by factors such as the pH, viscosity, and concentration of inorganic and organic precursors. Reducing biomineralization to a physicochemical process in which the organism defines geometric and thermodynamic boundary conditions has enabled phase field theory to be used to successfully describe morphogenesis in a range of systems.^353,355,356^

In considering the effects of confinement on biomineral morphologies, we here provide some contrasting examples and supplement many with descriptions of bioinspired experiments that provide support for the underlying core biogenic strategies. Diatoms are used to illustrate how amorphous biominerals can be shaped to form hierarchically patterned porous structures, while the magnetite crystals generated by magnetotactic bacteria provide excellent examples of small single crystals with simple morphologies. Mollusk shell offers an interesting example of morphological control within a structured, extracellular environment, while the small size of coccolithophores have enabled detailed studies of the pathways leading to the formation of the intricately shaped individual elements that comprise the coccolith scales to be conducted. Finally, we consider echinoderm skeletal elements, where these macroscopic single crystals can exhibit complex porous morphologies resembling periodic minimal surfaces, and the morphological development of the millimeter-scale polycrystalline chiton radular teeth and the stomatopod dactyl club, where these structures assemble within preformed organic templates.

### 4.1 Diatom Frustule Silica

#### 4.1.1 Biological Shape Control in Diatom Frustule Silica

Diatoms construct elaborately patterned siliceous external cell walls (termed frustules) that consist of two valves that fit together like the halves of a Petri dish. These are surrounded by several girdle bands,^357^ and the tops of the valves exhibit a complicated, species-specific pattern comprising well-organized nanopores (Figure 7b).^308^ Each frustule forms within a proteo-lipid bound silica deposition vesicle (SDV) (Figure 7c)^304,357,358^ that is micrometers in diameter but only ≈100 nm thick.^304,359,360^ This constrains silicification to a 2D area, where the SDV partitions this process from the rest of the cell, acts as a locus for molecular organization, and enables regulation of pH and the concentration of silicic acid precursors.

The SDV expands in the *x*–*y* plane, where this has been postulated to occur by merging with 30–40 nm silica transport vesicles (STVs) that contain organic molecules and silica mineral precursors.^304,360^ However, recent cryo-ET studies of the formation of the valves in *T. pseudonana* found no evidence that vesicles merge with the SDV.^72^ Further, the proximity of the plasma membrane and the valve SDV suggests that the lipids and proteins required for growth of the SDV may be transferred from the plasma membrane through contact sites^72^ and that silica precursors may be transported within the cell cytoplasm by complexation with polymers^299,300^ (Section 3.6.1). Silicification then occurs within the SDV in the presence of a range of biomolecules that may be involved in its morphogenesis (Figure 7d).^289,297,298,300,361–363^

Given their beautiful structures, the morphogenesis of diatoms attracts significant interest. The traditional model for the development of porosity is based on phase separation of biomolecules within the SDV, which generate an array of organic droplets that template the silica as it precipitates.^364^ Positively charged LCPAs have been envisaged to phase separate from aqueous solution to form hydrophobic droplets when in contact with anions such as phosphate,^365^ and these droplets then self-assemble into hexagonal arrays within the confines of the SDV.^366^ Silica subsequently precipitates between droplets. This consumes LCPAs as the mineral forms, destabilizing the larger droplet to form a new generation of smaller drops within the silicified pore. These are then mineralized in the same manner, creating fractal patterns.

Recent studies using advanced techniques such as cryo-TEM^72,367^ and proteomic analyses^368^ offer new insight into the morphogenesis of diatom valves and challenge the validity of this simple model. Investigation of the development of the valves of *T. pseudonana* shows that silica ribs branch out from a central circular seed, and that nanoporous silica then forms between the branches, where this first occurs at the valve center (Figure 8a, b).^72,360^ This branching pattern has been reproduced by a minimal mathematical model in which autocatalytic formation of diffusible silica precursors is followed by conversion into solid silica, which releases an inhibitor that retards the conversion of precursors.^369^ This suggests that templating by organic structures is not required to achieve such patterns.^72,360,369^

**Figure 8:**
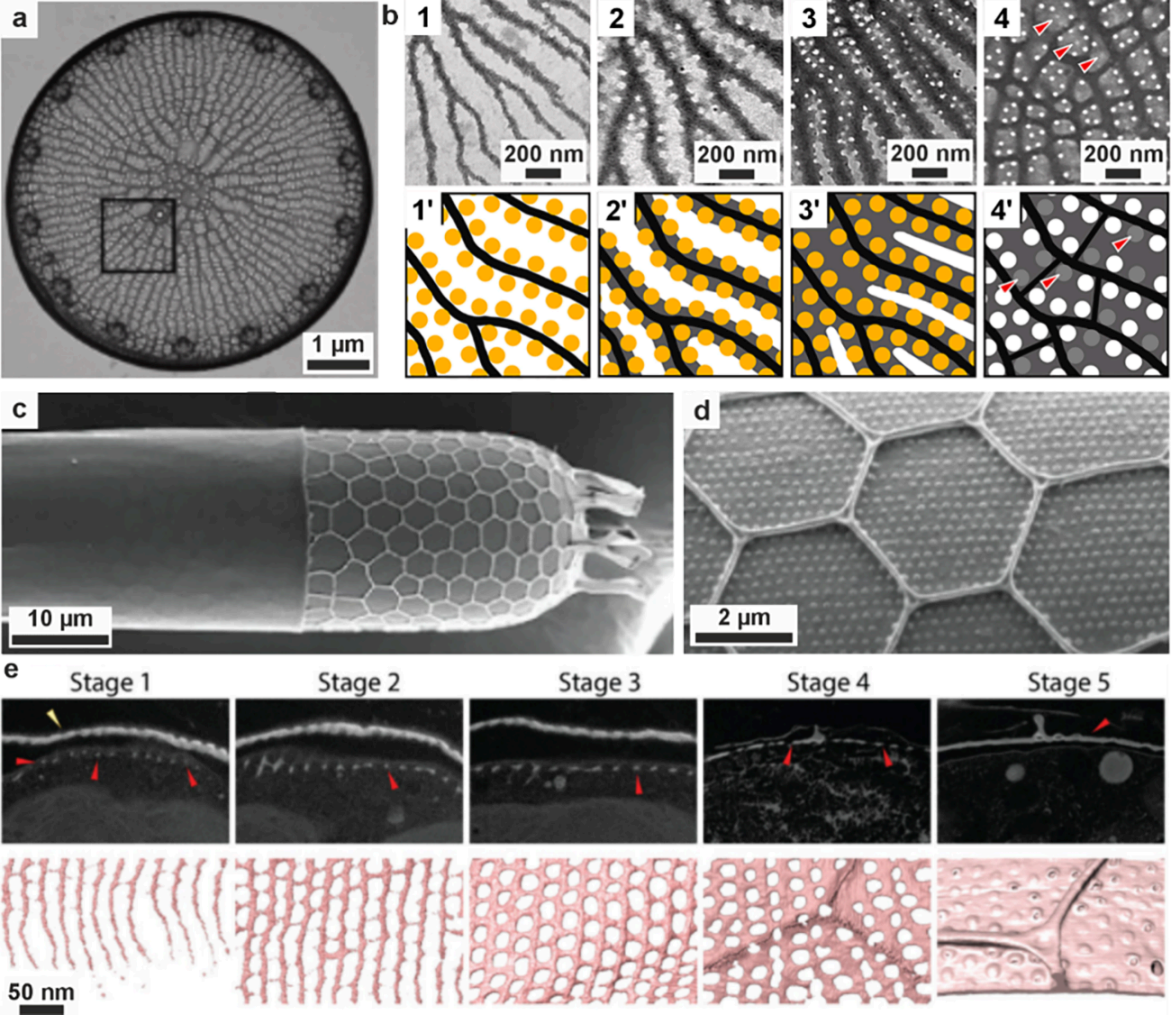
**Development of diatom frustule patterns over time**. (a) TEM image of mature *Thalassiosira pseudonana* and (b) TEM images (top) and schematic diagrams (bottom) showing the development of silica structures of *T. pseudonana* over time, from early (1) to mature stages (4). Reproduced with permission from Heintze et al.^368^ Copyright 2022 National Academy of Science under the PNAS license. In (b), the silica ribs are black, organic nanodroplets are orange, and the cribrum plate silica is gray. The red arrows indicate less electron dense areas that are assumed to form when the unstable cribrum pores partially fill in with silica. (c, d) SEM images of *Stephanopyxis turris* at (c) low and (d) higher magnification. (e) TEM slice and view images of the valve as it mineralizes over time: the top shows slices from resin-embedded samples, and the bottom shows 3D reconstructions of silica in the valve as the pattern develops over time. Adapted with permission from Lansky et al.^367^ Copyright 2024 The Author(s) and Advanced Science published by Wiley-VCH GmbH.

The morphological evolution of the silica valves of *T. pseudonana* has also been studied under near native conditions using cryo-TEM, where the cultures were synchronized before they were harvested.^72^ Cells were initially starved of silicon, causing them to arrest their growth just before cell division. Subsequent addition of silicon then restarts the cell cycle at the same stage, synchronizing the life-stage across the culture. Comparison of the volume of a dense silica phase and dilute residual phase within the SDV showed that silica growth proceeds in the highly confined SDV, and that it appears to be perfectly synchronized with the delivery of lipids and proteins for SDV expansion, possibly through contacts with the neighboring plasma membrane.^72^ Notably, there is a strong correspondence between the positions of nanoscale indentations in the SDV membrane and the pattern of pores in the surface of the silica valve prior to exocytosis, which suggests that the biophysical properties of the SDV (curvature, stiffness, tethering) contribute to the morphogenesis of the silica tests (Figure 8b).^72^ This is further supported by proteomic analysis of the valve SDV, which identified new biomineralization proteins, termed dAnk1–3. These are associated with the cytoplasmic surface of the membrane and may be involved in defining its conformation.^368^

Valve development has also been studied in the diatom *Stephanopyxis turris*, where it was visualized using cryo-ET and TEM of resin-embedded samples (Figure 8c–e).^367^ This revealed that the hexagonal pore pattern arises due to the formation of linking branches between emerging rods rather than templating of a close-packed array of precursor droplets.^367^

It is still hypothesized that phase separation could contribute to the morphological development of diatom silica, however. In *T. pseudonana*, nanopores were seen to initially form at the walls of the branches and then continued to be laid down until they filled the interbranch area.^360^ dAnk1–3 proteins may be involved in this process by influencing the biogenesis of templating nanodroplets,^368^ and it has also been suggested that the pores may be templated by phase-separated nanodroplets or by the formation of patterns of domains within the SDV membrane that direct mineralization.^72,367^ The degree to which a diatom valve is patterned by organic phase separation and/or membrane structuring and anchoring therefore appears to be species specific. However, in all cases, confinement of these processes within the SDV allows the organism to effect precise control on the silicification processes by combining these different physical controls on mineralization.

#### 4.1.2 Learning from Synthetic Silica Systems

Insight into this proposed biogenic templating mechanism for silica has been obtained from in vitro experiments that include the use of polymer additives,^370–374^ proteins,^194^ DNA,^375^ multilamellar vesicles,^376^ and hard templates such as TE membranes.^377^ In common with biology, in vitro synthesis of silica under ambient conditions usually results in amorphous nanoparticles.^378^ Silicification within nanosized, unilamellar phospholipid vesicles generated silica nanoparticles at the membrane surfaces,^378^ whereas 2–3 nm silica nanoparticles formed between the lamellae of onion-like multilamellar phospholipid vesicles, which preserved the original concentric organization of the soft vesicle template.^376^ Nano- to microsized particles were also obtained when silica was precipitated in chitosan and gelatin microemulsion organogels^379^ or reverse micelles.^380^

The combined effects of positively charged additives (to mimic LCPAs) and confinement were investigated by forming silica within the cylindrical pores of TE membranes, which provide a rigid template.^377,381^ Acidified water glass containing poly-l-lysine (PLL) was infiltrated into membranes pores with diameters of 200–1200 nm,^377,381^ and silica nanotubes formed by deposition of nanoparticles on the walls of the nanopores.^377^ Water glass containing PLL gave nanotubes with thicker walls and comprising slightly smaller nanoparticles than water glass alone.^381^ Thinner silica walls also formed in the smaller nanopores (60 nm in 200 nm pores and 150 nm in 1200 nm pores) in the presence of PLL.^381^

Soft templating of silica has also been widely explored, for example, by using positively charged poly­(ethylenimine) to form reverse micelles. Silicic acid was entrapped and condensed within the aqueous cores of the micelles,^380^ and hollow or solid silica spheres could be formed by varying the pH and poly­(ethylenimine) concentration, with the sphere sizes being defined by the dimensions of the confining micelle.^380^ Silica has also been deposited within collagen fibrils to form heavily silicified structures.^382^ Given that the nanopores in collagen are only ≈2 nm in diameter, this shows that the silica precursors can infiltrate into very small pores.^383^ Silicification has also been used to preserve synthetic structures, such as positively charged peptide nanocages,^384^ DNA origami,^375^ and delicate biological soft tissues including embryos and their internal organs.^385–387^ Thin (≈10 nm) coatings are sufficient to preserve the morphologies of soft matter structures, indicating that it is possible to create the intricate shapes of the silica frustules of diatoms by soft-templating.

Methods of generating more complex patterns comparable to those formed in diatoms have also been explored. Recently, the addition of bromide ions to a silica sol thin-film was used to form perforated silica films, where phase separation of water droplets from the sol as it gelled generated a regular array of pores in the silicified film.^388^ Polyamine droplets formed via phase separation have also been used to template silicification^371^ and to generate porous silica structures. The pores in these in vitro synthesized structures were more disordered than those of diatoms, probably because the droplets that templated the silica exhibited a distribution of sizes. As of yet, no attempts have been made to combine the phase separation and silicification within 2D confinement, nor has there been any cycling between the phase separating and silicifying regimes that are thought to generate hierarchical porous structures through sequential phase separation.

Finally, a number of recent studies have explored the role of polymer phase separation in silica precipitation.^372–374^ While pAH and pAA alone both stabilized soluble silicon containing ions, silica precipitation occurred within a dense polymer phase formed when these oppositely charged polymers were combined.^372^ Experiments with positively charged (polyamine) and negatively charged (acrylamide coacrylic acid) polymers showed that mobile silica species concentrate within the dense polymer phases that form by macroscopic phase separation upon mixing, which generates high supersaturations that drive the precipitation of silica.^373^ Both the phase separation and silica polymerization are dependent on the pH, and interactions between the silica species and polymer chains can enable polymerization of a silica–polymer hybrid material in undersaturated conditions.^374^

These in vitro experiments demonstrate that positively charged polymers in combination with soft or hard templates offer an effective way of molding the morphology of silica and add credence to the proposed biosilicification mechanisms. However, more detailed ex vivo studies are required to better understand the control mechanisms in biosilicification. Techniques such as liquid-cell TEM^389^ and micro- and nanofluidics^390^ may allow observation bioinspired silicification processes as they occur in vitro. Excitingly, as liquid-cell TEM provides a 2D confined environment, this may be the perfect vehicle to study phase separation in 2D to better mimic the proposed pattern formation in diatom silica.

### 4.2 Magnetosome Morphologies

#### 4.2.1 Biological Control in Magnetosomes

Biotemplated magnetic nanoparticles aid organisms in navigation by allowing them to sense the Earth’s magnetic field. They have been found in pigeons,^391^ trout,^392^ salmon,^393^ and bees^394,395^ but the best studied are in magnetotactic bacteria (MTB).^396–399^ These bacteria contain chains of oriented magnetic nanoparticles that act like compasses and allow MTB to passively align with an external magnetic field, like the Earth’s magnetosphere. The size and shape of the magnetic nanoparticles vary between different bacterial lineages (Figure 9a, b) but show uniformity within a single species, which indicates that mineralization is under strict biological control.^400–402^

**Figure 9:**
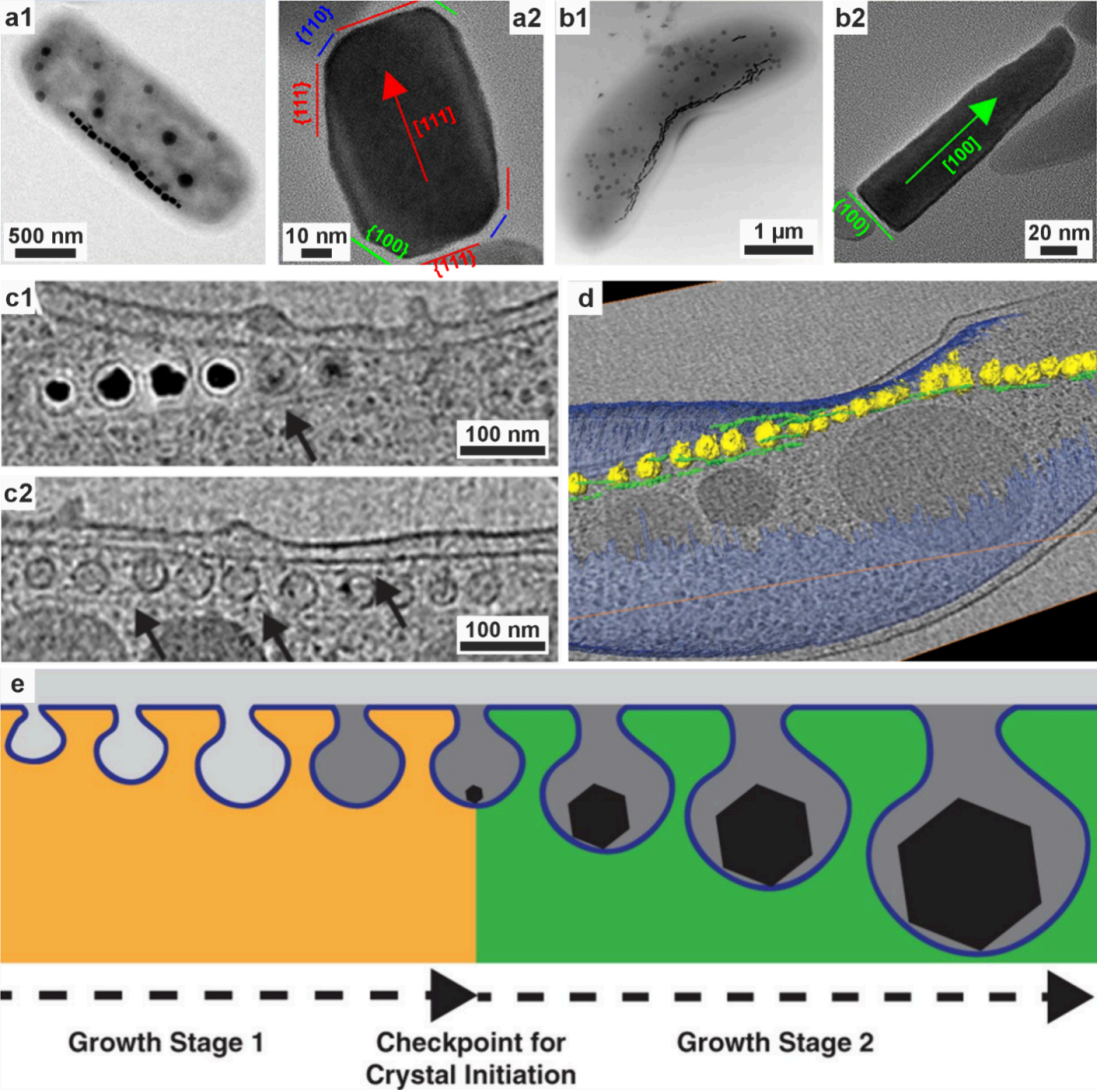
**The morphology and growth of magnetite in the magnetosome.** (a, b) TEM images showing some magnetosome morphologies. (a) *Gammaproteobacteria* strain SHHR-1 produces elongated prisms and (b) *Deltaproteobacteria* strain WYHR-1 synthesizes bullet-shaped magnetite. (a) Adapted with permission from Li et al.^403,404^ Copyright 2017 The Author(s) and the American Society for Microbiology with a Creative Commons Attribution CC-BY 4.0 license. (a, b) Reproduced with permission from Li et al.^28^ Copyright 2020. American Geophysical Union, all rights reserved. (c) Cryo-TEM images of cuboctahedral magnetite from a *Magnetospirillum magneticum* AMB-1 magnetosome chain grown in the (1) presence and (2) absence of iron. This shows that the magnetosome structures form prior to the magnetic particles. (d) Cryo-ET reconstruction showing the 3D organization of magnetosomes (yellow) and their association with the cytoskeletal filament (green). (c-d) Reproduced with permission from Komeili et al.^405^ Copyright 2006 The American Association for the Advancement of Science. (e) Diagram showing a two-step growth mechanism of a magnetosome, where a second growth stage of the vesicles is triggered upon crystal nucleation. Reproduced with permission from Cornejo et al.^406^ Copyright 2016 The Author(s) and The American Society for Microbiology with CC-BY-NC-3.0 license.

Most of our understanding of the formation of the magnetic nanoparticles within MTB is based on a few model organisms.^398^ MTB control the formation of 30–120 nm ferrimagnetic magnetite (Fe_3_O_4_) and/or greigite (Fe_3_S_4_) nanoparticles within proteo-lipid bound organelles called magnetosomes.^407^ Magnetite crystals of this size comprise single magnetic domains,^408–410^ and the magnetic response is further enhanced when they are organized into chains.^400,411,412^ The size, shape, and organization of these magnetic particles are species-specific and include octahedral and cuboctahedral forms in *Alphaproteobacteria*,^347,403,413–419^ elongated prisms in *Gammaproteobacteria* (Figure 9a),^319,420–423^ and bullet (Figure 9b) or tooth-shaped particles in *Deltaproteobacteria* and *Nitrospirae*.^28,78,338,339,424–426^ Individual species of MTB generate magnetite crystals with characteristic morphologies and uniform sizes and shapes,^398,418^ whereas the morphological and orientation control in greigite is less well pronounced.^400,427^ We focus here on the better studied magnetite-producing bacteria.

Depending on the shape of the magnetosome crystal, different mechanisms of morphological control have been proposed. The two best studied MTB (*Magnetospirillum magneticum* AMB-1 and *Magnetospirillum gryphiswaldense* MSR-1)^398^ produce cuboctahedral magnetite crystals within magnetosome vesicles. These morphologies are consistent with the cubic lattice of magnetite, and it has been widely proposed that their morphologies are controlled by proteins such as Mms6,^347,414,428^ MmsF,^348,429,430^ and MamG, F, D, and C^349,431–433^ (see Section 7.4). In vitro experiments with these proteins tend to generate particles that have the same shape as those formed in vivo, although the particle sizes are much smaller. It has therefore been suggested that the size of the particles may be directed by the magnetosome.

Empty magnetosome vesicles form prior to mineralization in both *Magnetospirillum* species (Figure 9c1).^405,434–436^ Initially, the unmineralized compartments form from invagination of the internal cell membrane.^405^ They are then remodeled and enlarge as the mineral core grows (illustrated in Figure 9e). Cornejo et al.^406^ used *Δmmsf* knockout mutants of AMB-1 to show that the magnetosome membranes form and then grow to their final size after mineral nucleation, even when the crystal growth is arrested by loss of the MmsF protein. The *Δmmsf* phenotype magnetosome membranes are the same size as those seen in the wild type, but the magnetic mineral cores are significantly smaller. The expansion of the magnetosome vesicle is therefore not due to the growing mineral applying pressure or stretching the membrane.^406^ As neither the proteins nor magnetosome chamber alone can form crystals that resemble the wild-type particles in size and shape, it is clear that they act in combination to generate cuboctahedral magnetite particles in vivo.

Looking then at magnetite crystals with noncubic symmetries, some MTB generate prismatic particles that are elongated parallel to the ⟨111⟩ axis.^319,437–441^ This morphology maximizes the magnetic moment of the nanoparticle^440^ and thus that of the assembled magnetosome chain. Magnetosome vesicles again form prior to mineralization and have elongated shapes that have the aspect ratio of the final crystal.^422^ The empty vesicles are about 70% of the size of the mature crystals, which suggests that the organelle grows as the crystal matures,^422^ possibly in a similar way to that demonstrated for AMB-1.^406^

In bullet shaped magnetosomes, the magnetization alignment is along a ⟨100⟩ magnetic hard axis.^424^ Although vesicles with similar sizes and shapes have been detected in bacteria prior to the formation of crystals,^426^ crystals have not been directly observed within these structures. Instead, amorphous iron phosphate particles are initially observed within the vesicles, and may act as ion reservoirs for mineralization.^426^ Initially, bullet-shaped magnetosomes do grow isotropically, with anisotropic growth occurring later as the bullet shape is formed.^425^ No organic membrane has been observed around the mature crystals by TEM, which suggests that the bullet-shaped particles are only transiently confined within the vesicles and are released as they mature. Alternatively, the magnetosome may confine the particles so closely that it cannot be differentiated from the particle surface by TEM.^426^ It is possible that the transition from isotropic growth to anisotropic elongation^425^ is controlled by a change in confinement, with this transition from the early stage vesicular confinement to the condition where a biological membrane is no longer visible triggering the symmetry breaking in bullet magnetosomes. Notably, no in vitro study using biotemplating proteins has succeeded in producing the bullet morphology. This emphasizes the importance of confinement templating effects and/or magnetosomes acting as a locus for biomineral templating biomolecule organization in controlling magnetosome crystal morphologies.

#### 4.2.2 Learning from Synthetic Magnetic Mineral Systems

Ex vivo synthesis of magnetite nanoparticles with comparable sizes and shapes to those formed in magnetotactic bacteria has proven challenging. First, synthetic crystals produced in aqueous solution under ambient conditions are typically significantly smaller and more polydisperse in size and shape than those from MTB.^442^ Heat is usually required to synthesize larger monodisperse crystals abiotically (e.g., Figure 10a),^442^ although these are smaller than magnetosomes. Magnetite crystals formed in the presence of Mms6, an acidic protein extracted from MTB, were smaller (20.2 ± 4.0 nm) yet morphologically similar to the cuboctahedral crystals seen in the AMB-1 parent organism (Figure 10b).^347,428^ In contrast, the positively charged electrolyte poly-l-arginine induced the formation of 35 ± 5 nm roughly spherical magnetite crystals that exceed the superparamagnetic threshold (25 nm) such that they are ferrimagnetic and tend to align in chains (Figure 10c).^443,444^ Similar effects were found for MamC^431^ and synthetic copolypeptides.^445^

**Figure 10:**
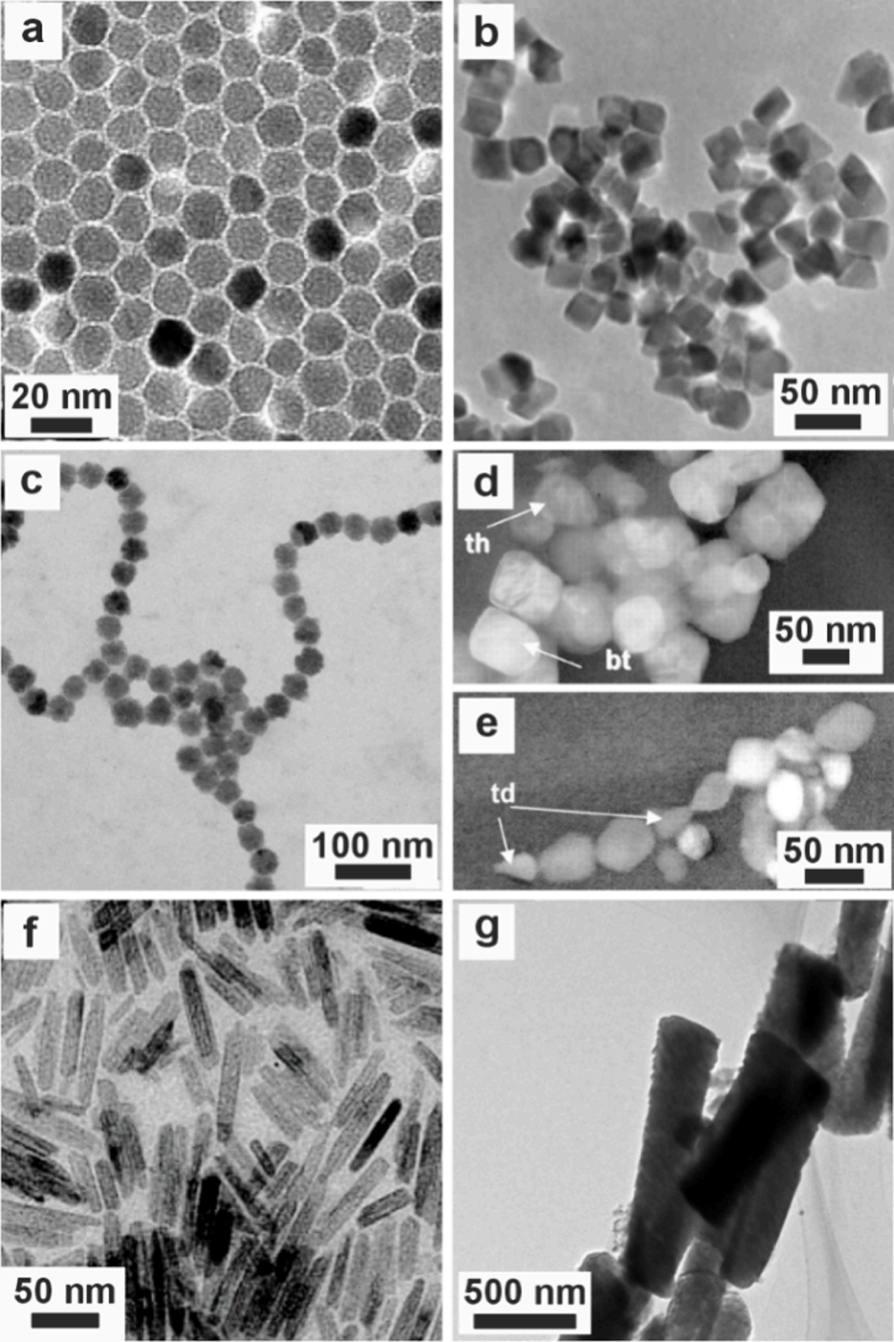
**Magnetite nanocrystals synthesized in vitro.** (a–c) TEM images of monodisperse equidimensional magnetite crystals synthesized by (a) heating iron­(III) acetylacetonate in the presence of oleic acid at 300 °C, (b) adding Mms6 at 90 °C, (c) and adding poly-l-arginine at 25 °C. (a) Reproduced with permission from Sun et al.^442^ Copyright 2004 American Chemical Society. (b) Reproduced with permission from Amemiya et al.^428^ Copyright 2007 Elsevier Ltd., all rights reserved. (c) Reproduced with permission from Baumgartner et al.^443^ Copyright 2014 American Chemical Society. (d–g) TEM images of elongated magnetite crystals synthesized by (d, e) heating Fe-rich carbonate to 470 °C at low pressures, (f) reducing FeOOH at 200 °C, and (g) electrochemical deposition within TE membranes at 65 °C. (d–e) Reproduced with permission from Golden et al.^446^ Copyright 2001 American Mineralogist and The Mineralogical Society of America. (f) Reproduced with permission from Mohapatra et al.^447^ Copyright 2015 The Royal Society of Chemistry with CC-BY-3.0 license. (g) Reproduced with permission from Zhang et al.^448^ Copyright 2017 Elsevier Ltd. and Techna Group S.r.l, all rights reserved.

The significant challenge of controlling the shape of magnetite crystals abiotically led to the elongated magnetite crystals found in the Mars meteorite ALH84001 in 1996 being considered as evidence for extra-terrestrial life.^449^ However, the subsequent finding that elongated magnetite can be synthesized by heating Fe-rich carbonate at high temperatures (Figure 10d-e)^446^ shows that this morphology cannot be reliably used as a biosignature. Magnetite nanorods can also be synthesized using methods such as the thermal reduction of FeOOH nanorods (Figure 10f),^447^ or hydrothermal reduction of FeCl_3_ using N_2_H_4_,^450^ although these are morphologically different to MTB magnetite. Notably, elongated magnetite nanorods have been synthesized by the electrochemical deposition of magnetite within the pores of TE membranes, although there was no preferential crystallographic axis of elongation (Figure 10g).^403^ Precipitation of magnetite within the confines of self-assembled surfactant systems, such as water-in-oil emulsions typically generates very small (<10 nm) nanoparticles.^451,452^

These results strongly suggest that the nanoscale dimensions of the magnetosome nanoreactor and the confinement that this creates are key to controlling magnetite particle size and shape. This highlights how organisms can harness confined volumes to generate crystal morphologies that cannot be produced from bulk solution. Both the nucleation and growth of the crystals can be carefully controlled within the magnetosome, where the organism can change the composition of the solution (including the presence of organic additives) over time, such that the crystal can grow until the magnetosome is filled. Elongation of the crystals may be achieved by directional flow of ions into the magnetosome, and/or anisotropic interactions of the magnetosome membrane with the growing crystals.^453,454^

### 4.3 Morphological Control in Calcium Carbonate

#### 4.3.1 Biological Shape Control in Mollusk Shells

Mollusk shell has long been one of the primary model systems for biomineralization studies, such that its structure, composition and formation mechanisms have been investigated extensively.^455–457^ Mollusks include bivalves, gastropods, and cephalopods, all of which produce calcium carbonate shells that are composites of calcite and/or aragonite and organic macromolecules. Their shells vary microstructurally, but in all cases the mineral is deposited in defined layers that have been structurally classified as columnar prismatic, fibrous prismatic, nacre, crossed lamellar, foliated, and granular (Figure 11).^455,456,458^ The size, shape, mineral type, and organization of the constituent blocks of these layers vary between these structural types, but they all show a high degree of morphological and orientational ordering (the orientation of nacre is discussed in Section 5.1.1).

**Figure 11:**
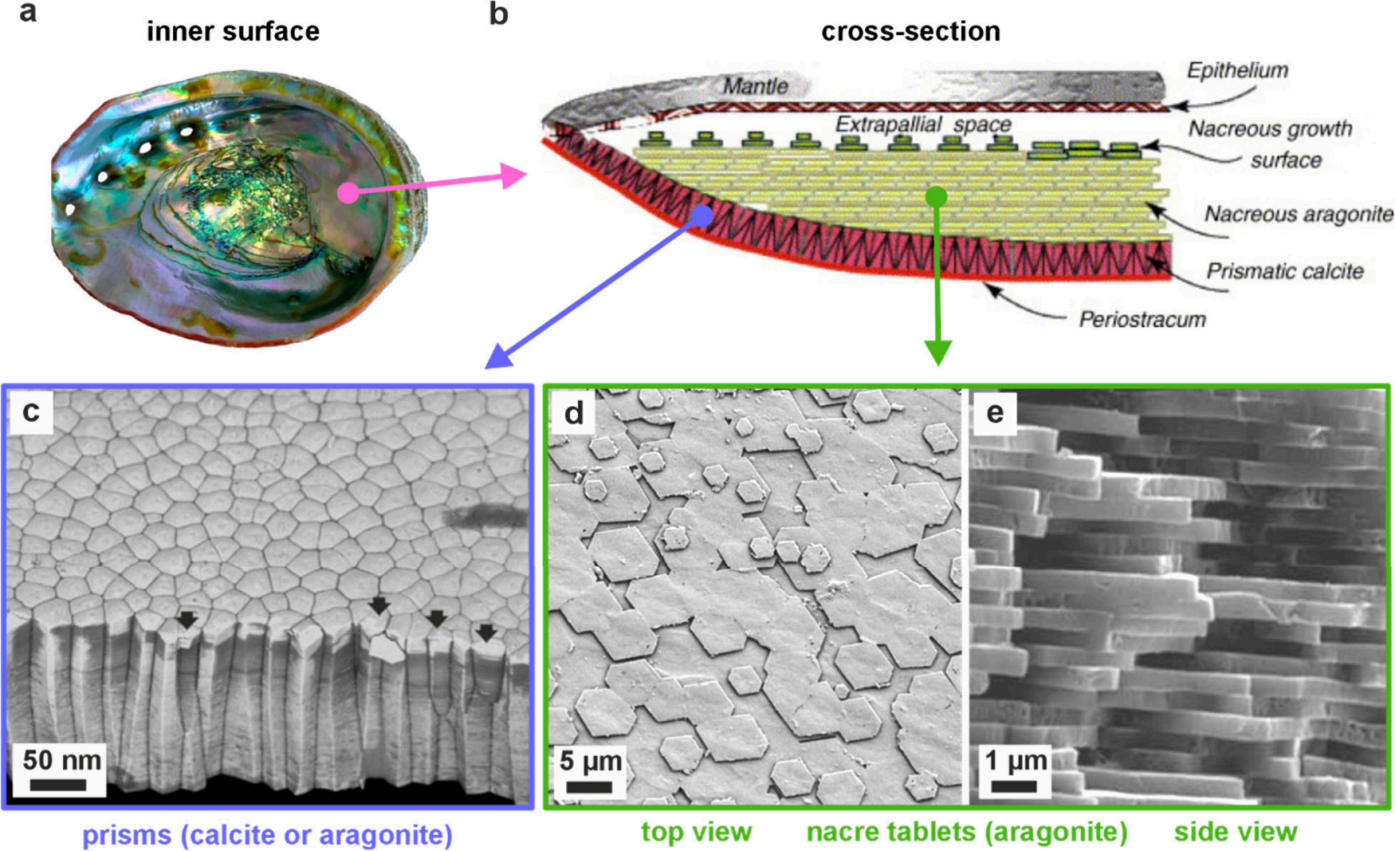
**Morphology of nacre.** (a) Optical image showing the iridescence of red abalone (*Haliotis rufescens*) nacre. Reproduced with permission from DeVol et al.^459^ Copyright 2015 American Chemical Society. (b) Diagrammatic cross-section of the structure of a mollusk shell. Reproduced with permission from Lin & Mayers^460^ Copyright 2004 Elsevier B.V., all rights reserved. (c) SEM image of the prismatic calcite layer of *Pinctada margaritifera*. Reproduced with permission from Checa^455^ in Frontiers in Marine Science. Copyright 2018 The Author(s) with a Creative Commons Attribution CC-BY license. Arrows denote prisms with fewer than six sides, which disappear during growth (bottom of the image). (d) Top and (e) side view SEM images of bivalve *Atrina rigida* nacre. Reproduced with permission from Nudelman et al.^43^ Copyright 2005 Elsevier Inc., all rights reserved.

Nacre and prismatic columnar structures are particularly well-described. Nacre, or mother-of-pearl, appears as an iridescent layer on the inside of many mollusk shells (Figure 11a).^461,462^ The iridescence arises from light scattering by the nacre, which comprises layers of close-packed aragonite tablets that are ≈300–500 nm thick, 5–20 μm wide, and separated by organic sheets.^463–465^ In bivalves, the aragonite tablets are organized in a brick-and-mortar structure, while in gastropods and cephalopods they are stacked on top of one another in towers.^456^ Given that bulk aragonite forms as elongated prismatic or acicular crystals, often aggregated in wheatsheaf morphologies, organisms clearly exert exceptional control to create aragonite crystals with the plate-like forms observed in nacre.^12^ Shells can also contain prismatic columnar structures that can be either calcite or aragonite and extend perpendicular to the growth surface.^466^ The widths of the prisms can vary considerably between organisms^467^ and are again separated by organic matrices.

Despite their differences in appearance, these mollusk shell structures share common formation mechanisms.^455,457^ Shell formation is initiated by the mantle, which secretes a thin organic layer termed the periostracum. This creates an isolated extracellular environment between the mantle and mineral growth surface called the extrapallial space and provides the surface on which new mineral deposits (Figure 11b).^460^ Mineral formation then continues as the epithelial cells of the mantle deliver organic and inorganic precursors. This moves the mineralization front away from the periostracum as the extrapallial space moves up with the mantle. The intervening space is filled with prisms adjacent to the periostracum (Figure 11c),^455^ and the nacreous aragonite (Figure 11d)^43^ appears above this adjacent to the extrapallial space. All crystals therefore grow in competition with each other within a confined space.

The morphological development of mollusk shells has attracted considerable attention. Considering first calcitic columnar prismatic microstructures, the mature prisms of a number of pterioid and ostreoid bivalves are surrounded by 0.5–3 μm thick organic membranes.^466,468,469^ There is some controversy about the role of these membranes in determining the prism morphology, where it is noted that nonmineralized cavities are sometimes observed.^455^ One suggestion is that the system initially comprises two-phases: a fluid, gel-like organic precursor and a PILP-phase mineral precursor. The organic phase self-organizes to generate a morphological template that defines the mineral shape,^455^ and cellular activity then facilitates mineralization and crystal growth. The mantle cells in contact with the growing mineral may uniformly secrete mineral and organic components that diffuse across the extrapallial space, or organic and mineral precursors are selectively directed to specific locations.^466^ Recent work has also proposed that some of the organic material is expelled from the PILP phase as it crystallizes.^470^

There has been much discussion about the relative importance of biological and physical control over the formation of mollusk shells, leading to the development of physical models for their morphogenesis.^353,354,356,455,457,471^ Investigation of the evolution of the calcitic prismatic layer of the bivalve *Pinna nobilis* using synchrotron X-ray microtomography shows that the average cross-sectional area of the individual prisms increases with time, while the total area of the interfaces between the prisms decreases. The prisms are organized such that three prisms meet at each vertex, subtending angles of approximately 120 degrees.^457^ This morphological evolution is comparable to the classical grain growth and coarsening observed in many polycrystalline materials, which is driven by a reduction in the interfacial area between the prisms.^457^

The morphology of nacre has also been attributed to simple competitive growth effects. In nacre, aragonite tablets nucleate and grow within a pre-existing layered extra-cellular matrix (ECM)^472^ that consists of β-chitin that self-organizes into crystalline lamellae^34,456^ and interlamellar spaces filled with a silk fibroin gel that creates a hydrophobic microenvironment (Figure 12a).^43,473^ The height of the gel-filled interlamellar space is commensurate with the final thickness of the aragonite tablets, suggesting that it defines the thickness of the platelets.^472^ The nacre tablets grow normal and parallel to the organic interlamellar sheets (Figure 12b),^456,474^ and lateral growth generates tessellated polygons that resemble a Voronoi diagram (Figure 12c).^475^ This is constructed by scattering a set of points on a plane and then drawing cells such that their contents are closest to the enclosed point. Correlating this with the mechanism of formation of the nacre, each aragonite tablet nucleates at a single point and grows at the same rate in all directions such that it acquires a cylindrical shape. With further growth, the proto-tablets come into contact, causing them to become polygons (Figure 12c2) with shapes that are determined by the distribution of the nucleation sites.^475^

**Figure 12:**
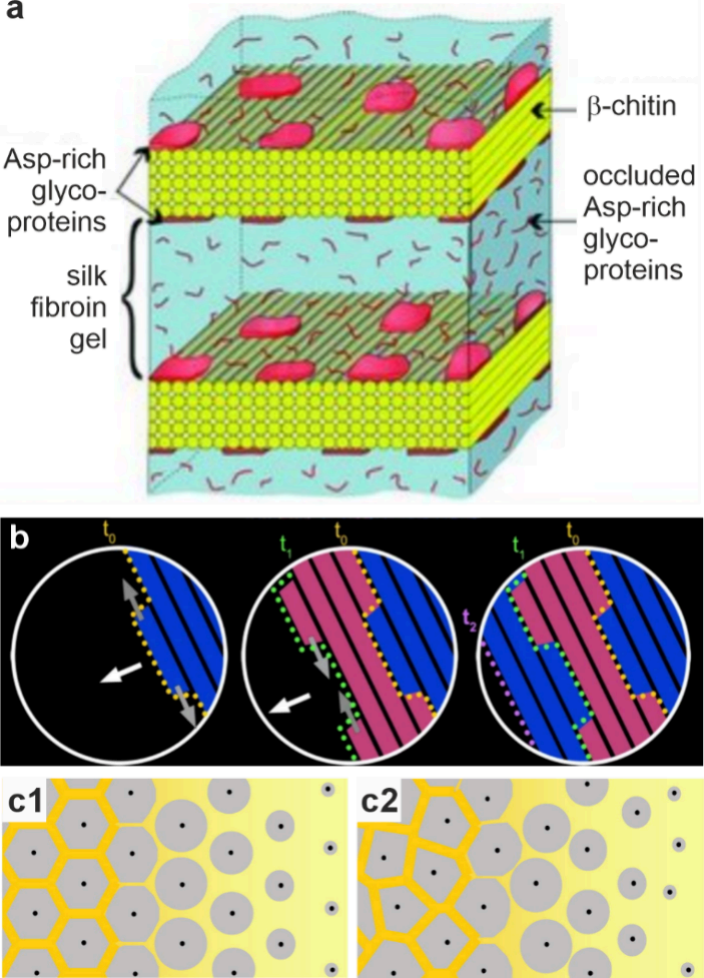
**Effect of confinement on the nacre morphology.** (a) Diagram of the organic layers in nacre. Reproduced with permission from Asenath-Smith et al.^476^ Copyright 2012 WILEY-VCH Verlag GmbH & Co. KGaA, Weinheim. Adapted with permission from Levi-Kalisman et al.^473^ Copyright 2001 Academic Press, all rights reserved. (b) Schematic diagram illustrating the development of nacre at three time points: *t*_0_ (blue), *t*_1_ (blue-pink), and *t*_2_ (blue-pink-blue), based on Sr-labeling experiments.^474^ The scheme shows nacre growth perpendicular to the organic interlamellar sheets (white arrows), followed lateral space-filling nacre growth of tablets parallel to the interlamellar sheet within nacre layers (gray arrows). Reproduced with permission from Otter et al.^474^ Copyright 2023 of the Author(s) and Springer Nature with a Creative Commons Attribution CC-BY 4.0 license. (c1) A Voronoi model showing the idealized formation of hexagonal plates in a 2D confined space and (c2) a more realistic model showing the formation of polygonal shaped plates. Reproduced with permission from Rousseau et al.^475^ Copyright 2004 Elsevier Inc., all rights reserved.

Finally, phase field models have been successfully used to model biomineralization in mollusks and corals.^353,355,356,477^ These phenomenological models are widely used in materials science to describe crystallization on the mesoscale under set boundary conditions^478,479^ and can therefore be used to explore the physical parameters that govern biomineralization in extracellular environments. Models were created to describe the formation of mollusk shell ultrastructures in 2D and were able to predict the change of mineral morphology from granular to prismatic to particle that is seen in many species. Analysis of time scales was also consistent with crystallization from an amorphous precursor phase,^356^ as has been suggested in biological studies (see Section 3).

These models were then extended to identify the key parameters responsible for the growth kinetics and morphogenesis of mollusk shell.^464^ Columnar structures that resemble the prismatic layer form due to competitive growth effects, and nacre-like morphologies are created by (i) secretion of an organic membrane ahead of the growth front, (ii) filling this confined volume with a gel, and then (iii) subsequently replacing the gel with crystalline calcium carbonate.^464^ This may be facilitated by careful positioning of calcium binding acidic proteins to help increase local calcium concentrations prior to mineral formation.^480,481^ Pores in the membranes connect adjacent chambers, and the mineral grows through these pores to generate a new tablet with the same crystallographic orientation as its pore connected neighbor.^464,474^ The separation, the position of the pores and the contact angle of the membrane with the crystal all have a significant effect on controlling the development of nacre. These studies give insight into the physical parameters that control the mineralization of mollusk shell and highlight the role of competitive crystal growth within a confined compartment.

#### 4.3.2 Coccolith Morphologies

Coccolithophores are marine algae that are covered by 3–30 μm diameter calcitic plates (coccoliths) that form an exoskeleton called the coccosphere.^3,482^ The coccosphere protects the algae against predation^483^ and infection by viruses and bacteria^484^ and may also enhance photosynthesis,^485^ reduce damage from strong light^486^ and contribute to buoyancy control.^487^ Coccoliths exhibit a wide range of species-specific morphologies,^3,482^ where most are elliptical plates constructed from elaborately shaped calcite units.^327,482^ The sophisticated shapes of these calcite single crystals provide excellent examples of complex crystal morphologies that bear little resemblance to the rhombohedral form of synthetic or geological calcite.

Coccolith formation has been studied in a range of species, giving insight into the biogenic strategies used to control the morphologies of the constituent crystals. Mineralization occurs within an intracellular compartment termed the coccolith vesicle (CV)^47,488^ and begins with the generation of a proto-coccolith ring on an organic baseplate formed from cellulose fibers, insoluble proteins, and other polysaccharides.^46,47,151,489,490^ The proto-coccolith ring comprises a circle of rhombohedral calcite crystals located at the rim of the baseplate that have alternating orientations.^491^ Of these, the V-units are oriented with the crystallographic *c*-axis perpendicular to the baseplate, whereas the R-units have the *c*-axis radial to the baseplate. These crystals then grow along specific directions within the coccolith vesicle until they become mechanically interlocked in the fully developed coccolith. The relative sizes of the V- and R-units and their individual morphologies vary according to the species.

A detailed study of the evolution of crystal morphologies in *Pleurochrysis carterae* used cryo-ET to determine how the crystals develop their characteristic anvil–like shapes (Figure 13a)^492^ and proposed that the organization of the crystals plays an important role in defining the morphologies. Competition for space between neighboring crystals limits possible growth directions, and together with the direction of preferred growth of the oriented crystals, acts in tandem to inform their morphological development. Notably, the anvil shape only forms correctly if the V- and R-units are adjacent, as their orthogonal orientations are such that they overgrow each other when they come into contact (Figure 13b). If a defect occurs where adjacent units are V/V or R/R, then cuboid-like crystals develop (Figure 13c). This supports an earlier similar competitive growth model for *P. carterae* that was proposed based on SEM and electron backscattering diffraction results.^493^

**Figure 13:**
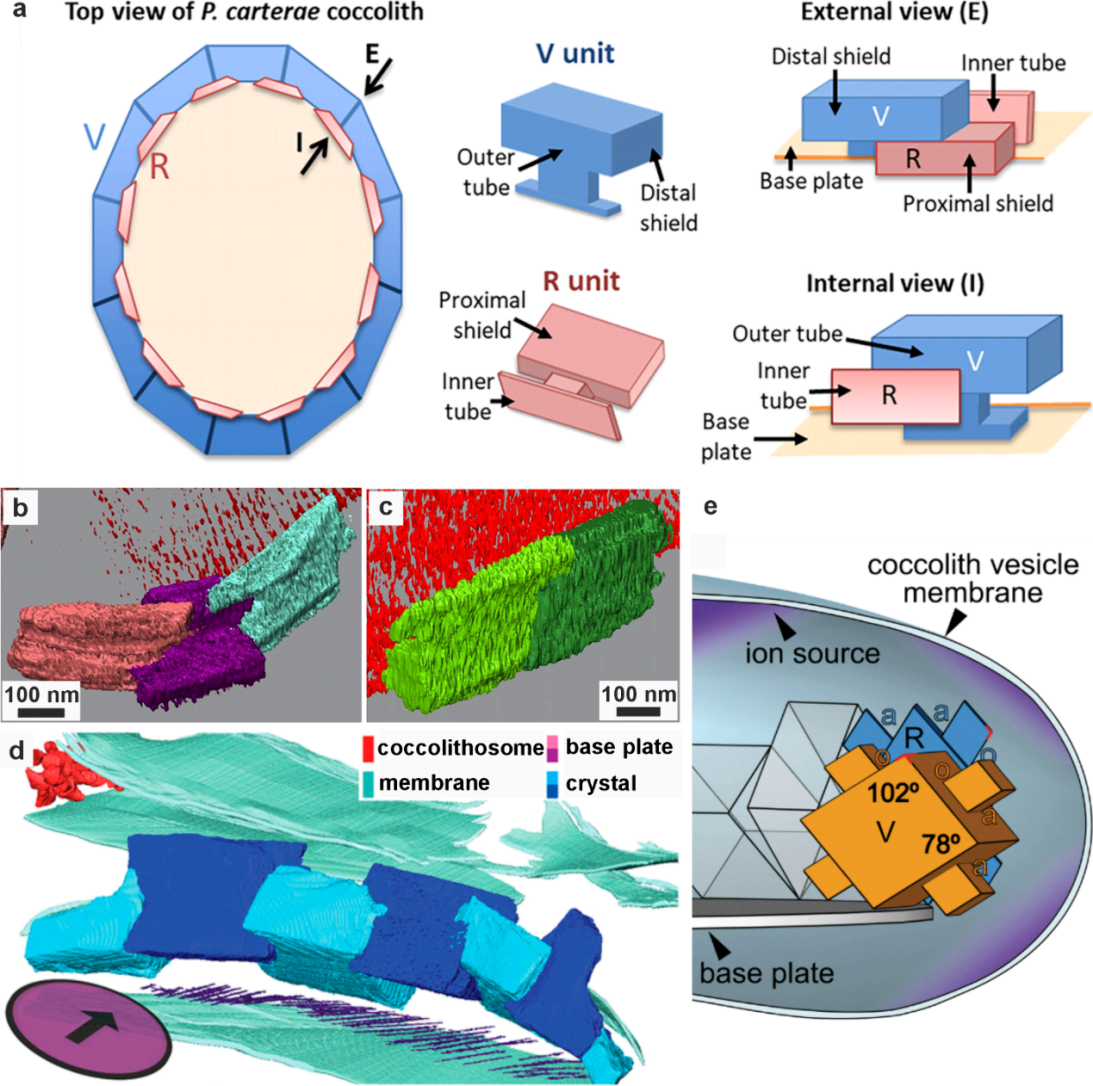
**Role of confinement in controlling coccolith morphology.** (a) Diagram showing the morphology of the coccoliths in *Pleurochrysis carterae*. Cryo-ET reconstructions of (b) adjacent V-R-V units and (c) two adjacent V-units in *P. carterae* coccoliths. (a–c) Reproduced with permission from Walker et al.^492^ Copyright 2020 Elsevier Inc., all rights reserved. (d) 3D surface rendering of *P. carterae*. The purple ellipse illustrates the viewing angle relative to the base plate. Reproduced with permission from Kadan et al.^151^ Copyright 2021 National Academy of Science under the PNAS license. (e) Scheme showing the anisotropic ion concentration gradients (purple) in the confined environment of the coccolith vesicle. The {104} facets of calcite grow at different rates depending on their orientation with respect to the ion flux. As the V- and R-units have different growth step orientations, they grow at different rates under the same ion flux and so show different preferential growth directions. Acute steps are labeled *a* and obtuse steps are labeled *o*. Reproduced with permission from Avrahami et al.^70^ Copyright 2022 The American Association for the Advancement of Science.

An investigation into the development of *Calcidiscus leptoporu*s using electron tomography gave further insight into the role of the coccolith vesicle in the morphological development of individual units.^70^ The mature V- and R-units are shaped as double-shields that express only {104} facets, and their asymmetric morphology was attributed to growth occurring within the confining coccolith vesicle. This membrane is separated from the enclosed crystal by just tens of nanometers in many species,^151^ which may contribute to the generation of smooth, curved mineral faces. The confinement provided by the CV may allow the organism to control where precursor species are delivered to the growing crystal, creating anisotropic growth and breaking the symmetry of the crystal (Figure 13e).^70^

Notably, the morphologies of the coccoliths in *P. carterae* and *C. leptoporus* are quite simple when compared to those in species such as *Emiliania huxleyi*, where the latter are constructed from individual calcite crystals with hammerhead morphologies.^328,494^ The growth of the V-units in *E. huxleyi* is limited by the R units, such that the mature coccoliths comprise elaborately shaped R-units interdigitated with small V units.^494^ A recent study used STEM tomography and cryo-ET to investigate the morphogenesis of coccoliths produced by *E. huxleyi*.^69^ This showed that crystal growth was anisotropic, that crystallographic facets are present at the early stages of growth, and that noncrystallographic curved surfaces develop as elongation continues. Crystal growth alternates between different primary crystallographic directions as the crystals develop, such that their gross morphologies are governed by the crystallographic structure of calcite.^69^ Finally, a recent 3D imaging study of the morphological evolution of *Gephyrocapsa oceanica* again suggested that the confined environment in which the calcite crystals grow plays a key role in determining their morphologies, where the crystals grow in competition with each other.^495^

Cryo-ET of sections cut through *E. huxleyi* gave further information about the proximity of the coccolith vesicle membrane to the developing crystals.^69^ This showed that they are separated by 40–80 nm in the initial stages of growth, when the developing crystals principally display well-defined crystallographic planes.^69^ This separation reduces to under 20 nm at the curved surfaces, while separations of 40 nm are maintained when the elements only elongate at their apexes during stem formation and shield expansion.^69^ This suggests that rapid growth of the crystals is correlated with their greater separation from the enclosing membrane, which in turn allows the required supersaturation conditions to be established to enable crystal growth.

Morphogenesis in *E. huxleyi* was therefore suggested to be the product of a balance between reaction-limited and transport-limited growth regimes, and spatial and temporal control over the transport of ions into the coccolith vesicle. The absence of membranes between neighboring shields suggests that the gaps between them are controlled by the crystal growth regime. The elongation of the distal shield elements in turn is defined by the delivery of their constituent ions, where this occurs at specific sites in the coccolith vesicle.^69^ The coccolith vesicle expands as the crystal grows, as is also observed in magnetotactic bacteria,^406^ and crystal growth ceases when the supply of ions terminates. This is marked by the close proximity of the membrane to the crystal.

While further experiments are required to build a complete understanding of how coccolithophores control crystal morphologies, the relative simplicity and small size of these organisms make them well-suited to this task. Identifying key structural and biomineralization proteins in coccolithophores would advance their study, as this would allow genetic manipulation to label key proteins of interest. This could then be correlated with the excellent cryo-ET studies to show structure–function relationships in their biomineralization at the nanoscale.

#### 4.3.3 Echinoderm Morphological Control

Sea urchin larvae are historically one of the best model systems for investigating biomineral morphogenesis, since they can be readily studied using optical microscopy. The morphologies of the nascent inorganic spicules and their surrounding organic environments can therefore be studied as they develop within the living organism (Figure 14a). The first-formed larval spicules adopt a triradiate form and are shaped by the compartment in which they grow (Figure 14d).^496^ Observations of echinoderm larvae have shown that spicule formation is preceded by the assembly of primary mesenchyme cells (PMCs). These fuse to form syncytial masses with triradiate morphologies and are connected to neighboring cell assemblies by pseudopodal strands (Figure 14c).^497,498^ Mineralization begins with the formation of a faceted single crystal of calcite within the syncytium (Figure 14e) that is oriented such that the *a*-axes of the crystal are aligned with the pseudopodal strands (Figure 14f). Growth then continues by the development of rod-shaped outgrowths to generate a triradiate spicule.^133^ The crystal remains within the confines of the syncytium throughout its growth, and the shape of the compartment defines the morphology of the spicule (Figure 14d).^207,499^

**Figure 14:**
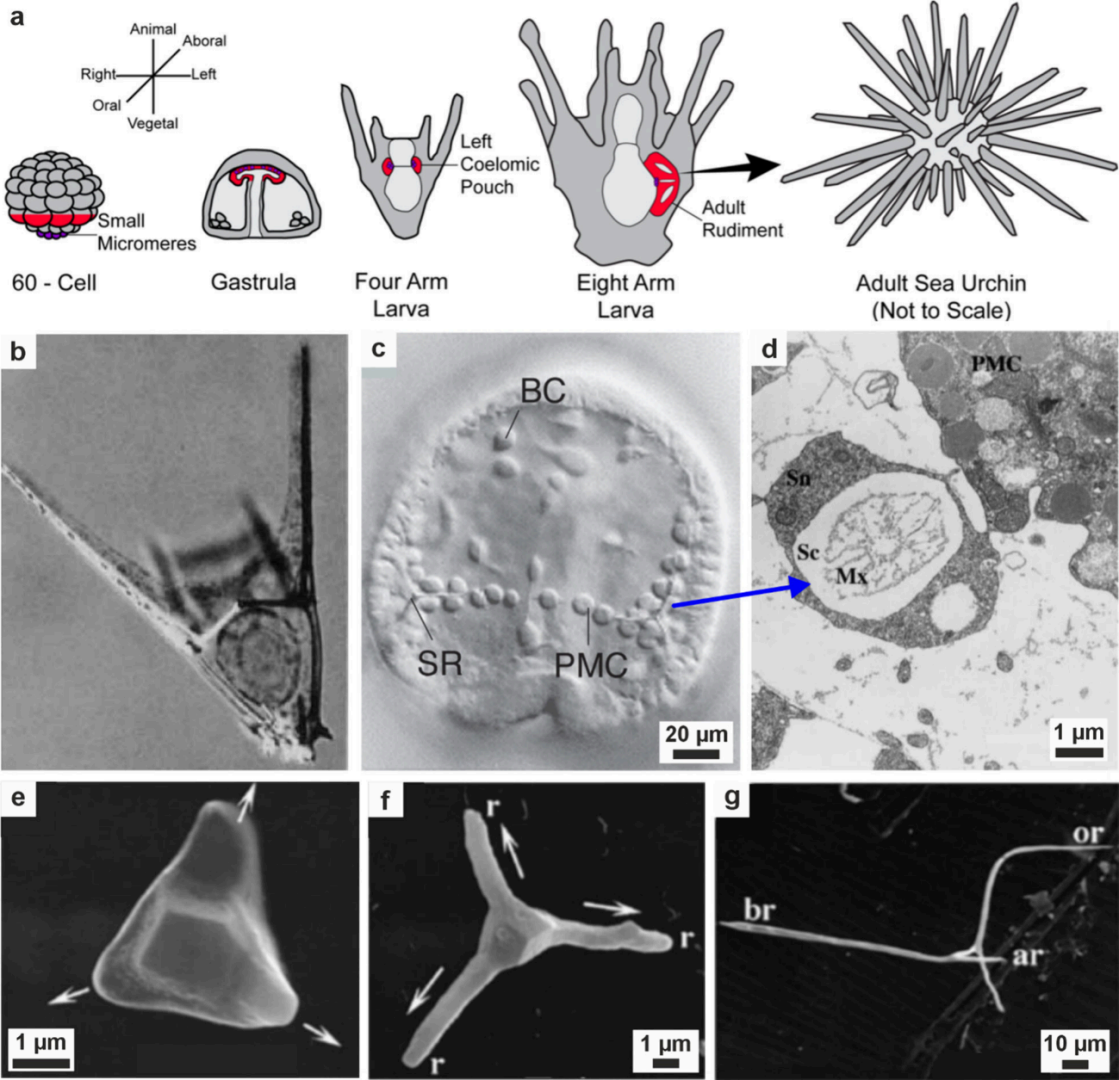
**Sea urchin larval spicule formation.** (a) Scheme showing development of a sea urchin larval spicule. Reproduced with permission from Warner et al.^500^ Copyright 2012 The Author(s) and The Public Library of Science with a Creative Commons Attribution license. Small micromere (blue) gamete cells migrate to the nonskeletogenic mesoderm (red) as the organism differentiates, which is important for determining symmetry as the sea urchin develops. (b) Optical microscope image of the 4-arm larval stage of *Arbacia punctulata* under polarized light. One spicule (right) is at extinction, whereas the other (left) appears bright, showing individual spicules are single crystals. Reproduced with permission from Okazaki and Inuoé^496^ Copyright 2008 John Wiley and Sons. (c) A differential interference contrasts optical micrograph showing the formation of calcitic spicules in a syncytium within a *Lytechinus variegatus* embryo. Labels: blastocoel cell, *BC*; primary mesenchyme cell, *PMC*; and skeletal rudiment, *SR*. Reproduced with permission from Ettensohn^501^ Copyright 1990 Published by Elsevier Inc. (d) TEM image showing the cross section of the spiculogenic envelope from the sea urchin *Paracentrotus lividus*. Reproduced with permission from Beniash et al.^207^ Copyright 1999 Academic Press, all rights reserved. The spicule mineral partially dissolved during preparation. Labels: ectoderm, *EC*; organic matrix, *Mx*; primary mesenchyme cell, *PMC*; spiculogenic compartment, *Sc*; and syncytial envelope, *Sn* of the spicule. (e–g) SEM images of the sea urchin larval spicule from *P. lividus* at different growth stages. Reproduced with permission from Beniash et al.^133^ Copyright 1997 The Royal Society. (d) Initially (20 h), a rhombohedral crystal of calcite begins to elongate in three places (*a*-axes indicated by arrows). (e) Later (25 h), the elongation has continued, and three radii grow along the *a*-axes. (f) Fully developed spicule (48 h). Labels: radii, *r*; body rod, *br*; anal rod, *ar*; and oral rod, *or*.

Importantly, spicule growth is achieved by delivery of ACC to the mineralization site,^75^ followed by transformation of the amorphous/poorly crystalline material to calcite with preservation of the volume and gross morphology of the spicule (see Section 3.3).^207,208^ Given that some other biogenic calcite crystals with noncrystallographic morphologies—such as the hammerhead elements of the coccoliths of *E. huxleyi* (see Section 4.3.2)—do not seem to form via an ACC precursor phase, this suggests that an ACC precursor phase is not required for morphological control of calcite in echinoderms.

The influence of the composition of the solution in which the larvae are raised on the development of the syncytium has provided further evidence that the spicule morphology is templated by the form of the encapsulating organic template. Pronounced changes in spicule morphologies are observed when the composition of the seawater in which the larvae are raised is changed, such as by reducing calcium and increasing magnesium concentrations.^503^ These appear to correlate with changes in the shape of the organic matrix in the syncytium prior to mineral nucleation. For example, conditions that swelled the organic matrix gave rise to thicker spicules, and those that stretched the matrix led to longer spicules.^503^

Evidence of templating was also obtained from a series of in vitro experiments in which PMCs extracted from the sea urchin *Strongylocentrotus purpuratus* were assembled into a syncytium, directed by a surface that had been micropatterned with lectins (Figure 15a).^504^ Single crystal calcite spicules formed within the patterned syncytium such that the *c*-axis of the crystal aligned with the long axis of the patterned cellular structures (Figure 15b). More sophisticated control over the spicule morphology was achieved by including a recombinant vascular endothelial growth factor (rVEGF), where this signaling molecule interacts with a cell-surface receptor (Figure 15c).^505^ Notably, rVEGF interacts with and shapes the cell assemblies bounding the developing mineral, not directly with the mineral itself,^505^ demonstrating that this process is controlled by the shape of the organic compartment. The spicule morphology could vary from h-shaped, H-shaped, or triradiate according to the concentration of rVEGF present. These morphologies correspond to growth along the *c*-axes (h and H shapes) or *a*-axes (triradiate) and were templated by the shape of the in vitro syncytium assembled by the rVEGF directed cells.

**Figure 15:**
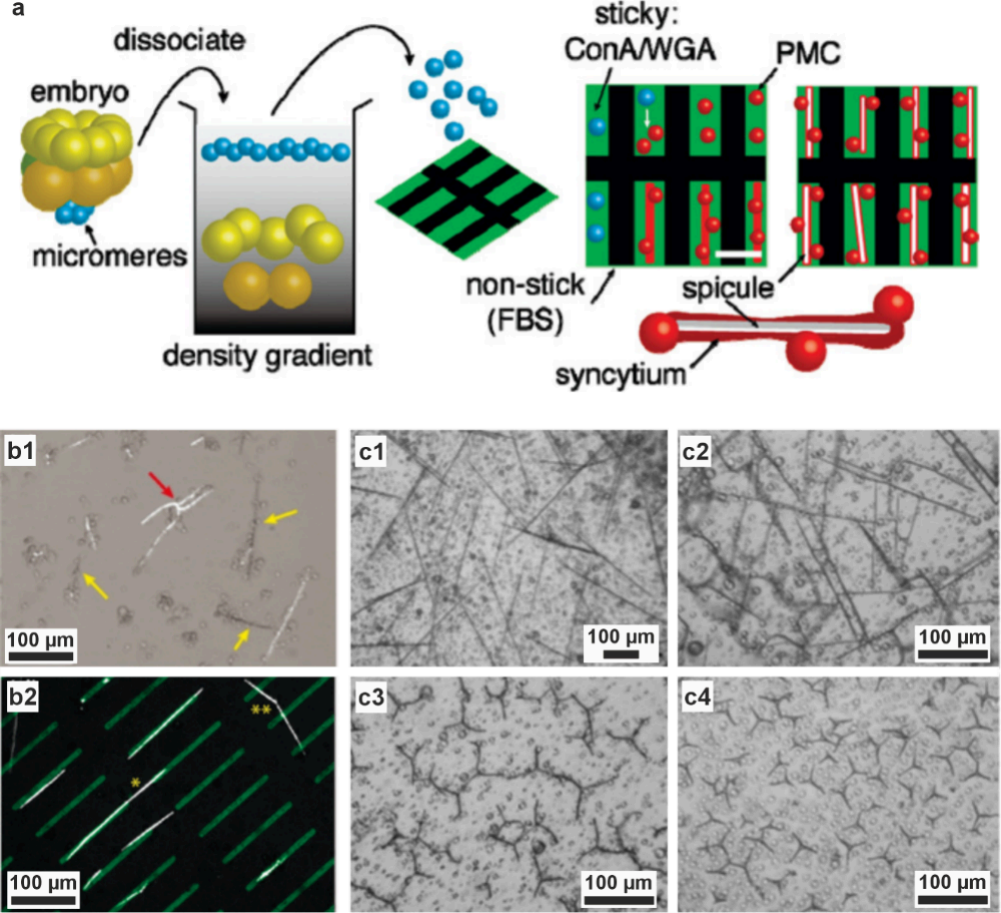
**Patterning of calcite spicules using extracted PMCs.** (a) Schematic diagram and (b) optical microscopy images of the patterning process. Reproduced with permission from Wu et al.^504^ Copyright 2011 American Chemical Society. (a) Micromeres (ascendants of PMCs, blue) were isolated from *Strongylocentrotus purpuratus* embryos and attached to microcontact printed patterns of lectins (ConA/WGA, green) on a “nonstick” fetal bovine serum (FBS) background (black). The micromeres differentiated into PMCs (red) and fused to form a syncytium that templated the formation of a single crystal calcite (CaCO_3_) spicule that was aligned with the underlying lectin pattern. (b1) Spicules on the unpatterned surface appear as single crystals (yellow arrows) that can branch (red arrow) and show no preferred orientation. (b2) Spicules (white) formed on the patterned lectins (green fluorescence) align with the pattern but can bridge along the long axis (labeled *) or across the pattern (labeled **). (c) Optical microscope images showing the effect of increasing concentrations of rVEGF on *S. purpuratus* PMCs. Reproduced with permission from Knapp et al.^505^ Copyright 2012 American Chemical Society. Spicules were (1) linear at 5 μg mL^–1^, (2) “h” shaped 15 μg mL^–1^, (3) larger triradiate at 30 μg mL^–1^, and (4) smaller triradiate at 120 μg mL^–1^.

The truly remarkable morphologies of the skeletal elements of adult echinoderms have fascinated for decades (Figure 16).^506–508^ However, their morphological development is much more difficult to study, leaving significant questions about the origins of the gross morphology. Each spine or skeletal plate (stereom) comprises a large single crystal of magnesium-bearing calcite with a fenestrated, bicontinuous form, smooth curved surfaces and pores of diameter ≈15 μm. Studies of regenerating sea urchin spines showed that, in common with larval spicules, they form via an ACC precursor phase,^55,509–511^ and that their morphologies are defined by a bounding organic sheath extending from the mineralizing cells.^510^

**Figure 16:**
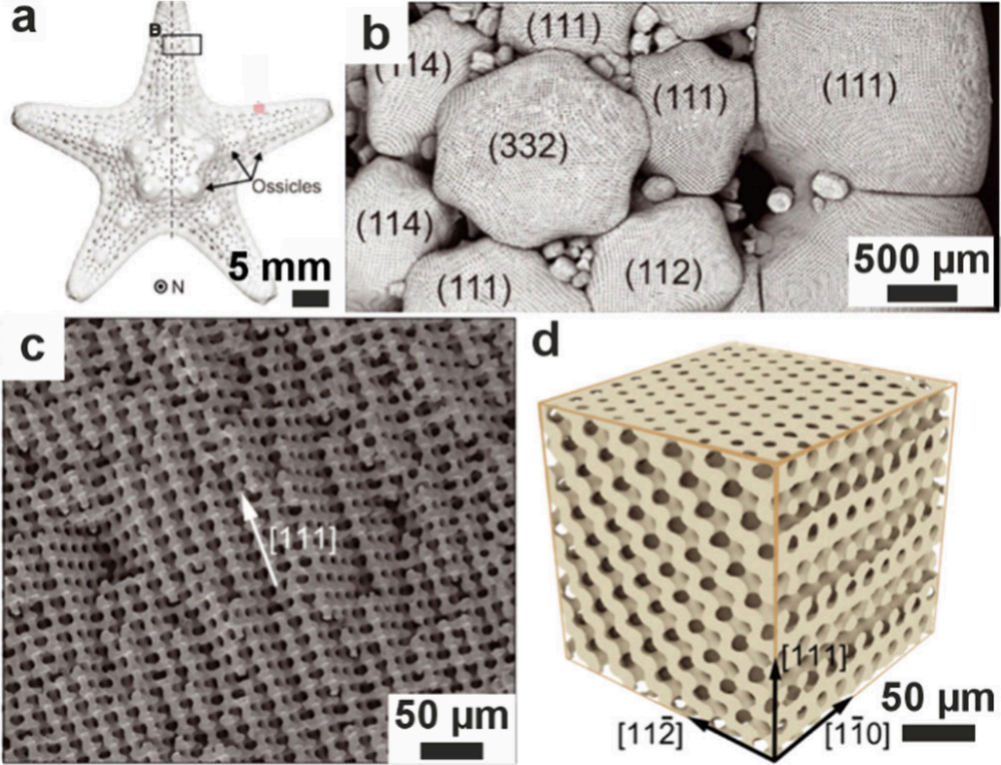
**Microlattice of the starfish** ***Protoreaster nodosus*.** Reproduced with permission from Yang et al.^56^ Copyright 2022 The American Association for the Advancement of Science. (a) *P. nodosus* after the removal of organic matter. (b) SEM image of ossicles labeled with lattice directions for each ossicle. (c) SEM image of the fractured ossicle surface. (d) 3D rendering of representative ossicle showing the fenestrated structure and orthogonal edges of [111], [110] and [112] directions of calcite.

These morphologies are characteristic of all echinoderms, and their degree of order varies according to the species and the location in the plate or spine. Notably, some species generate a stereom that resembles one of the three simplest triply periodic minimal surfaces (TPMS). TPMS occur in a range of biological, natural and synthetic systems such as insect cuticles, cell membranes, zeolites, and liquid crystal systems.^512–514^ Length-scales of ≈300 nm are observed in iridescent butterfly wings and weevil carapaces, and these are constructed from chitin structures templated by interaction with their endoplasmic reticulum, generating gyroid morphologies that mirror these folded organic membranes. The resemblance of the skeletal plates of *Cidaris rugosa* to the TPMS cubic *P*-surface was noted as early as the 1960s.^340^ More recently, the starfish *Protoreaster nodosus* was reported to generate plates that resemble the diamond-like lattice of the TPMS *G*-surface, (Figure 16)^56^ where the lattice parameter of the echinoderm TPMS is almost 4 orders of magnitude greater than that of calcite. The pores act as pathways for the organism to extend its pseudopodal tube feet^324,515^ to attach muscles fibers or allow penetration of structural collagenous fibers.^516^ This means that the pore positions may be controlled by the structure of the underlying organic systems of the organism, and has been used to reconstruct the soft parts of echinoderms from their fossil tests.^517,518^ What drives the organic matrix to adopt this shape and so template these complex crystal morphologies is currently unknown.

#### 4.3.4 Learning from Synthetic Calcium Carbonate Systems

The remarkable mechanical properties of nacre have inspired efforts to create analogous structures synthetically.^519,520^ Although the shape of nacre tablets is consistent with the orthorhombic symmetry of aragonite, aragonite typically forms as needles or elongated prismatic crystals in abiotic conditions.^12^ These morphologies could theoretically be converted to plates using soluble additives that bind to the {001} faces, retarding growth in this direction. However, as far as we are aware, this has never been achieved synthetically. Single crystal aragonite tablets have been generated synthetically using a PILP-based process, in which calcium carbonate was precipitated under Langmuir monolayers of resorcarene in the presence of pAA and magnesium ions.^521^ Thin films of calcium carbonate formed under the monolayers that comprised both aragonite single crystal tablets and calcite aggregates with thicknesses of ≈600 nm.

Calcium carbonate structures that resemble nacre have also been formed by remineralizing previously demineralized insoluble organic matrices extracted from nacre in the presence of pAsp,^522^ where ACC formed within the matrix before crystallizing into platelets (Figure 17a, b). However, while the product material morphologically resembled nacre, demonstrating the morphological control of the natural template, the mineral was calcite rather than aragonite, and the platelets were not crystallographically oriented.^522^ Precipitation of aragonite within a lamellar β-chitin organic matrix^523^ in the presence of magnesium ions gave a superior mimic of nacre, where the material was further strengthened by silk fibroin infiltration and hot pressing after calcium carbonate formation. This material resembled the brick-and-mortar structure of nacre (Figure 17c–f) and comprised aragonite platelets that diffracted individually as single crystals but were not crystallographically co-oriented. A detailed discussion of the crystallographic orientation of nacre can be found in Section 5.1.1.

**Figure 17:**
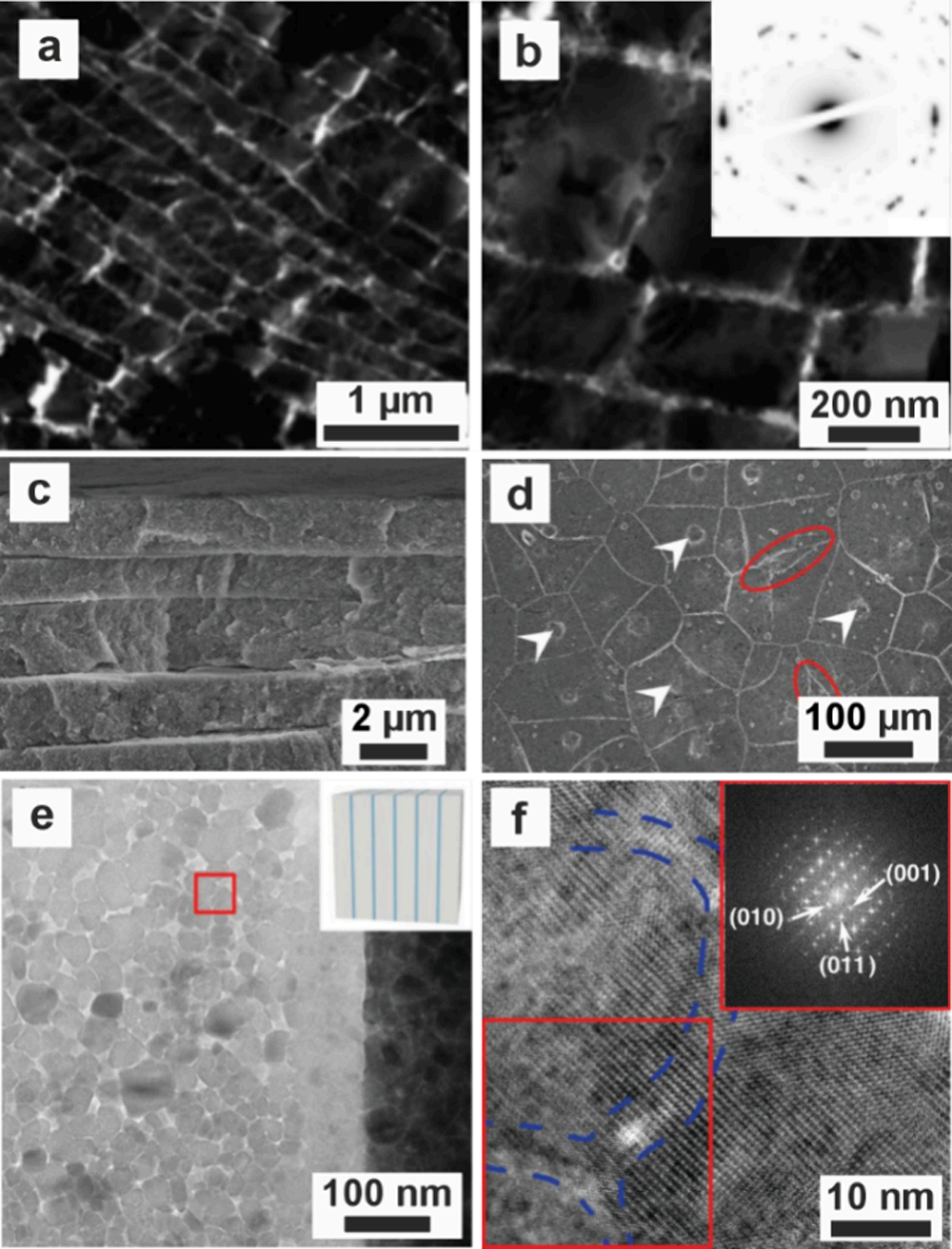
**Nacre-like structures synthesized under biomimetic conditions.** Organic and calcium carbonate composite materials were synthesized by (a, b) remineralizing the insoluble organic matrix extracted from nacre and (c–f) by depositing CaCO_3_ within a lamellar β-chitin organic matrix. (a, b) Reproduced with permission from Gehrke et al.^522^ Copyright 2005 American Chemical Society. (c–f) Reproduced with permission from Mao et al.^523^ Copyright 2016 The American Association for the Advancement of Science. (a, b) TEM images showing the cross section of the remineralized nacre. The inset SAED shows that the platelets are randomly oriented calcite crystals. (c, d) SEM images of the mineralized lamellar β-chitin in (c) cross-sectional and (d) top-down views. White arrows in (d) highlight concave areas that may correspond to mineral nucleation sites, while red ellipsoids indicate the imperfection of the Voronoi pattern. (e) TEM images of the cross section of the mineralized lamellar β-chitin, showing the nanograins. (f) HRTEM image of the selected area in (e). The inset shows the FFT pattern of this crystal.

Significant insight into the ability of organisms such as coccoliths and echinoderms to generate single crystals with nonequilibrium crystallographic morphologies has been gained from a range of synthetic systems. Experiments in which calcite crystals were grown within porous polymer membranes that had been cast from sea urchin skeletal plates—and therefore have the same size and shape as the original bicontinuous biomineral—yielded single crystals of calcite with identical curved surfaces and shapes to the original biomineral (Figure 18).^123,524–526^ Similar results were obtained with colloidal crystal templates, which also provided access to smaller length-scales.^153^ Notably, no ACC precursor phase was required to achieve these morphologies, and extension to a range of alternative compounds showed that this approach can be used to mold the form of any crystal which grows to a size larger than the length scale of the template.^525^ That the templated crystal grows to fill the entire template shows that a thin film of solution must remain between the crystal and the template so that ions can reach the growing surfaces.^527–529^ This is also the origin of the crystallization pressure that contributes to weathering in porous media.^530^

**Figure 18:**
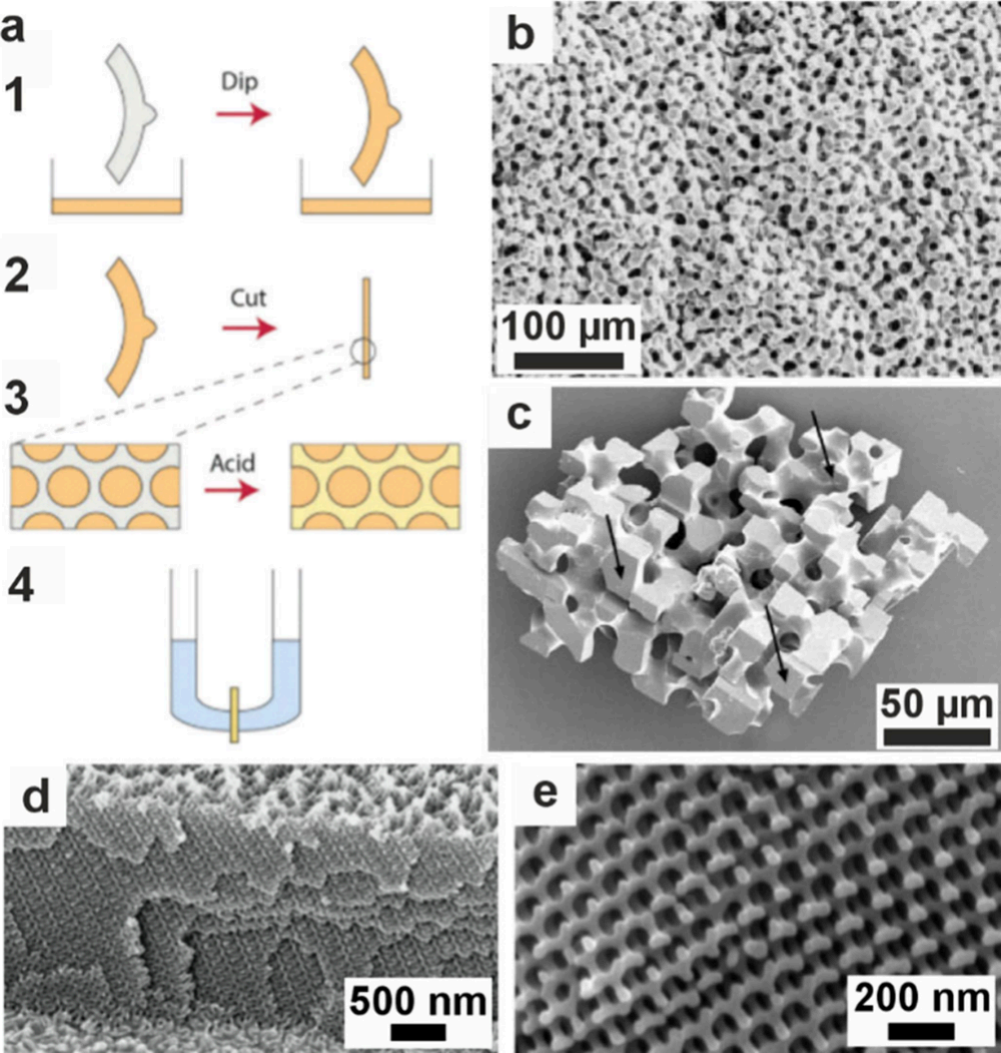
**Templated calcite single crystals.** (a–c) Calcite single crystals templated by a polymer replica of anechinoderm test. Reproduced with permission from Park and Meldrum^123^ Copyright 2002 WILEY-VCH Verlag GmbH & Co. KGaA, Weinheim. (a) Scheme of the templating method: (1) The urchin plate is dipped in polymer and cured. (2) A thin section is cut, (3) the section is exposed to acid to remove the CaCO_3_, and (4) CaCO_3_ is precipitated within the polymer replica using a double diffusion setup. SEM images of (b) a sea urchin skeletal plate and (c) the porous calcite single crystal templated on the micrometer scale. (d, e) Calcite single crystals templated by TPMS block copolymer scaffolds. Reproduced with permission from Finnemore et al.^124^ Copyright 2009 WILEY-VCH Verlag GmbH & Co. KGaA, Weinheim. (d) SEM image of patterned polystyrene template after polyisoprene removal and (e) calcite single crystal templated on the nanoscale using the structure shown in (d) after removal of the polymer.

Templated single crystals have also been generated from amorphous precursor phases, where this strategy appears to enable the templating of smaller structures than those achievable using ion-by-ion growth methods.^124,139,531^ Calcite single crystals with bicontinuous structures comparable to sea urchin skeletal plates, but with much smaller feature length-scales of ≈50 nm, were formed by generating ACC within a bicontinuous polymer membrane and then allowing it to crystallize.^124^ The presence of methanol in the solvent in this system facilitated the formation of long-lived ACC that later crystallized to give single crystals with curved surfaces. PILP phases have also been used as precursor phases and can significantly aid the infiltration of ACC into small volumes.^152^ Calcite single crystal nanorods were formed within the rod-shaped pores of TE membranes by immersing the membrane in a reaction solution in which PILP was formed,^190^ but crystal formation within nanoscale pores was limited in the absence of pAA (and thus PILP).

It is noted, however, that all of these bioinspired experiments employed rigid templates. In contrast, the organic compartments in which biominerals form can vary from readily deformable (e.g., fluid membranes) to quite stiff (e.g., assembled polysaccharide structures). The extent to which a soft template can modify the shape of a growing crystal remains unclear. While many experiments have been performed to grow polycrystalline materials within soft templates,^532–535^ we are not aware of any in which single crystals have been grown to fill the entire volume. It is therefore not known whether the crystal would maintain crystallographic planes, or whether its morphology would be dictated by the shape of the template.

A major distinction between biomineralization processes and bioinspired experiments also lies in the supply of material to the growing crystals. Organisms can continuously supply ions or amorphous nanoparticles to developing crystals from the surrounding matrix, ultimately generating a crystal that fills the entire confined volume. This contrasts with synthetic confined systems, in which the surrounding matrix is usually impermeable, and reagents can only be introduced in specific locations and/or times. It would therefore be very difficult to grow a crystal in a closed synthetic vesicle such that it fills the entire volume. Crystallization in bioinspired systems also invariably occurs in the presence of counterions, which can reach very high concentrations when larger crystals are grown. Again, organisms can control the composition of the reaction medium such that this does not occur during biomineralization, by pumping ions in and out to maintain ionic strength and pH.

### 4.4 Chiton Radular Teeth and the Stomatopod Dactyl Club

An organic matrix can also direct the morphology of polycrystalline biominerals on the millimeter or even larger scale. This is illustrated here for the radular teeth of chitons and the dactyl club of stomatopods, where in both cases the morphology is defined by the shape and gel-like nature of a preformed organic template. This organic matrix confines reactions, slows diffusion, and helps the organism control mineralization.

#### 4.4.1 Macroscopic Shaping in Chiton Radular Teeth and the Stomatopod Dactyl Club

In common with most marine mollusks, chitons use a conveyor belt-like radula for feeding (Figure 19).^536^ This consists of multiple teeth that form at the posterior of the radula and then slowly migrate to the anterior as the teeth mature.^4,537^ The teeth form in an organ called the radula sac that contains a gel-like ECM of α-chitin fibers^538^ and has the same morphology as the mature teeth (Figure 19).^539,540^ The posterior region of the organic matrix is first mineralized with magnetite (Figure 19d), and then the anterior region is mineralized with apatite after teeth pass the superior epithelium (Figure 19e). The confinement of the gel-like ECM therefore plays a dual role, where it both defines the overall morphology of the tooth and allows the organism to localize the biomineralization of magnetite and apatite. The role of an α-chitin fiber matrix in templating the orientation of the minerals in the teeth of limpets^541^ is described in Section 5.3.

**Figure 19:**
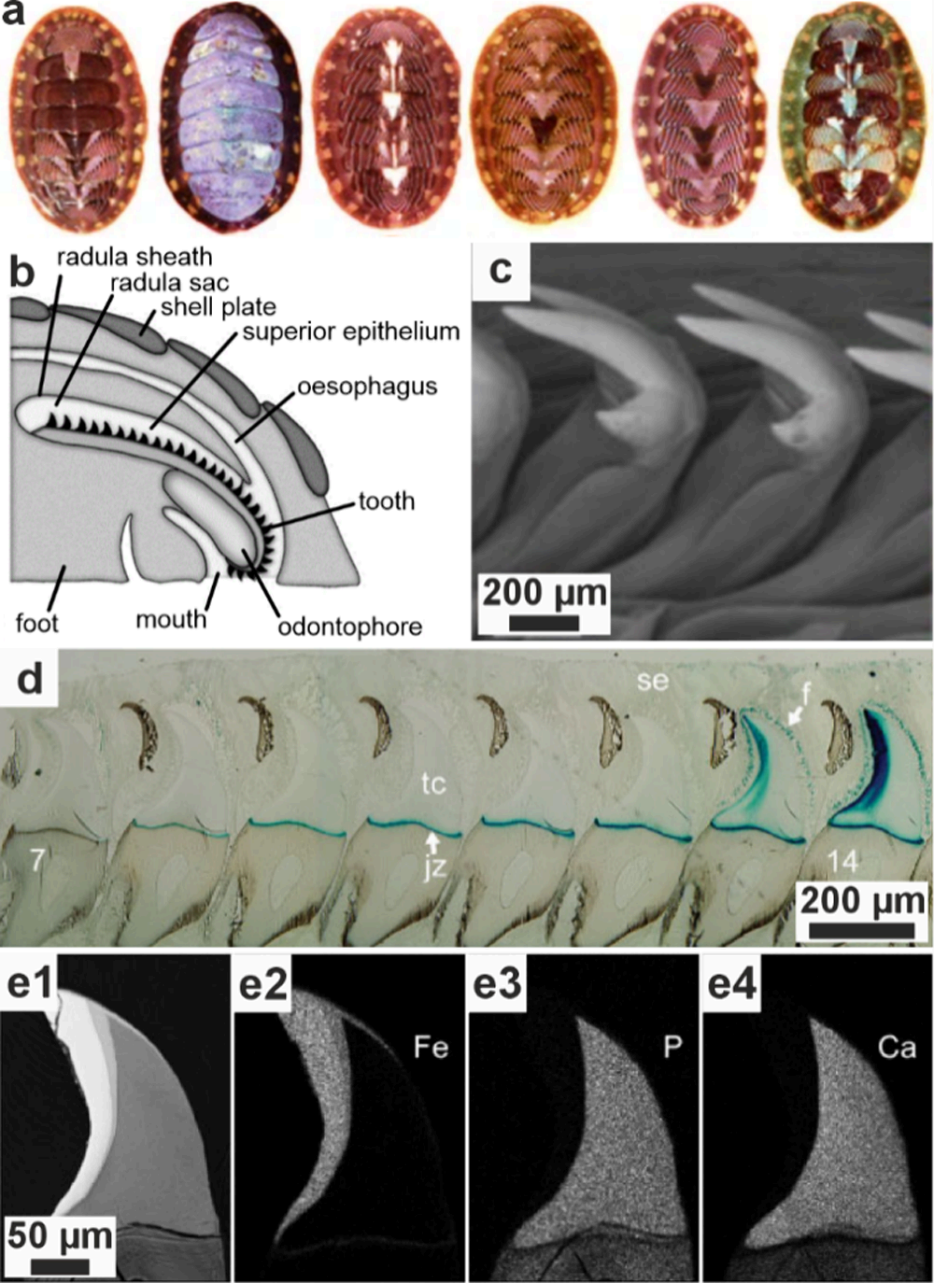
**Morphology of the radular teeth of chiton.** (a) Images of the chiton *Tonicella lineata*. Reproduced with permission from Sigwart et al.^542^ Copyright 2018 The Author(s) and Springer Nature with CC-BY license. (b) Scheme of the anatomy of a chiton radula. Reproduced with permission from Shaw and Macey.^539^ Copyright 2009 Microscopy Society of America. (c) SEM image of the major teeth of *Cryptochiton stelleri*. Reproduced with permission from Weaver et al.^4^ Copyright 2010 Elsevier Ltd. (d) OM image showing a longitudinal section of tooth cusps and associated superior epithelium from the radula of *Acanthopleura hirtosa* (early teeth 7–14). The sample is stained to show ferric iron (Fe^3+^), i.e., iron accumulation, in blue, which occurs prior to apatite deposition. Labels: tooth cusp, *tc*; junction zone, *jz*; superior epithelium, *se*; and iron-containing granules, *f*. (e) SEM image (1) and EDX elemental maps of iron (2), phosphorus (3), and calcium (4) in mature tooth 61, with elemental maps (e2) indicating the location of magnetite on the tooth leading edge and (e3–4) that of apatite (calcium phosphate) in the tooth core. (d, e) Reproduced with permission from Shaw et al.^540^ Copyright 2008 Wiley-Liss, Inc.

Considering then stomatopods (mantis shrimp), some species have developed club-like dactyl claws that are used to smash hard materials like protective shells to eat the soft animal within (Figure 20).^5,543^ The denser part of the club can be divided into impact, periodic, and striated regions (Figure 20b), all of which are constructed from helicoidally organized chitin fibers.^5^ The impact region is heavily mineralized with oriented fluorapatite nanocrystals, while the periodic and striated regions principally contain ACP and ACC.^5,544^ The formation process of the club was revealed by monitoring the molting (ecdysis) process of the animal. An organic membrane of chitin and protein is stored in the internal cavity of the old club^545^ and then unfolds and expands after ecdysis to form an unmineralized envelope with the same shape as the new club. Mineralization then begins at the inner surface of the membrane, where club mineralization protein 1 (CMP-1) nucleates apatite, probably by condensing Ca^2+^ ions and promoting the crystallization of HAp from ACP. The mineralization front progresses inward as chitin fibers are deposited ahead of the mineralization front. The shape of the membrane and the self-assembled chitin and protein organic matrix within the club envelope controls the morphology of the biomineral.

**Figure 20:**
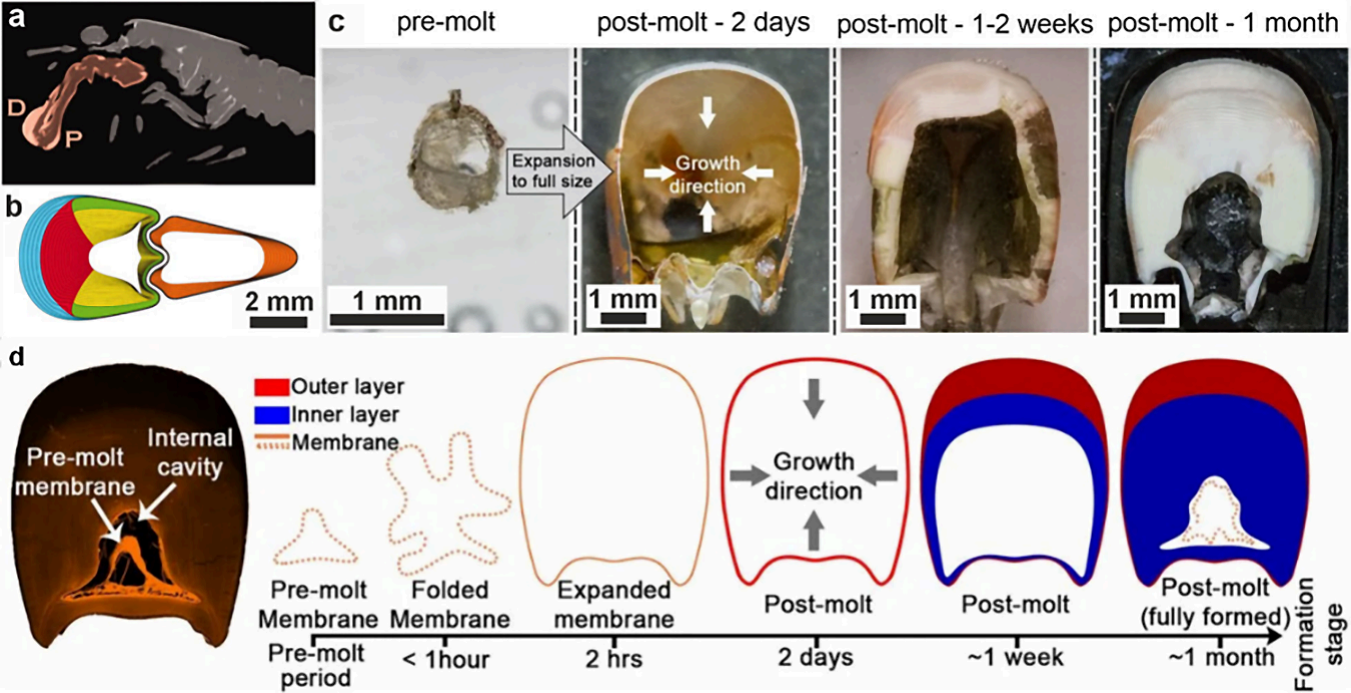
**Morphology of the dactyl club of a stomatopod**. (a) A tomographic section of the dactyl (labeled *D*) and propodus (labeled *P*) segments, revealing the difference in electron densities. (b) Cross-sectional view of the club showing the impact region (blue), the medial (red) and lateral (yellow) periodical regions, the striated region (green), and the propodus (orange). (a, b) Reproduced with permission from Weaver et al.^5^ Copyright 2012 The American Association for the Advancement of Science. (c) Optical micrographs of cross sections of dactyl clubs at different time points of the molting and formation process. (d) Diagram showing the stages of the formation process of the dactyl club during the first month after ecdysis as the membrane is mineralized to form the new club. (c, d) Reproduced with permission from Amini et al.^545^ Copyright 2019 National Academy of Science under the PNAS license.

#### 4.4.2 Learning from Synthetic Composite Systems

Molding crystals at a macroscopic level requires large-scale and controlled synthesis of both the organic templates and the crystals. The former can be readily achieved using 3D-printing, where materials such as structured bioceramics have been manufactured.^546,547^ For example, wax templates with different shapes were 3D-printed and then impregnated with a slurry of HAp nanocrystals, which filled in the voids around the wax template.^547^ Removal of the wax template by sintering generated porous bioceramics with shapes tailored for use in bone repair. Calcium carbonate structures containing pores ranging from hundreds of nanometers to millimeters in size have also been formed by extruding a concentrated, viscous suspension of surfactant-stabilized oil-in-water emulsions.^532^ The aqueous phase contained CaCl_2_ and PVA, and extrusion into a Na_2_CO_3_ solution resulted in gelation of the PVA and precipitation of calcium carbonate from the aqueous phase.^532^ The surface of the oil droplets may facilitate mineral nucleation as the surfactant accumulates calcium ions to stabilize the interface.^532^ These gels could be molded into the desired centimeter-sized forms by 3D printing the extruded mixture, and the mass of mineral was increased by alternately immersing the structures in a calcium then a carbonate solution. After molding, these materials were dried to remove the volatile oil, leaving behind porous calcium carbonate mineral foams.^532^

As a final example, Liu et al. (2019)^548^ created a highly viscous moldable precursor comprising calcium carbonate oligomers and triethylamine in ethanol. This amorphous material could be molded into centimeter-sized structures and subsequently transformed into single crystals of calcite by removal of the triethylamine and ethanol by evaporation.^548^ Removal of the triethylamine and ethanol initiated cross-linking of the calcium carbonate oligomers, leading to crystal formation as the organic component was removed.^548^ These examples demonstrate the potential of combining advanced manufacturing strategies with novel crystallization methods to achieve control over morphology and structure over multiple length scales.

## 5 Controlling Mineral Orientation

A characteristic feature of many biominerals is the high degree of orientation of the component crystals.^549–554^ These range from single crystal biominerals, such as sea urchin spines, where the calcite crystal is oriented with its *c*-axis parallel to the long axis of the spine,^549^ to polycrystalline biominerals with complex structures such as bone^552,553^ and nacre.^554^ Nacre in particular is often considered a model biomineral when discussing orientation, where it is constructed from aragonite tablets that are stacked either in a bricks-and-mortar structure or as adjacent towers.

Orientation in biominerals is often attributed to the influence of organized organic matrices that may select a specific crystal nucleation face^555,556^ or its in-plane orientation, such that all crystals nucleated on the matrix are coaligned. Epitaxial crystal growth is widely exploited in materials science, for example in the generation of thin films or seeding nucleation,^557^ and there are multiple examples of synthetic organic matrices supporting epitaxial crystallization. Langmuir-Schaeffer monolayers of 10,12-pentacosadiynoic acid have been used to direct the formation of calcite crystals nucleated from their {012} faces and crystallographically aligned with respect to the polymer backbone.^51^ Oriented crystal arrays of PbS^541^ and CdS^50^ were formed under Langmuir monolayers of arachidic acid, where this was attributed to an epitaxial match between the crystals and the monolayer, and coaligned calcite crystals were formed on gold surfaces functionalized with SAMs.^54,558^ Modeling studies attribute the latter to epitaxial and charge density matching between the SAM and crystal nucleation face^559^ and suggest that this is supported by the flexibility of the monolayer.^560^

Epitaxial matching between an organic matrix and the nucleating crystal has also been proposed to orient nacre. It was initially suggested that the organic matrix comprised a thin layer of highly ordered β-chitin that is sandwiched between two thicker layers of silk fibroin-like β-sheet proteins, onto which acidic macromolecules are adsorbed.^34^ The chitin polymers and the protein chains were considered to be orthogonally aligned,^561^ and the acidic proteins were proposed to induce the nucleation and orientation of aragonite by organizing Ca^2+^ ions such that they match the {001} face of aragonite.^34,562^ However, this model was later shown to be incorrect when cryo-TEM of hydrated samples demonstrated that the silk-like proteins actually form a disordered gel, and the acidic macromolecules are adsorbed in discrete domains on the β-chitin sheets (Figure 12a).^43,473^ While the mechanism that operates to give orientation in the mollusk shell remains unproven, control of the initial nucleation event by an organic matrix seems to be the most likely scenario.^455^ It is also noted that Pif80, an acidic protein extracted from nacre, can selectively form aragonite such that it nucleates from the (001) face when Pif80 is adsorbed to a chitin membrane via Pif97.^59^ As such, biomolecules are likely involved in orienting biomineral crystals, but they alone are not sufficient to fully control crystal orientation in vivo.

When compared to the evident role that confinement plays in defining the morphology of biominerals, its impact on crystallographic orientation has attracted much less attention. This section explores the influence of confinement on crystallographic orientation in biominerals and demonstrates how it can create an environment that facilitates competitive growth between adjacent crystallites, giving rise to orientation. This is illustrated by the well-studied biominerals in mollusk shell, bone and teeth, as well as the less well explored limpet radula teeth and sea squirt spicule, and potential insight is gained from a number of bioinspired systems that demonstrate the effects of confinement on crystal orientation.

### 5.1 Orientation of CaCO_3_ Crystals in Mollusks

#### 5.1.1 Orientation of Crystals in the Nacre and Prismatic Structures

The structure of mollusk shell was introduced in Section 4.3.1 when considering the role of confinement in morphogenesis. We now focus on the orientation of the crystals in two commonly studied types of mollusk shell microstructure, namely columnar calcite prisms and aragonitic nacre. A competition for space model has been used to explain the formation of prismatic microstructures.^563–565^ These comprise elongated crystals that are oriented with their long axes, and thus the direction of rapid growth, perpendicular to the plane of the growth front. However, the prisms in many of these tissues (e.g., the calcite in pinnoideans and pterioideans^564^) are surrounded by thick organic layers that control the growth of individual prisms.^455,469^ As the crystals grow within individual compartments in isolation from each other, their orientation cannot derive from a competitive growth effect. It has also been observed that the prisms in some *Pinctada* species comprise domains with an angular spread in orientation of 10–20°, which is thought to confer increased hardness to the shell.^467^ This gradual change in orientation of the crystal lattice may result from the crystallization from a nanoparticulate amorphous precursor.^467,566,567^

Aragonitic nacre possesses a high degree of orientation. In gastropods, the generally held picture is that the aragonite tablets stack in towers such that their crystallographic *c*-axes are parallel to the stacking axis. The *a*- and *b*-axes are aligned within individual stacks, but there is no in-plane coalignment between stacks.^455,568^ Bivalve nacre, in contrast, is described as a brick-and-mortar structure in which the axes of the crystals are coaligned, and the *c*-axes are oriented perpendicular to the nacre surface. However, analysis of the nacre formed by 15 mollusk species using polarization-dependent image contrast (PIC) mapping showed that the reality is rather more complicated, and an angular spread of *c*-axis orientation of about 30° was observed in all of the species analyzed.^568^

PIC imaging of nacre in the abalone gastropod *Haliotis rufescens* showed that the tablets are aligned within stacks.^465^ The misorientation of the *c*-axes in adjacent stacks is thought to be due to a kinetic effect created by competition for space (Figure 21a, b).^465^ In contrast, analysis of bivalve nacre reveals local alignment in domains of diagonally staggered tablets, and when changes in orientation occur between adjacent tablets they are often abrupt. In all species investigated, the degree of *c*-axis misorientation increases with proximity to the nacre-prismatic boundary.^568^ This effect was attributed to competitive growth within an enclosed space, such that the faster-growing tablets whose *c*-axes are aligned with the growth direction dominate.^565,568^ The alignment between tablets in individual stacks is attributed to a small mineral bridge that connects tablets across the bounding organic sheets (Figure 21e, f).^464,568^ Notably, this appears to give rise to a gradual lattice tilting between the crystals within a domain.^568^

**Figure 21:**
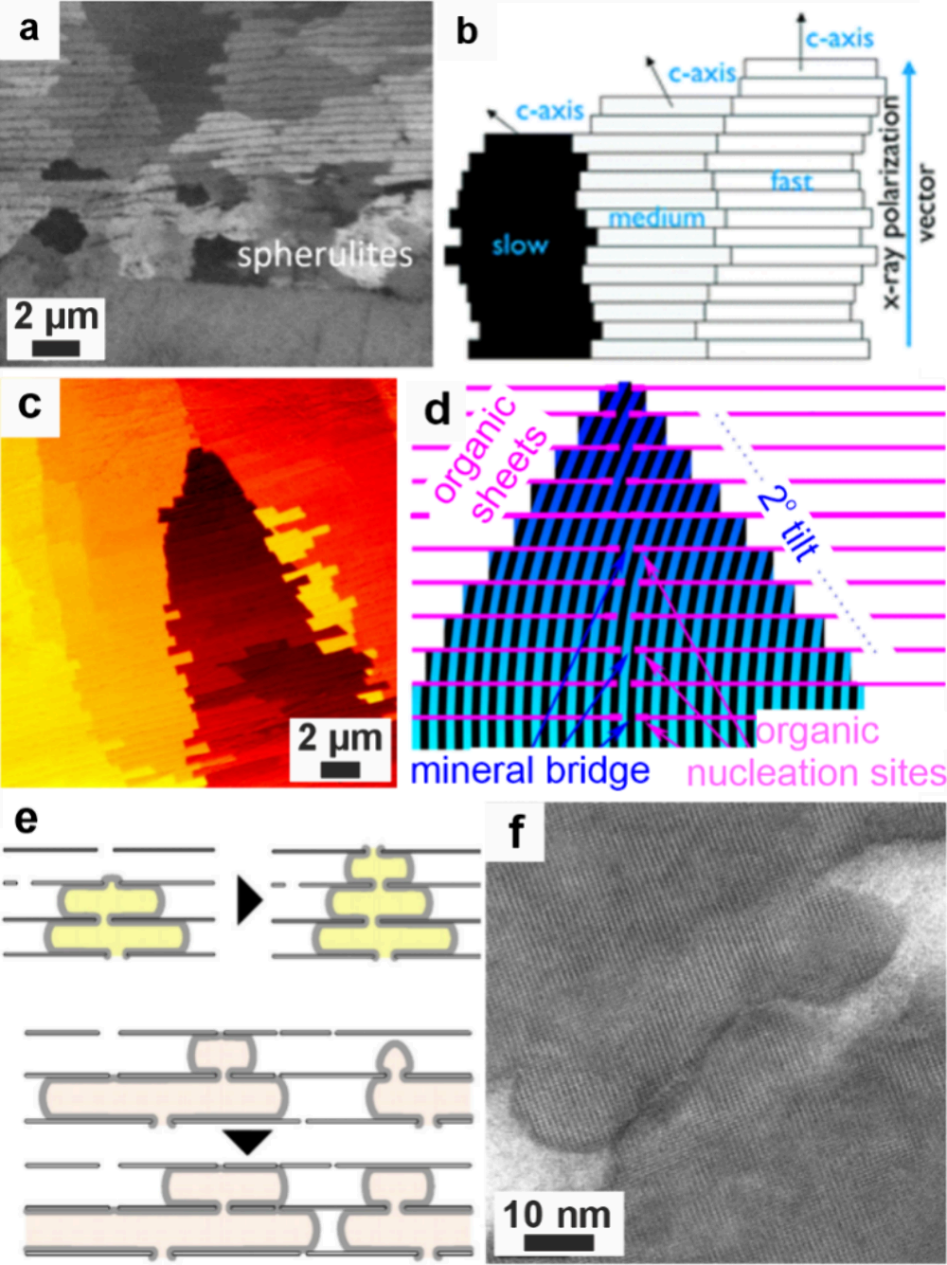
**Development of the orientation of nacre.** (a) X-PEEM observation of the nacre of red-abalone. The gray levels in this image correspond to the *c*-axis orientation of aragonite, and those with lower contrast are closer to perpendicular to the organic matrix layers. (b) Model showing the development of the *c*-axis orientation in nacre due to competition for space. (a, b) Reproduced with permission from Gilbert et al.^465^ Copyright 2008 American Chemical Society. (c) False-colored X-PEEM PIC map of nacre *Haliotis laevigata*. The gradual change of color within a stack indicates a tilting between tablets. (d) Model showing the observed tilting. (c) Reproduced and (d) redrawn with permission from Olson et al.^568^ Copyright 2013 Elsevier Inc., published by Elsevier Inc., all rights reserved. (e) Illustrations showing the growth of aragonite between organic layers in gastropod (top, yellow) and bivalve (bottom, peach) nacre organic templates. Reproduced with permission from Cartwright and Checa.^456^ Copyright 2006 The Royal Society of Chemistry. (f) TEM image showing the mineral bridge between adjacent layers of aragonite tablets in gastropod *Gibbula umbilicalis* nacre. Reproduced with permission from Checa et al.^464^ Copyright 2011 Elsevier Inc., all rights reserved.

The in-plane ordering of nacre tablets has been investigated using X-ray diffraction (XRD) and SEM and was again seen to evolve from relatively low ordering adjacent to the nacre-prismatic boundary to highly ordered more distant from it.^569^ This was also attributed to competitive growth of crystals within the confines of an individual lamella. The growth of aragonite is ≈1.6× faster along the *b*-axis than the *a*-axis, such that the crystals oriented with their *b*-axis parallel to the growth direction of the lamellae grow fastest and thus dominate (Figure 22).^569,570^ As the adjacent lamellae in the nacre structure are physically linked though mineral bridges, the orientation of the more rapidly growing crystals will eventually dominate in the mature nacre. However, Checa subsequently queried this explanation, stating that nacre tablets are not always seen to elongate along the *b*-axis.^455^

**Figure 22:**
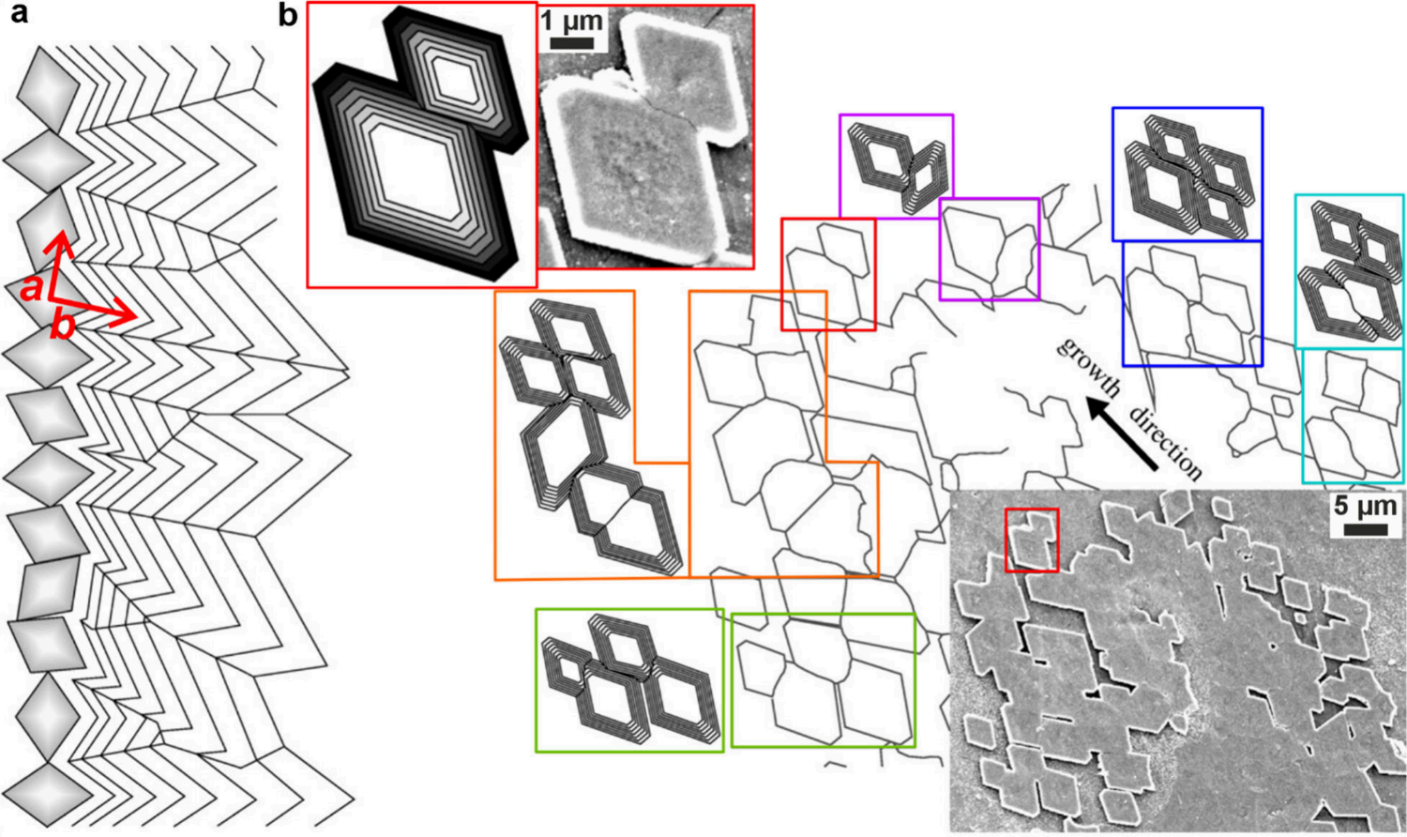
**Development of** ***a-b*** **orientation in nacre due to competitive growth.** Reproduced with permission from Checa et al.^570^ Copyright 2006 The Royal Society. (a) Schematic diagram showing the plane of the lamella, where the boundaries between growing crystals are drawn at equivalent growth increments and crystals are growing toward the right. Those with the longest *b*-axis diagonals parallel to the growth direction out-compete other orientations and survive. (b) SEM images and sketch of the growth front of a lamella from *Pteria hirundo*, showing rhombic nacre {110} faces with some {010} faces. Colored boxes highlight contacts between crystals that are consistent with the competitive growth model.

#### 5.1.2 Learning from Synthetic Calcium Carbonate Systems

Control over calcium carbonate orientation by the cylindrical nanopores of TE membranes has been studied for all of its three anhydrous polymorphs: calcite,^571^ aragonite,^98^ and vaterite.^116^ Single crystal calcite nanorods were precipitated within 70 nm diameter pores via the transformation of ACC, and lattice rotation angles of several degrees per micrometer were recorded along the length of the nanowire.^571^ This was attributed to surface stresses arising from the anisotropic crystal structure of calcite, as well as the nanoscale diameter and confined space provided by the membrane pore.^571^ This raises the interesting possibility that confinement effects contribute to the lattice tilting observed in some calcite prisms and stacks of aragonite tablets in nacre (see Section 5.1.1).

When aragonite nanorods were precipitated within pores that are 200 nm or less in diameter, their *c*-axes align with the long-axes of the pores.^98^ This is most likely due to competitive growth effects favoring aragonite crystals with their *c*-axes (which is usually the direction of fastest growth) coaligned with the long axis of the pore. Calcite^139,190,346^ and vaterite^116^ nanorods were not oriented in the pores, likely due to their lower growth rate anisotropies. Strikingly, it has proven very difficult to synthesize aragonite platelets with the *c*-axis perpendicular to the plate, as observed in nacre. A notable exception is aragonite crystals grown on chitin-coated glass slides in the presence of acidic macromolecules extracted from nacre, where the crystals formed between the glass slide and chitin membrane, and were oriented with the *c*-axis perpendicular to the substrate (Figure 23c, d).^59^

**Figure 23:**
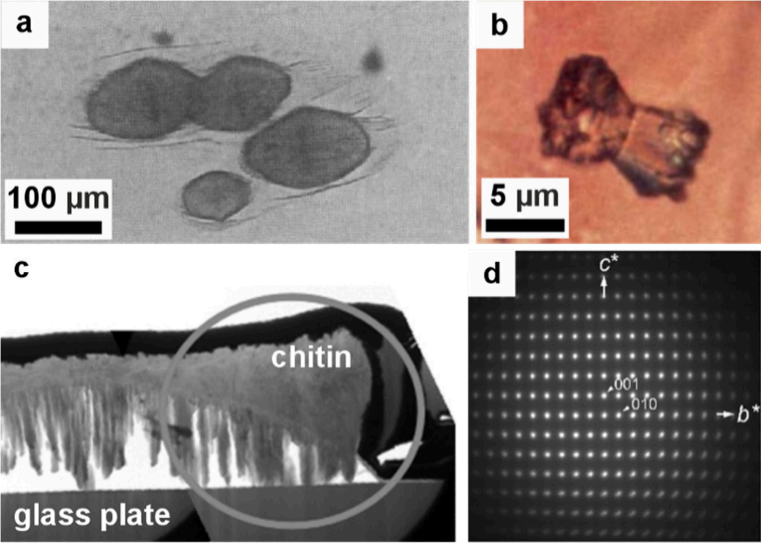
**Aragonite crystals formed in the presence of proteins extracted from aragonitic nacre.** (a) Optical micrograph of aragonite formed within a β-chitin-silk fibroin substrate supplemented with aragonite-associated glycoproteins. Reproduced with permission from Falini et al.^57^ Copyright 1996 The American Association for the Advancement of Science. (b) Optical micrograph of aragonite crystal grown on a nucleating protein sheet in the presence of polyanionic proteins isolated from the aragonitic nacre from the shell of the red abalone *Haliotis rufescens*. Reproduced with permission from Belcher et al.^60^ Copyright 1996, Springer Nature Limited. (c) TEM image of the cross section of a crystal formed in the space between the chitin membrane and a glass plate in the presence of Pif proteins. (d) Electron diffraction pattern of the area circled in (c), indicating a single aragonite crystal oriented with its *c*-axis perpendicular to the glass plate. (c, d) Reproduced with permission from Suzuki et al.^59^ Copyright 2009 The American Association for the Advancement of Science.

### 5.2 Orientation of Hydroxyapatite (HAp) Crystals in Bone and Teeth

#### 5.2.1 HAp Orientation in Bone

Vertebrate bone is a hierarchical structure organized over at least nine different levels (Figure 24a).^553,572,573^ The organic component is principally collagen, which possesses helix-forming repeating proline hydroxyproline glycine (POG) motifs and extrahelical telopeptide sequences.^574^ The collagen is secreted as procollagen, which is cleaved to release tropocollagen molecules that fold into trimeric helices. These tropocollagen trimers form the basic subunit, which then further self-assembles into fibrils.^575^ The fibrils display a characteristic ≈67 nm periodic banded structure due to the difference in contrast created by regions in which the tropocollagen trimers overlap (more dense) and those span gaps (less dense) (Figure 24b).^575^ These gaps are cylindrical and are approximately 2 nm × 2–4 nm × 20 nm and can overlap to be up to 36 nm in length, with the long axis of the void aligning with the long axis of the fibril.^383,576^

**Figure 24:**
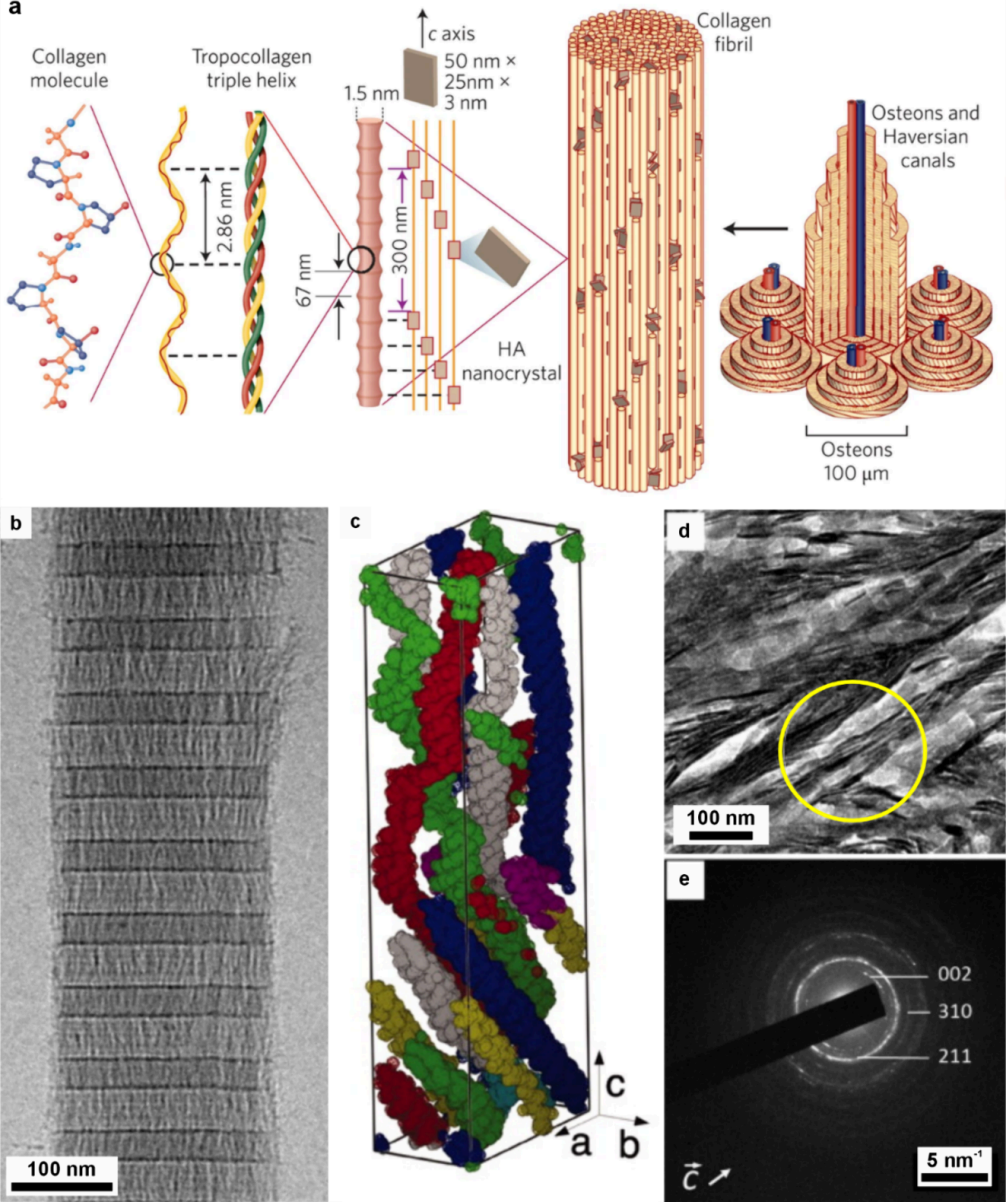
**Structure of bone.** (a) Scheme showing the hierarchical structure of bone. Reproduced with permission from Wegst et al.^18^ Copyright 2014, Springer Nature Limited. (b) Cryo-TEM image of an unmineralized bovine tendon collagen fibril showing the characteristic 67 nm banding of the protein structure. Reproduced with permission from Xu et al.^383^ Copyright 2020 of the Author(s)­and Springer Nature with a Creative Commons Attribution CC-BY 4.0 license. (c) Cα carbon structure of D-staggered collagen in the protein unit cell. Reproduced with permission from Orgel et al.^577^ Copyright 2006 National Academy of Science under the PNAS license. (d) TEM image of a focused ion beam (FIB) thin section of human bone, showing the HAp platelets. (e) SAED pattern of the area circled in (d), indicating that the *c*-axes of HAp platelets are aligned parallel to the long axis of the collagen fibril. (d, e) Reproduced with permission from Reznikov et al.^553^ Copyright 2018 of the Author(s) and The American Association for the Advancement of Science.

Collagen fibrils in bone are mineralized with carbonated HAp platelets, oriented with their *c*-axes parallel to the long axis of the collagen fibrils (Figure 25c, d).^553^ These thin (2–4 nm) crystals significantly increase the stiffness, fracture resistance, strength and robustness of the collagen,^578^ and are located both outside and within the fibrils.^579^ Here, we focus on the intrafibrillar HAp platelets, as these form within the confinement of the collagen and have received considerable attention due to their orientation.

**Figure 25:**
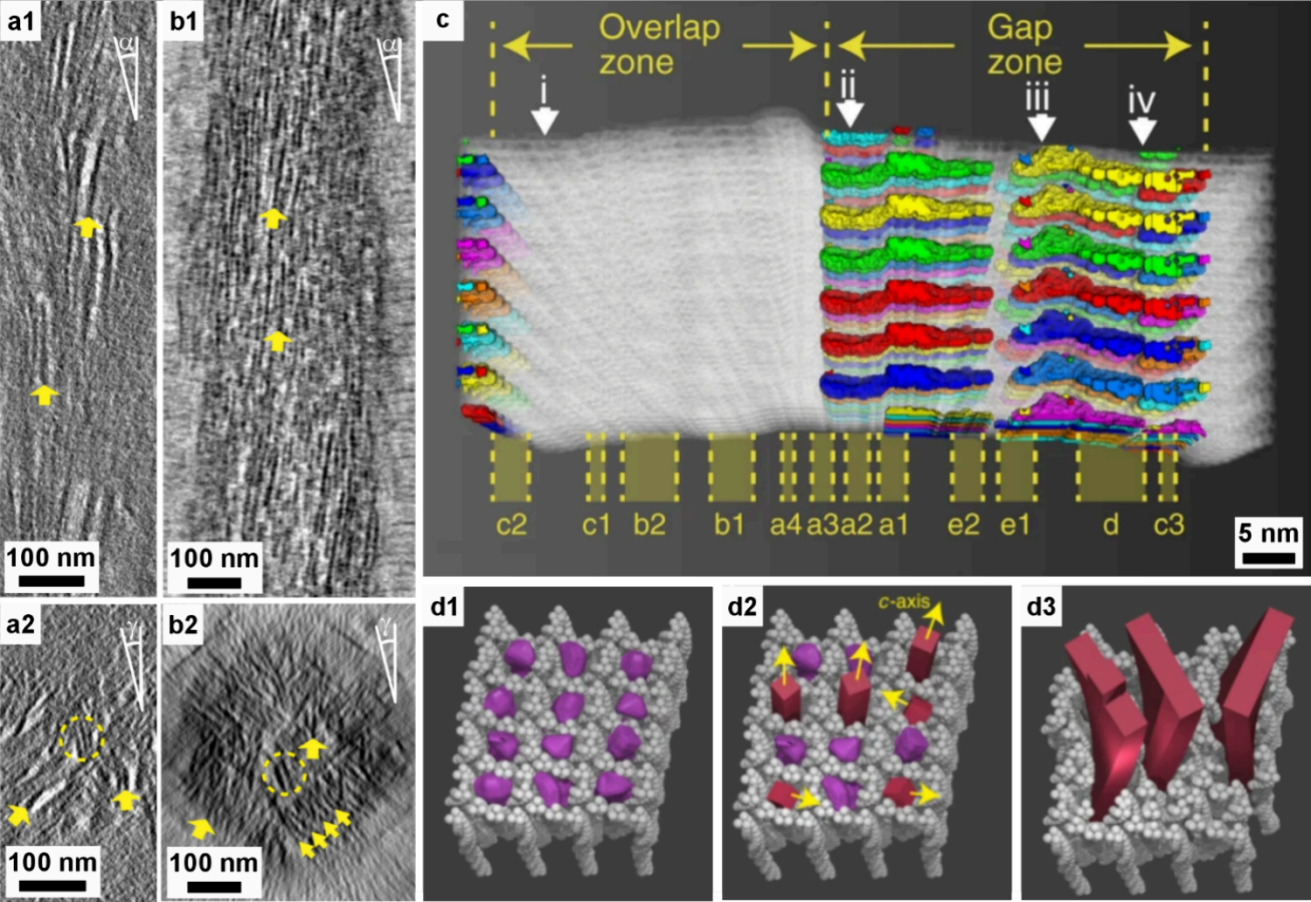
**Orientation control in mineralized collagen.** Reproduced with permission from Xu et al.^192^ Copyright 2020 of the Author(s) and Springer Nature with a Creative Commons Attribution CC-BY 4.0 license. (a1) Side and (a2) cross-section view electron tomography reconstruction slices of in vivo mineralized collagen fibril from bone, showing that the *c*-axes of HAp platelets are oriented parallel to the long axis of the collagen fibril. The platelets are not co-oriented laterally (i.e., perpendicular to the axis of the fibril) but instead form short stacks that are highlighted with a yellow circle in (a2). (b1) Side and (b2) cross-section views of slices of an in vitro remineralized horse tendon collagen fibril, showing similar mineral orientations to those in the naturally mineralized bone shown in (a). (c) A 3D electron density map generated from X-ray scattering of a hydrated rat tail collagen fibril. The intermolecular channels are highlighted with colors. (d) Model showing that initially, (d1) ACP infiltrates the gaps (purple), (d2) from which randomly oriented HAp crystal nucleate (red). (d3) Only those with their *c*-axes oriented parallel to the long axis of collagen fibril grow in the direction of least confinement.

Weiner and Traub first studied calcified turkey tendon—which contains HAp-mineralized collagen fibrils similar to bone—with TEM, and showed that the HAp platelets were aligned with their *c*-axes parallel to the long axis of the fibril and that their *a*- and *b*- axes appeared to be co-oriented.^552^ Subsequent electron tomography of calcified turkey tendon^580^ and chicken bone^581^ supported this analysis, giving rise to a model in which collagen molecules were considered as straight rods that were packed into staggered stacks. The stacks were thought to contain parallel 2D channel gap regions in which the HAp crystals nucleated and subsequently developed into platelets.^580^ The high degree of crystallographic orientation was believed to originate from epitaxial matching between the amino acid residues in the gap region and the surface of the HAp crystals.^44,580,582^

Analysis of mineralized fibrils using today’s analytical techniques has challenged some of these ideas. Collagen fibrils are now known to be constructed from microfibrils, each of which contains five twisted collagen trimers assembled in a 1D staggered offset pattern, and the microfibrils are also twisted around each other. Consequently, there are no 2D channels.^577^ X-ray scattering of the HAp platelets in fish and equine bones has also revealed that they are only co-oriented along their *c*-axes and that stacks only comprise a few platelets.^184,583^ Further challenging the possibility of an epitaxial model, NMR has shown that the HAp platelets in bone are separated from the organic template by a layer of amorphous mineral, which prevents any possible epitaxial matching between the crystals and collagen.^584^

There is growing evidence that confinement rather than epitaxial matching gives rise to the orientation of the HAp platelets in mineralized bone. Electron tomography was used to study collagen fibrils that had been mineralized in vivo and in vitro, generating 3D images of the mineral platelets embedded within the collagen fibrils.^383^ This showed that the intrafibrillar HAp platelets are present as small stacks of 2–4 crystals, and their *c*-axes are uniaxially oriented (Figure 25a, b).^383^ Some twist like propellers and have needle-shaped tips, which is consistent with mineralized human bone collagen fibrils.^553^ Notably, collagen also directed the orientation of lepidocrocite (γ-FeOOH) and vaterite (CaCO_3_) crystals such that their fast-growing *c*-axes were parallel to the long axis of the fibril, but calcite crystals, which have rhombohedral structures and no preferential growth directions, were not oriented.^383^ Importantly, analysis of X-ray scattering data^383,577^ showed that 2 × 2–4 × 20 nm cylindrical channels are present in the gap region of the collagen and are oriented with their long axes parallel to the fibril axis (Figure 25c). In combination, this demonstrates that a combination of confinement and minerals with anisotropic growth habits are required to orient minerals in collagen.

This led to a new confinement-based model where calcium phosphate PILP precursors infiltrate into the intermolecular channels in the collagen fibrils, and then start to crystallize to form HAp nuclei with random crystallographic orientations (Figure 25d).^383^ Those with their *c*-axes aligned parallel to the long axes of the intermolecular channels (and thus fibrils) grow with less restriction^383^ and develop into nanoplatelets. The growth of the misoriented nuclei is restricted by the confining collagen matrix, such that the final product is dominated by the oriented platelets. This builds on a previous model that suggested that the collagen matrix directed the transformation of a PILP phase, but which used an earlier quasi-hexagonal model of the collagen fibril^184^ and therefore could not explain the orientation of the mineral platelets.

#### 5.2.2 HAp Orientation in Tooth Enamel

Tooth enamel is another important HAp biomineral and forms the outermost layer of vertebrate teeth.^585^ It is the hardest tissue in vertebrates and comprises 95–97% HAp and 1–3% organics.^585,586^ Enamel has a truly remarkable microstructure that consists of interwoven rope-like units called rods or prisms (Figure 26).^585,587,588^ Each rod is several micrometers thick, and contains assemblies of ≈60 nm thick, micrometers-long HAp nanocrystallites.^589^ The long axes of adjacent HAp crystallites within each rod are oriented along the long axis of the rod, while crystals in the inter-rod areas are misoriented by ≈60°.^585,587,590^ The long-axes of the HAp crystallites have long been considered their *c*-axes, but a recent PIC mapping study suggests a 1–30° misorientation between the morphological long-axes and the crystallographic *c*-axes.^591^ The relationship between the morphology and orientation of these HAp crystals is therefore more complicated than first thought.

**Figure 26:**
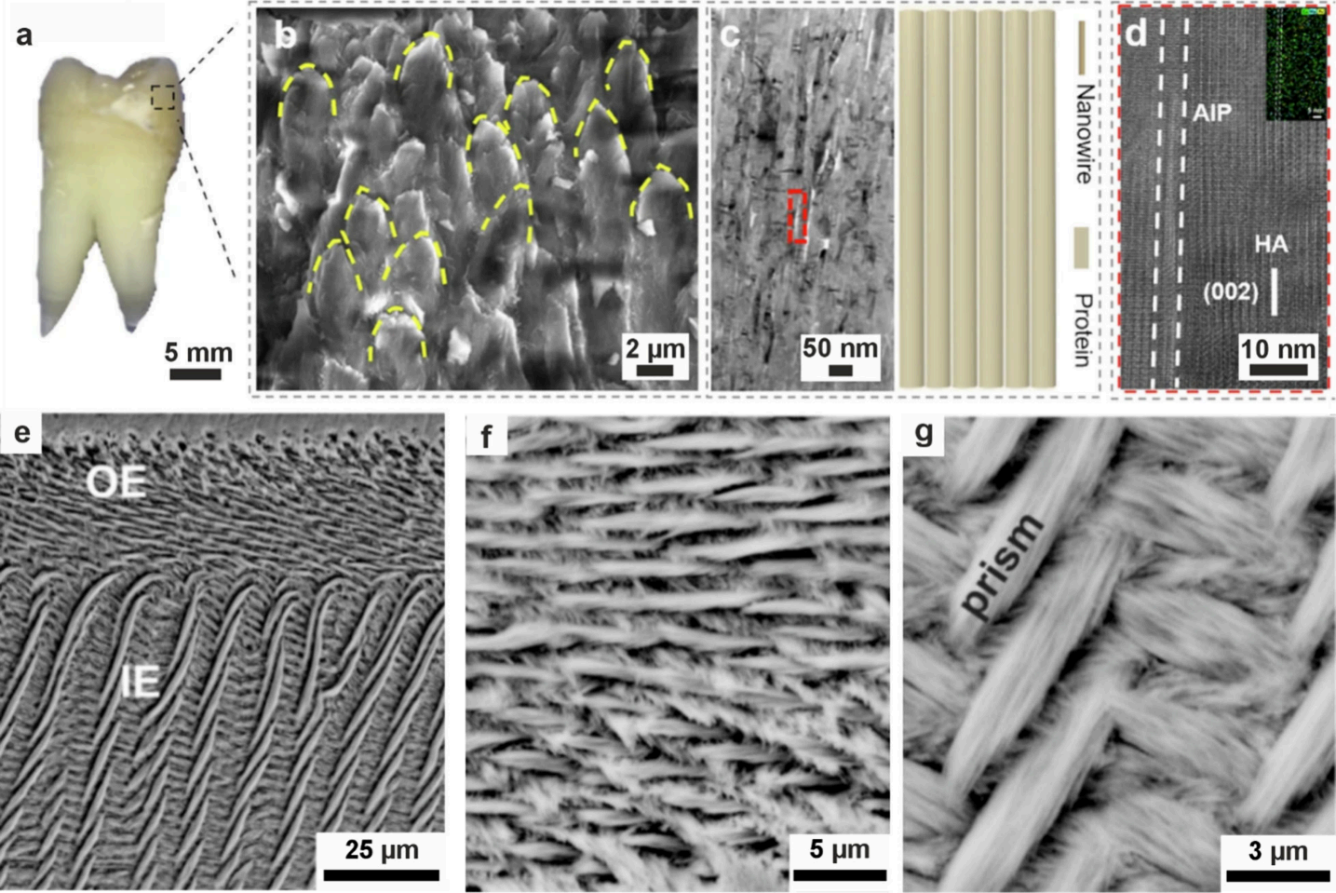
**SEM and TEM images of the rod structures in tooth enamel**. (a–d) Human tooth enamel. Reproduced with permission from Lu et al.^587^ Copyright 2024 of the Author(s) and Springer Nature with a Creative Commons Attribution CC-BY 4.0 license. (a) Optical image of a human molar and (b) SEM iamge of enamel (yellow highlights microbundles of HAp). TEM images from a section through enamel at (c) lower resolution and (d) higher resolution (red box on c), annotated with HAp (002) alignment, amorphous intergranular layer (AIP), and HAp nanowires (HA). (e–g) Rat incisor from *Rattus sp.* Reproduced with permission from Dauphin^588^ Copyright 2020 The Author(s) and Licensee MDPI, Basel, Switzerland with CC-BY-4.0 license. SEM images showing an outer prismatic sublayer (OE) and a thick inner prismatic sublayer (IE) at (e) low magnification and (f) higher magnification of OE and (g) higher magnification of IE. Crystallites of HAp are labeled *prism*.

Many questions remain concerning the mechanisms by which organisms control the orientation of the HAp crystals in enamel. The formation of tooth enamel starts with the migration of ameloblast cells from the dentin-enamel-junction area toward the enamel surface, with each cell creating an individual projection called a Tomes’ process (TP).^592^ Each of these projections delineates a confined column-shaped area of about 5 μm in diameter in which the mineral forms. Cells fill this area with proteins that are subsequently mineralized with HAp^592^ and that direct the formation of enamel.^593–597^ Each enamel rod corresponds to a TP whereas the inter-rod areas form at the interfaces between the TPs.^598^

Amelogenin constitutes ≈90% of the protein that fills the TP^599^ and plays a key role in directing the formation of HAp in enamel.^593–597^ In vitro experiments show that amelogenin can self-assemble into microribbons in aqueous solution^594^ and template the orientation of HAp crystallites, with their *c*-axes aligned with the long axes of the ribbons. It was initially proposed that this may originate from epitaxial matching between the crystal and ribbon, but later experiments showed that the ribbon is poorly crystalline,^594^ making this unlikely. It has also been reported that amelogenin can stabilize and assemble ACP clusters into linear chains, which may promote their transformation into oriented HAp crystal arrays.^595,596^ The intrafibrillar confinement provided by the amelogenin ribbons may also contribute to the orientation of HAp crystals, as shown for collagen,^383^ and the confined environment of the TPs is likely to be critical in ensuring that the amelogenin assembles correctly and in molding the final form of the enamel.^600^

#### 5.2.3 Learning from Synthetic HAp Systems

That confinement can direct the orientation of HAp crystals has also been demonstrated in simple in vitro experiments. The cylindrical nanopores in TE membranes were able to template the orientation of the *c*-axis of HAp crystals parallel to the long axis of the pore (Figure 27).^127,191^ Polycrystalline particles with no preferred orientation formed in 300 nm diameter pores, while about 75% of the particles in 200 nm pores showed some degree of preferential orientation of the *c*-axis of the crystal parallel to the long axis of the pore.^127^ Co-orientation significantly increases in 50 nm pores, such that 95% of the particles show marked orientation, while the crystals formed in 25 nm pores were highly oriented, with angular spreads of between ± 5–12°. This is comparable to the *c*-axis orientation of HAp crystallites in bone and mineralized reconstituted collagen fibrils. In the absence of any structural match between the HAp lattice and the surface of the membranes, the crystal orientation in the TE membrane pores can only be achieved by competitive growth effects induced by confinement.

**Figure 27:**
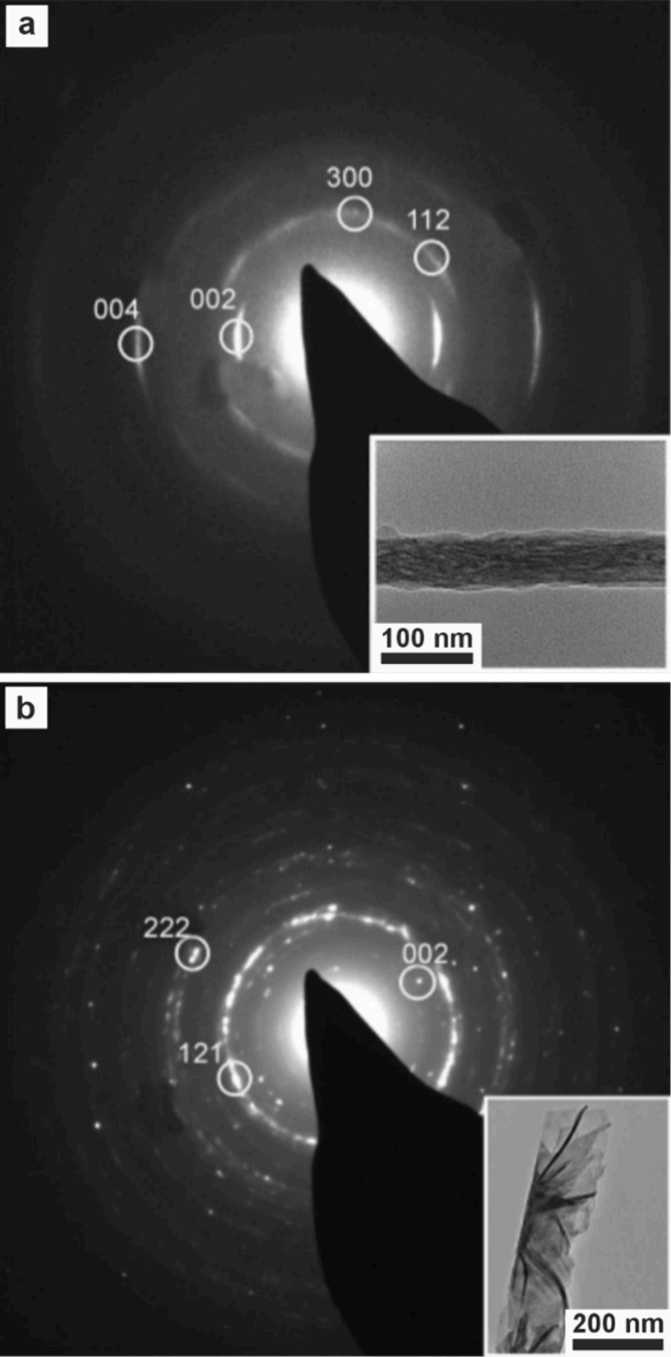
**Oriented HAp crystals formed within the pores of TE membranes.** Reproduced with permission from Cantaert et al.^127^ Copyright 2013 WILEY-VCH Verlag GmbH & Co. KGaA, Weinheim. TEM images and SAED patterns of HAp platelets precipitated within (a) 50 nm and (b) 200 nm sized pores in TE membranes, showing a decreasing *c*-axis crystallographic orientation of the crystals as confinement is reduced.

A number of materials with rod-like microstructures reminiscent of enamel have also been synthesized on solid substrates or at the air–water interface,^601–603^ further showing that competitive growth effects can give rise to these morphologies in the absence of biological control. Arrays of close-packed fluorapatite rods oriented with their *c*-axes perpendicular to the substrate were synthesized on iron plates using a hydrothermal route.^601^ Similar arrays of fluorapatite crystals were also grown at the air–water interface under Langmuir monolayers predicted to bind to the (001) face of HAp, and the orientation and thickness of the rods increased with the fluoride concentration.^603^ In a third example, transparent thin films of HAp were generated at the air–water interface when chondroitin sulfate and fluoride ions were present in the subphase.^602^ Evaporation from the air–water interface leads to enhanced supersaturation at that location, resulting in the formation of spherulites that are confined to the plane and which grow in competition with each other to give preferential orientation of the *c*-axis perpendicular to the substrate.

Of course, none of these structures replicate the complexity of the structure of enamel.^588,590^ It is therefore likely that biotemplating of amelogenin ribbons and confinement within the Tomes process act in combination to generate the complex enamel structure. Experiments that combine confinement with competitive growth in organic matrices may be able to provide a better understanding of how confinement controls HAp mineralization in bones and teeth.

### 5.3 Limpet Radula Teeth with Oriented Goethite

Limpets are aquatic gastropods that use radula teeth for feeding in a similar way to chitons (see Section 4.4.1).^604^ The teeth consist of a fibrillar α-chitin organic matrix (≈60% mass)^605,606^ that is mineralized with 15–20 nm × 200–1000 nm goethite (α-FeOOH) nanowires (≈12% mass) and amorphous silica (≈10% mass).^607–609^ The long axes of the goethite crystals correspond to their *c*-axes,^607^ as also observed in synthetic crystals,^610^ and are aligned with the long axis of the fibers in the chitinous matrix, where they form bundles. The overall orientation of these bundles is well-controlled, where two perpendicular sets of bundles exist near the leading (concave) surface and the bundles form acute angles with the trailing (convex) surface (Figure 28a).^611^ The orientations define the wearing mode of the teeth and contribute to their self-sharpening properties.^611^

**Figure 28:**
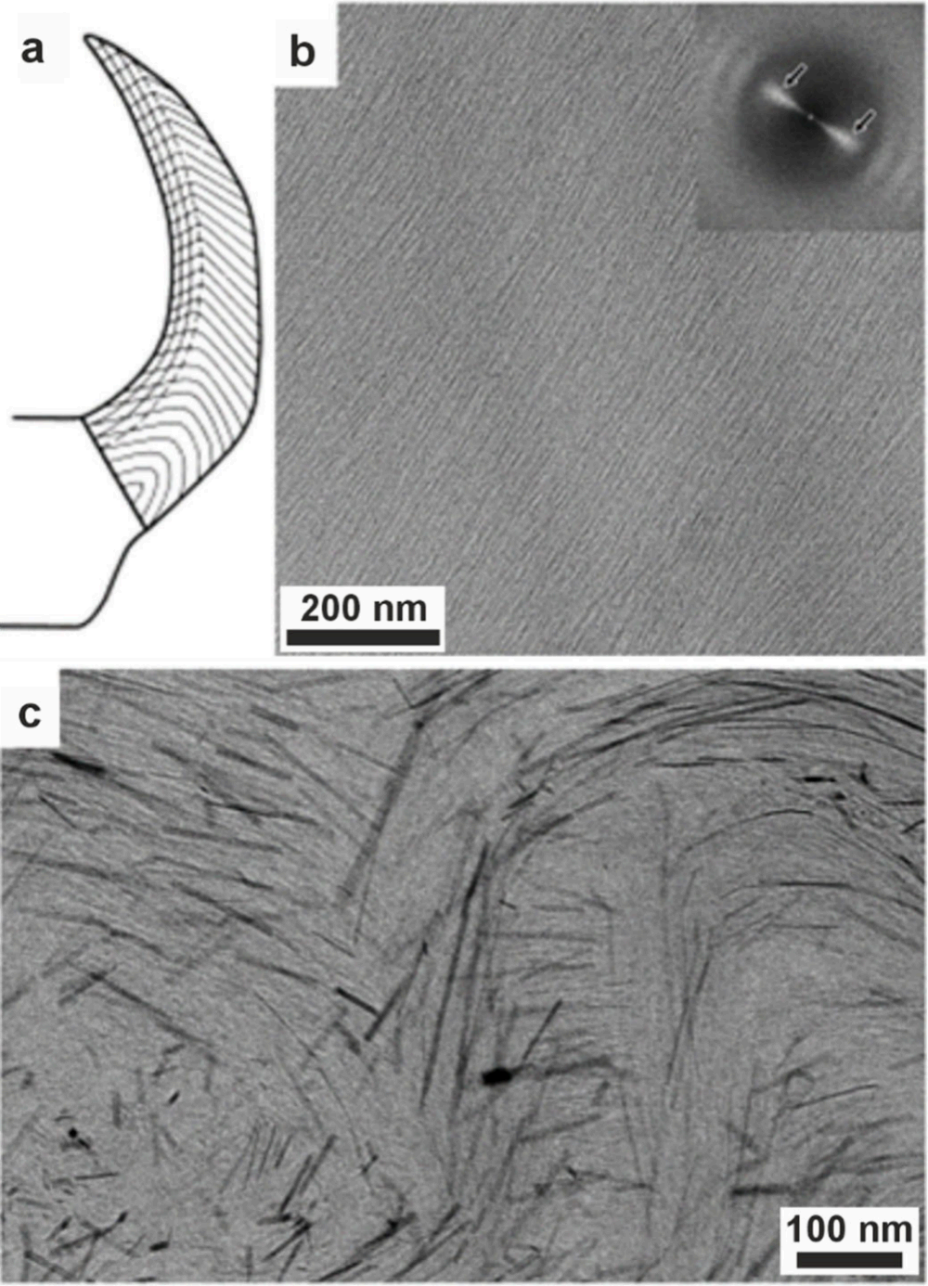
**Crystal orientation in limpet teeth.** (a) Sketch showing the orientations of the organic and mineral structures in longitudinal sections of mineralized limpet teeth. Reproduced with permission from van der Wal et al.^611^ Copyright 2000 Elsevier Science S.A., all rights reserved. Cryo-TEM image of cryo-sections of (b) unmineralized and (c) mineralized limpet teeth. Reproduced with permission from Sone et al.^612^ Copyright 2007 Elsevier Inc., all rights reserved. Inset in (b) shows a FFT of the micrograph, where the ≈7 nm periodicity of the dark lines is evident as a pair of diffuse spots (arrows).

The mechanisms underlying the co-orientation of the goethite crystals and chitin fibers have been investigated using cryo-TEM.^612^ First, an organic matrix of α-chitin that comprises oriented parallel arrays of chitin fibers with thicknesses of 6–9 nm and interfibrillar spacings of 2–5 nm is deposited (Figure 28b).^612^ Goethite crystals initially nucleate on the fibers and subsequently push the fibers apart as they grow into ≈4 × 4 × 100 nm needles that are aligned with the fibers (Figure 28c).^612^ These crystals grow within these confined, anisotropic environments within the fiber assembly, probably contributing to the formation of the extreme aspect ratios of the mature crystals.^607^ This could derive from the physical constraints of the organic matrix, and precursor species could potentially be introduced from specific directions to accentuate the acicular form.

### 5.4 Ascidian Sea Squirt Spicules with Oriented Vaterite

The sea squirt *Herdmania momus* is one of the few organisms that constructs its hard tissues from vaterite.^613^ Vaterite is a kinetic polymorph of calcium carbonate, and is commonly seen as a precursor to calcite or aragonite in synthetic systems, where it usually transforms rapidly to these more stable polymorphs in solution.^614^ *H. momus* forms vaterite spicules within its body and tunic (the tough, unmineralized exterior sac that protects the animal). The ≈100 μm tunic spicules form within compartments anchored to the inside wall of the tunic blood vessels, while the body spicules have high aspect ratios, reach lengths of 1–2 mm, and are composed of rows of acicular crystals that are closely packed to give crown-like structures.^615^ Although each individual crown was long considered a single crystal,^616^ an X-PEEM study using PIC mapping revealed that they are actually polycrystalline, such that the *c*-axes of the constituent crystals are coaligned by ±15°.^615^ TEM studies of demineralized spicules indicate that they are present within extracellular sheaths^617,618^ that may define the shape and size of the crystals and induce orientation. These studies used demineralized resin embedded sections, so it is not possible to see how the organic-mineral interface interact, as the inorganic vaterite was removed before imaging. Cryo-sectioning and high resolution imaging techniques may be better suited to determining how vaterite crystals nucleate and grow in these animals and to elucidate the mechanisms underlying the orientation. It is also expected that confinement effects contribute to stabilization of the vaterite, where the effect of confinement on polymorph is discussed in Section 6.

## 6 Controlling the Polymorph

One of the best demonstrations of the control exerted by organisms during biomineralization processes is their ability to produce specific polymorphs. This is beautifully exemplified by calcium carbonate biominerals, where calcium carbonate can form three anhydrous polymorphs—calcite, aragonite and vaterite—all of which can be precipitated from aqueous solution at room temperature. Of these, calcite is the most thermodynamically stable, aragonite is only slightly less so, and vaterite is a kinetic polymorph.^614^ Both calcite and aragonite are produced in enormous quantities as biominerals (Sections 3.2, 4.3 and 5.1), whereas there are only a few known vaterite biominerals (Section 5.4).^2^ Notably, it is difficult to synthesize aragonite in vitro at room temperature in the absence of additives. Magnesium ions are abundant in seawater^619^ and are effective in forming aragonite, where they inhibit the growth of calcite abiotically.^620^ By comparison, few synthetic organic additives have been identified that generate aragonite under ambient conditions in the absence of magnesium ions.^621–623^

Although polymorph control in biomineralization is usually attributed to the effects of specific organic additives and/or templates,^21,624^ it is likely that confinement also plays a role in polymorph control in many organisms. The ability of confinement to influence polymorph has been widely reported in synthetic systems for crystals including calcium carbonate,^98^ calcium sulfate,^96,113,118^ calcium phosphate,^114^ perovskites,^95^ glycine,^94^ ROY,^109^ and even crystals of colloidal particles.^625^ It is generally observed that metastable polymorphs are stabilized in confinement, but many questions remain about the origins of this behavior.^65^

Despite the multiple examples in synthetic systems, polymorph control through confinement in biomineralization has seldom been discussed, let alone proven. A significant factor is undoubtedly the challenge of studying the nucleation of biominerals in vivo. Whether formed from a liquid-like or an amorphous precursor, local dissolution–reprecipitation, or ion-by-ion addition to a crystal nucleus, the steps leading to polymorph control are poorly understood. Multiple mechanisms are also likely to combine to control polymorph, making it difficult to assess their relative contributions. Finally, only a few biominerals can form multiple metastable polymorphs, limiting the number of model systems that can be evaluated. In this highly speculative section, we focus on polymorphism in crystalline biominerals, where stabilization of amorphous phases in confined volumes is considered in Section 3.

### 6.1 Using Biomimicry to Study Polymorph Control in Mollusk Shells

Mollusk shell provides an exquisite demonstration of the ability of organisms to control crystal polymorph, where many switch between layers of calcite and aragonite with perfect fidelity. This activity has long been attributed to the action of specific biomolecules, where multiple studies have attempted to identify those that can select for calcite or aragonite.^57,59,60^ A notable success is the work of Belcher et al., who were able to switch between the growth of calcite and aragonite in bulk solution by adding biomolecules extracted from the calcitic and aragonitic layers of the abalone *H. rufescens* (Figure 23b).^60^ However, the scarcity of successful results suggests that the mechanism operating in organisms is not as simple, where the soluble biomolecules extracted from aragonite biominerals have generally only formed aragonite when combined with an insoluble organic matrix. The acidic matrix Pif proteins only promoted the formation of aragonite crystals when confined between a chitin membrane and glass slide (Figures 23c, d).^59^ A more complex nacre mimic made from β-chitin, silk fibroin, and macromolecules extracted from the aragonitic or calcitic layers of the mollusks *Atrina rigida* and *Mytilus californianus* formed aragonite in the presence of the aragonitic proteins, and calcite with the calcitic proteins.^57^

Further mimicking biological environments, aragonite has been formed within organic matrices in the presence of polyelectrolytes, including reacetylated chitosan thin films containing pAA^626^ and pVA matrices with pAA.^627,628^ More general control over calcium carbonate polymorph has also been achieved in cross-linked, non-cross-linked, and uniaxially deformed cross-linked gelatin films infiltrated with poly-l-aspartate and poly-l-glutamate.^629–632^ The degree of deformation of the gelatin films, and the type and concentration of the polyelectrolyte influenced the crystal polymorph and orientation, where a general sequential precipitation of calcite, aragonite, and finally vaterite was recorded with increasing concentration of polypeptide. Higher polypeptide concentrations would be expected to increase the concentration of calcium ions, and thus the supersaturation within the gelatin matrices, favoring the production of metastable polymorphs. Uniaxial orientation of the gelatin also influenced polymorph, suggesting that a potential link between a reduction in the cavity size within the ECM mimicking gel and the supersaturation of the solution.^629,632^

Aragonite has also been formed in confined volumes in the absence of soluble organic additives or gel-like environments. Calcium carbonate was precipitated within the pores of TE membranes in the presence of magnesium and sulfate ions, where reaction conditions were selected that yielded only 7% of the crystals as aragonite in bulk solution (Figure 29).^98^ This fraction increased to 19% in 800 nm diameter pores, 69% in 200 nm pores, and reached 100% aragonite in 50 and 25 nm pores. Notably, aragonite was the only polymorph produced in 25 nm pores when crystallization was carried out in the absence of any additives (Figure 29c). Analysis of the relationship between the proportion of aragonite formed in the membrane pores and the inverse of the pore diameter revealed a roughly linear relationship, which is expected if the number of aragonite nucleation sites is proportional to the surface area (*d* ^2^), the quantity of calcium carbonate produced is proportional to volume (*d* ^3^), and the crystal growth rate is constant. This suggests that control of the formation of aragonite in this system is dominated by the surface of the pores.

**Figure 29:**
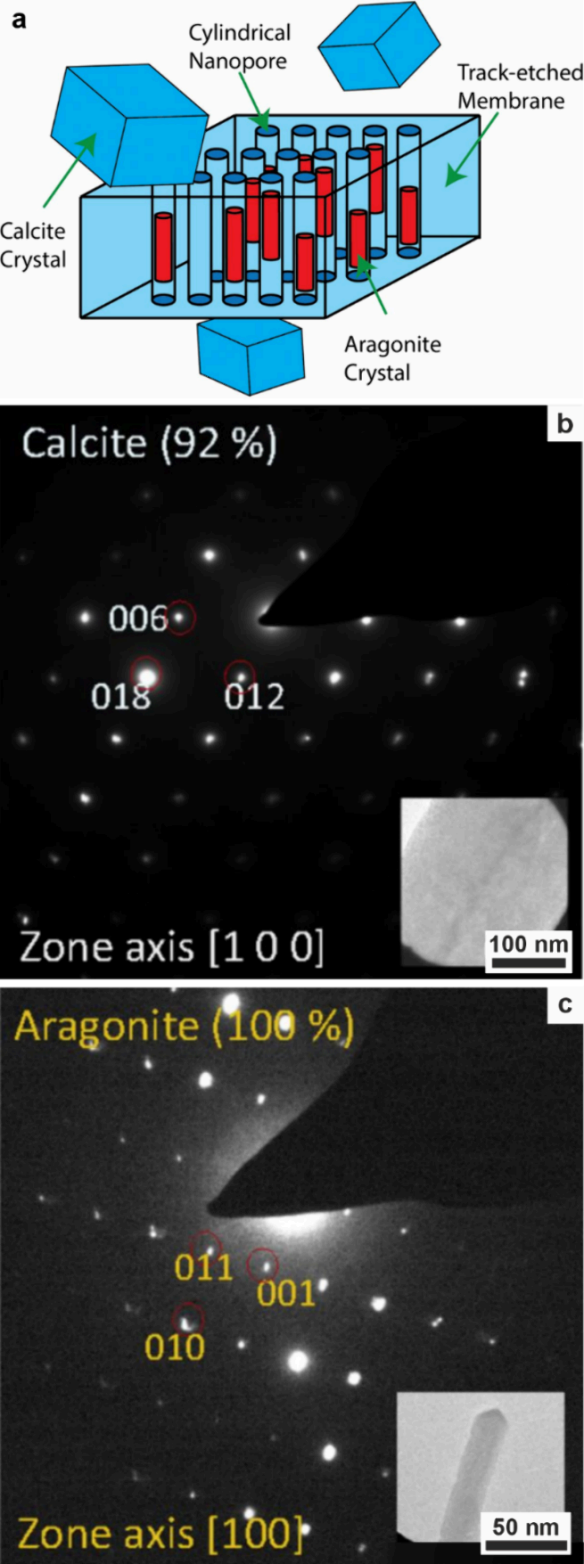
**Aragonite formation in nanoporous TE membranes.** (a) Schematic diagram of calcium carbonate crystallization within the cylindrical nanopores of TE membranes. Reproduced with permission from Xu and Sommerdijk^633^ Copyright 2018 National Academy of Science under the PNAS license. (b, c) SAED and TEM images of CaCO_3_ formed in TE membranes. Reproduced with permission from Zeng et al.^98^ Copyright 2018 National Academy of Science under the PNAS license. SAED and inset TEM of crystals precipitated within (b) 200 nm and (c) 25 nm membrane pores using [Ca^2+^] = [CO_3_^2–^] = 1.5 mM. The polymorph and relative abundance are also shown.

These data therefore show that physical environments can play important roles in controlling polymorph in synthetic environments, suggesting that similar effects could occur in biological systems.

### 6.2 Bassanite Statoliths in Jellyfish

Calcium sulfate has two crystalline hydrated forms, where bassanite (CaSO_4_·0.5H_2_O) is metastable in aqueous solution and can act as precursor to the stable form gypsum (CaSO_4_·2H_2_O).^96,113,634–636^ Calcium sulfate biominerals are rare in nature, being found in the stems of the plants *Salvadora persica* ^637^ and *Tamarix aphylla* ^638^ and within the Scyphozoan jellyfish.^639,640^ The latter forms calcium sulfate particles within statocysts (gravity-sensing organs) (Figure 30a).^640^ These comprise micrometer-sized disc or rod shaped bassanite crystals (Figures 30b–d) that are enclosed by a lipid and protein bound vesicle.^639–641^ That bassanite has a higher density than gypsum and is thus better-suited to use within gravity or inertia sensing organs, suggests a possible evolutionary driver for selection of this mineral.

**Figure 30:**
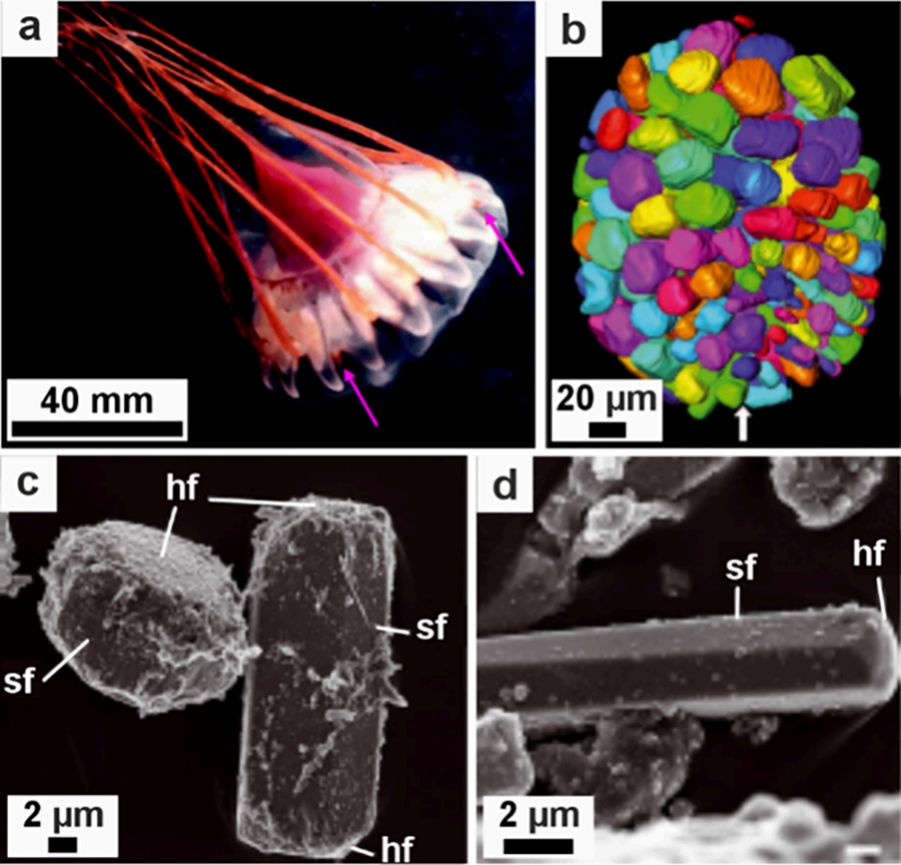
**Statoliths and statocysts in jellyfish.** (a) A medusa of *Periphylla periphylla* with statocysts indicated by arrows. Reproduced with permission from Tiemann et al.^640^ Copyright 2002 The Royal Society of Chemistry. (b) Statocyst and (c, d) individual statolith crystals. Reproduced with permission from Heins et al.^10^ Copyright 2018 The Author(s) and Inter-Research with CC-BY license. (b) X-ray tomographic reconstructions of statocysts from 42 day old *Sanderia malayensis* medusa, with colors indicating the separate statoliths. (c, d) SEM images of individual statoliths from 42 day old *S. malayensis* medusa showing (c) a disc (left) and (right) rod and (d) a longer rod. Labels: head face, *hf*; side face, *sf*. Organic matter clinging to surface of the particles creates a lumpy appearance.

Metastable bassanite and amorphous phases have been detected as precursors to gypsum in aqueous solution,^96,634–636^ and large quantities of bassanite can be formed by quenching aqueous calcium sulfate solutions with >70% ethanol.^642^ Polyelectrolytes including poly­(acrylic acid) and poly­(styrene-4-sulfonate), and the simple inorganics sodium triphosphate and magnesium chloride are also able to stabilize bassanite against transformation to gypsum for 1 to 2 days.^636^ Confinement has been shown to stabilize amorphous calcium sulfate and bassanite in vitro using a crossed cylinder apparatus,^113^ TE membrane pores,^118^ and controlled pore glass rods.^96^ These systems, which were open to a reservoir of mineralization solution, were able to stabilize bassanite for months in open pores of diameters <100 nm. Notably, the bassanite rapidly transformed to gypsum when the confinement was removed^118^ and synthetic dry bassanite rapidly transforms to gypsum on addition of water.^636,643^

Confinement effects are therefore likely to contribute to the stabilization of bassanite within the statocysts. The biomineralized bassanite statoliths transform to gypsum when washed with water^639^ or even dissolve when the thin protective layer of organic matter on their surface is removed.^640^ The lipid and protein layer that coats the bassanite isolates it from external fluids and thus stabilizes it against transformation to gypsum, which typically proceeds by a dissolution/reprecipitation mechanism.^118,635,643–646^ The presence of additional biomolecules and inorganic ions may enhance this stabilization effect, but these have not yet been conclusively identified.

### 6.3 Selecting the β-Guanine Polymorph In Vivo and In Vitro

Guanine is an organic crystal that is employed in dinoflagellate algae, fish, chameleons, crustaceans, spiders, beetles, bacteria, and mollusks for both its photonic properties and its ability to act as a nitrogen store^647–656^ and is thoroughly reviewed elsewhere.^657,658^ The extremely high refractive index (*n* = 1.83) of guanine crystals^659^ make them ideally suited to roles in which optical properties are desired such as multilayer broadband^660–662^ and narrowband reflectors.^663^ These crystals are usually in the form of platelets (Figure 31a) and are confined by cytoplasmic layers (Figures 31b–e), while crystals with less defined shapes have been detected in vacuoles in dinoflagellate cells (Figure 31d, e). Guanine is highly insoluble in aqueous solution at neutral pH, such that lab syntheses are typically carried out in organic solvents,^664^ or in aqueous solution at high or low pH, where the solubility is much greater.^665^

**Figure 31:**
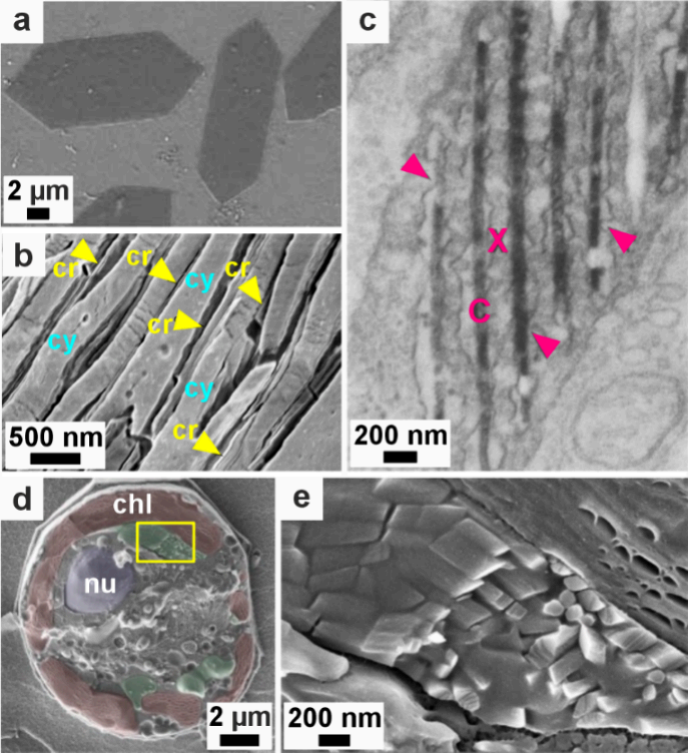
**Guanine biominerals.** (a) SEM image of guanine crystals extracted from the skin of a koi fish and (b) cryo-SEM image of an iridophore cell. Reproduced with permission Levy-Lior et al.^666^ Copyright 2010 WILEY-VCH Verlag GmbH & Co. KGaA, Weinheim. Panel (b) shows the regular layered organization of the crystals (labeled *cr*) as well as the cytoplasmic layers (labeled *cy*) between the crystals. (c) TEM image of an ultramicrotome section of an iridophore of *Poecilia reticulata*. Reproduced with permission from Gundersen and Rivera^650^ Copyright 1982 Wiley-Liss, Inc. This shows guanine platelets (labeled *C*) and the cytoplasm layers in between (labeled *X*). (d, e) Cryo-SEM micrographs of guanine crystal containing vacuole in a *Calciodinellum operosum aff*. cell at (d) low and (e) high magnification. Reproduced with permission from Jantschke et al.^649^ Copyright 2019 Elsevier Inc., all rights reserved. (d) Photosynthetic dinoflagellate cell, showing chloroplasts false colored in pink and the nucleus in blue. (e) High magnification area of guanine (colored green in (d)) from the yellow box.

There are three known forms of guanine—monohydrate,^667^ anhydrous α-guanine (α-AG)^668^ and anhydrous β-guanine (β-AG)^669^—but only β-AG has been found in organisms so far.^659^ As both α-AG and β-AG have the same high refractive index, and their crystal structures and physical properties are extremely similar, this suggests that β-AG is easier to form and/or stabilize in vivo. α-AG is more thermodynamically stable than β-AG under ambient conditions,^669^ and β-AG quickly converts to α-AG in the presence of water.^665^ β-AG is also challenging to produce in synthetic systems but has been precipitated from organic solvents such as dimethyl sulfoxide,^664,665^ and a mixture of α-AG and β-AG is precipitated from aqueous solution at pH 13.^665^

An amorphous precursor to guanine has been observed in synthetic systems, where rapid neutralization of acidified guanine in vitro leads to precipitation of an amorphous phase, and additives can be used to direct the polymorph of crystals formed from amorphous guanine.^664,670^ For example, α-AG microplates were made when adenine or guanosine was added to a guanine-formamide solution, and β-AG plates when uric acid, hypoxanthine or xanthine were added.^670^ β-AG can also be formed by hydration of an amorphous guanine using water vapor.^671^ A recent in vitro study also investigated the precipitation of guanine from aqueous solution within the confines of water–oil–water double emulsions, where a low pH solution of guanine was encapsulated and precipitation was induced within the emulsion cores by increasing the pH of the exterior solution.^672^ Control over the polymorph was achieved in these environments according to the reaction conditions, where spherical β-AG crystals formed if high initial guanine concentrations were employed, while needles of monohydrate formed at low guanine concentrations.

To bypass the issues with the low solubility of guanine at physiological pH, a recent study employed an enzyme (purine nucleoside phosphorylase) to convert the more soluble guanosine precursor to guanine in phosphate buffer at pH 7.2.^673^ β-AG ribbons were produced when the enzyme was able to increase the supersaturation of guanine rapidly, while the stable α-AG polymorph formed at low crystallization rates. Additives including purines, dyes, amino acids, and polymers modified the morphology and twinning frequency of the crystals but not the polymorph formed.^673^ These data indicate that the rate at which an organism increases the supersaturation of guanine may be key to generating the β-AG crystals, so rapid influx or generation of guanine within a confining matrix would be key for this mechanism to apply.

Nitrogen-starved dinoflagellate microalgae *Amphidinium carterae* take up guanine from their surroundings^648^ and store this intracellularly as crystalline guanine within vesicles (Figure 32a). Raman spectroscopy of another dinoflagellate *Calciodinellum operosum aff*. identified the β-AG polymorph within vesicles closely associated with the chloroplasts in the cells, which proposed that these could help collect light and/or offer protection to the photosynthetic organelles.^649^ 1–2 μm vesicles filled with a material consistent with amorphous guanine have also been seen in the spiders *Latrodectus pallidus* ^666^ (Figure 32b, c) and *Phoroncidia rubroargentea*.^674^ There is strong evidence that guanine fish scales form via an amorphous precursor, where stacks of guanine crystals and vesicles filled with amorphous guanine were observed in the koi *Cyprinus carpio* (Figure 32d, e).^675,676^ Similar vesicles were observed in the guppy *Poecilia reticulata*,^650^ the zebrafish *Danio rerio*,^677^ and the neon tetra *Paracheirodon innesi*.^678^ That the guanine crystals present in fish and spiders do not have nanogranular textures^150,679^ suggests that crystallization does not follow a solid-state transformation from the amorphous precursor (e.g., Section 3.3); the vesicles simply store amorphous guanine, and crystallization likely occurs by a dissolution/reprecipitation mechanism.

**Figure 32:**
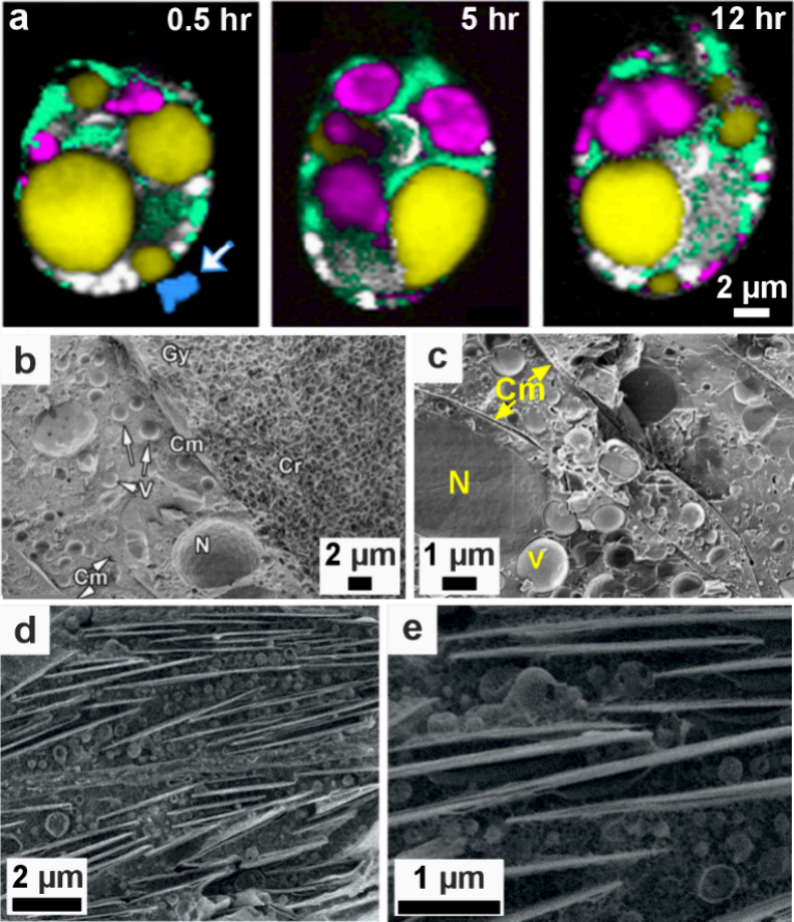
**Membrane-bound guanine structures.** (a) Raman microscopy images of nitrogen-starved *Amphidinium carterae* at different time points after feeding with crystalline deuterated guanine (blue, indicated with arrow). Reproduced with permission from Mojzeš et al.^648^ Copyright 2020 National Academy of Science under the PNAS license. The organism recrystallized the guanine inside vesicles (magenta), with chloroplasts (green), starch (white), and lipids (yellow) also shown. (b, c) Cryo-SEM images of freeze fractured white-colored tissues from the spider *Latrodectus pallidus*. Reproduced with permission Levy-Lior et al.^666^ Copyright 2010 WILEY-VCH Verlag GmbH and Co. KGaA, Weinheim. Labels: guanocytes, *Gy*; filled with guanosine crystals; *Cr*; cell membrane, *Cm*; nucleus, *N*; and vesicle, *V* filled with solid material. (b) The cell (left) adjacent to crystal rich area (right) is packed with solid filled vesicles. (c) Another cell adjacent to a crystal-rich area, also packed with solid filled vesicles. (d, e) Cryo-SEM images of freeze-fractured silvery koi *Cyprinus carpio* scales. Reproduced with permission from Gur et al.^675^ Copyright 2014 American Chemical Society. These show cross sections through crystal stacks and the multivesicular cytoplasm in between the guanine crystals.

These observations indicate that confinement plays a significant role in the mineralization of guanine in vivo. Vesicles are most abundant adjacent to the most recently formed layer of guanine crystals in fish scales,^676^ suggesting that they are responsible for solubilizing guanine and delivering it to the site of mineralization. They may allow the organism to control the pH, thereby adjusting solubility, and the colocation of additional molecules may promote the formation of amorphous guanine and direct the polymorph of the product crystals. Confinement is also expected to stabilize this amorphous phase, as is widely observed for crystallization in small volumes, and may facilitate the formation of the β-AG crystals, where the close proximity of an organic matrix will limit their contact with water^181^ and inhibit transformation to α-AG.^659^ Indeed, β-AG crystals in dinoflagellates rapidly transformed to α-AG when the organic confinement was removed,^649^ demonstrating its role in stabilizing the β-AG phase.

## 7 Nucleation and Nanoreactors

Underpinned by molecular-scale, dynamic, and often rare events, the characterization of nucleation processes remains one of the most challenging topics in the field of crystallization. While it is extremely difficult to study nucleation in synthetic systems, determining how organisms control the nucleation of biominerals offers an order of magnitude greater difficulty, where this would ideally be studied in situ. Our final section addresses this topic by highlighting several systems that have given us insight into the early stages of biomineralization. These involve well-defined, nanoscale reaction environments that are accessible for detailed characterization.

Nanoscale confinement is widely used in biology to control reactions, where it can reduce the lifetime and negative impact of toxic intermediates and increase reaction rates by creating high local concentrations of precursors and reducing the distances between sequential reaction centers in cascades. There are also some examples where it influences biomineralization. This can be direct, where mineralization occurs within a nanoscale compartment such as in the iron storage protein ferritin, or indirect, such as when confinement promotes the formation of reactants involved in crystallization. An example of the latter is provided by the carboxysome, which is a self-assembled protein nanostructure that organizes the machinery that carries out CO_2_ fixation.^680–684^ The carboxysome colocalizes two enzymes, namely carbonic anhydrase, which catalyzes the hydration of CO_2_ to bicarbonate ions for carbonate biomineralization in some organisms,^236,685^ and RuBisCO, which fixes CO_2_ from the atmosphere to form organic matter. The efficiency of RuBisCO is significantly increased by increasing the local concentration of CO_2_, such that the carboxysome nanoreactor makes carbon fixation from CO_2_ and thus primary productivity possible.^682,686^

Examples that start simply then build in complexity are presented here. The first considers the ability of some organisms to selectively generate biominerals from low abundancy cations. This can be attributed to the organism using confinement to control the supersaturation and the presence of crystallization inhibitors. The nucleation of HAp within the gap regions of collagen fibrils then provides a contrasting example, where this system provides insight into how nucleation occurs within the fibrils. The iron storage protein ferritin, which is among the most beautiful and best-understood of all biomineralization systems, is considered next. Due to its relative simplicity, it is accessible to techniques including cryo-TEM, XRD, NMR and site-directed mutagenesis. Analysis of ferritin mineralization using these techniques has identified the protein residues involved in the formation of the mineral core and has shown how the confines of the protein shell localizes high concentrations of precursor ions, controls redox processes, promotes nucleation, and stabilizes metastable polymorphs. Finally, we consider magnetite production by magnetotactic bacteria. Although a far more complex system than ferritin, sequencing of the genetic code of a number of strains has provided exceptional insight into biomineralization control strategies in these organisms and revealed the roles that various proteins play in generating magnetite crystals within the magnetosome.

### 7.1 Formation of Ba and Sr Sulfates for Gravity Perception

A few organisms have evolved to generate biominerals from low-abundance cations. For example, barium sulfate (BaSO_4_, barite) and strontium sulfate (SrSO_4_, celestite) are made by some desmids^687,688^ and charophytes for use in sensing gravity,^689,690^ while *Radiolaria* ^691^ and dinoflagellates with symbiotic relationships with *Acantharia* ^692–694^ can selectively deposit celestite spines and skeletons (cysts) for protection.^690^ Given that calcium is far more abundant than strontium or barium in the ocean (400 ppm as compared with 13 and 0.05 ppm, respectively^695^), these organisms must have developed strategies to concentrate and generate minerals from the less abundant cations. For example, the diplonemid *Namystynia karyoxenos* accumulates intracellular membrane-bound crystalline deposits of Ba^2+^ and Sr^2+^ at concentrations 42,000× and 10,000× higher than those found in the surrounding medium.^696^

We here focus on statolith mineralization within the desmid *Closterium moniliferum*, as it is likely that this process is controlled by compartmentalization combined with a suite of proteins. Most ion selective processes in organisms are facilitated by selective ion pumping across biological membranes.^697,698^ However, this may not be the origin of selectivity between Ca^2+^, Sr^2+^, and Ba^2+^, as most of the proteins involved in binding and transport of cations are not able to differentiate between alkaline earth metals.^699^ In mammals, calcium can be selectively and actively transported across cell membranes over strontium, but this is not common.^700^ As these studies are ≈50 years old, and based on animal rather than plant transporters,^701^ unravelling the mechanisms employed to selectively concentrate barium or strontium for biomineralization requires scrutiny using modern techniques.

It has been suggested that “sulfate traps” may operate to control the selective formation of barite and celestite in biomineral deposition vesicles.^687^ In the sulfate trap model, calcium, strontium, and barium ions are transported by transmembrane proteins into vacuoles in roughly the same ratio as found in seawater (with a slight bias against Ca^2+^) and can accumulate at higher concentrations than in seawater.^687^ This hypothesis has been supported by X-ray fluorescence microscopy (XFM) maps of the localization of Ca, Sr, and Ba within *C. moniliferum* grown in freshwater media containing 17 mM Ca^2+^ supplemented with, but undersaturated with respect to Ba^2+^ and Sr^2+^.^687^ XFM maps showed that all three elements (Ca, Ba and Sr) were elevated above background levels between the lobes of the chloroplast and at the center of the cell, and that strontium and barium were also concentrated in the terminal vacuoles^687^ (Figure 33b). These areas of high concentrations correlate with the location of nanoscale vesicles within the organism (Figure 33c–e).^687,702^

**Figure 33:**
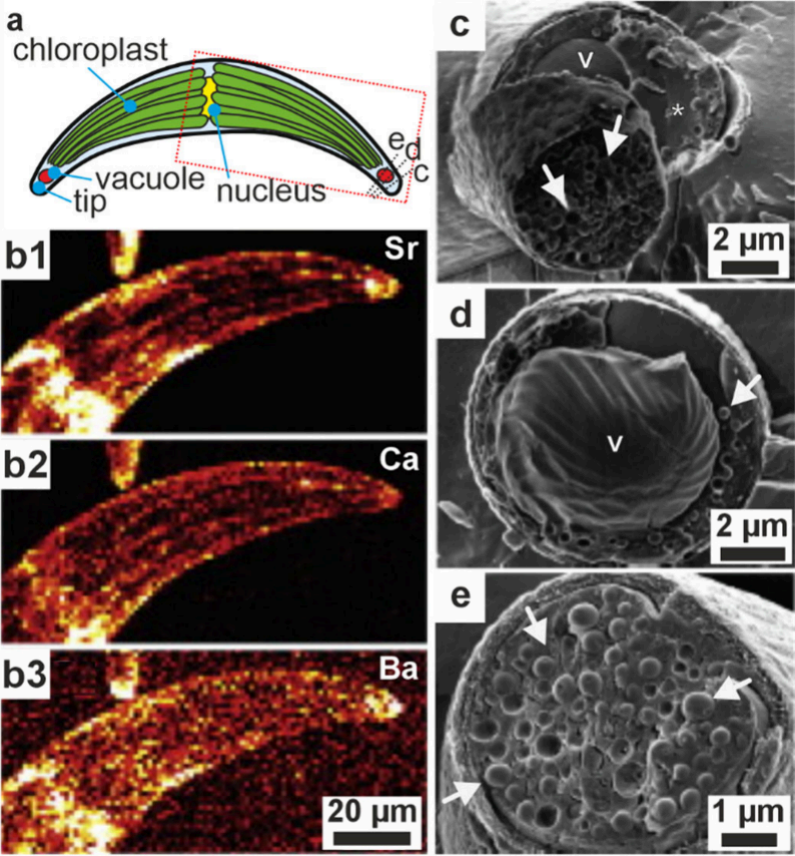
**Freeze fracture cryo-SEM and XFM maps of** ***Closterium moniliferum***. (a) Redrawn and (b–e) reproduced with permission from Krejci et al.^687^ Copyright 2011 Elsevier Inc., all rights reserved. (a) Sketch of section through *C. moniliferum* labeled with fracture sections and features of interest. XFM maps showing (b1) strontium concentrations were highest between the chloroplasts, in the terminal vacuole, and in the center of the cell; (b2) calcium distribution was similar to strontium but not elevated in the terminal vacuole; and (b3) barium detection was increased from the terminal vacuole and center of the cell. Differences in signal-to-noise are due to the detection limits. (c–e) Freeze fractured transverse sections (areas annotated on (a)) from near the cell tip showing the terminal vacuole and a vesicle dense region. Annotations: vacuole, *v*; vesicles, marked with arrows; and vesicle poor regions, marked *.

Simple bulk thermodynamic calculations (e.g., in Visual MINTEQ ver. 3.1,^703^ using Ca^2+^, Sr^2+^, Ba^2+^, and SO_4_^2–^) show that, for low cation concentrations (0.1–0.5× seawater abundance of Ca:Sr:Ba 40:1.3:0.05 mM^695^), and high sulfate concentrations (1–4× that found in seawater, 28–112 mM^695^), a solution is solely oversaturated with respect to barite. At intermediate ion concentrations, barite and celestite are oversaturated, and gypsum is undersaturated. At high concentrations of these cations (4×) and sulfate (2–4×), a bulk solution is oversaturated with respect to barite, celestite, and gypsum. There is no combination of these conditions that is oversaturated solely with respect to celestite in the presence of Ca^2+^, Sr^2+^, Ba^2+^ and SO_4_^2–^. These factors may explain why diplonemid protist biominerals consist of celestite with traces of barite,^696^ as the sulfate trap may maintain undersaturation with respect to gypsum but be oversaturated for celestite and barite. In order to form barium-free strontium sulfates, organisms must be able to omit barium from any calcium and strontium containing biomineralization vesicle, use additives to favor celestite or discriminate against barite/gypsum, or the confinement condition is able to select for the celestite phase.^179,704,705^

If alkaline earth cation transport machinery is able to actively select for calcium, strontium, or barium, these could be used to fill a vesicle with just strontium or just barium.^687^ Alternatively, nonselective pumps could transport Ca^2+^, Sr^2+^, and Ba^2+^ from seawater into a confined locale, then selective transporters could remove the calcium or barium, enriching the interior in Sr^2+^ and precipitating celestite in the presence of sulfate. Both scenarios are consistent with the XFM maps shown in Figure 33b, where Ca, Sr, and Ba are all enriched in the cell cytoplasm surrounding the chloroplasts, but only Sr and Ba are concentrated in the vacuoles at the cell tips.

For further evidence, we turn to organisms that form amorphous alkaline earth carbonates. Amorphous carbonates formed abiotically contain cations at the same ratio as the solution from which they precipitated and so do not preferentially form one particular alkaline earth carbonate mineral over another,^179^ nor do they form zoned deposits when cations are added sequentially.^704^ However, biomineralized amorphous carbonates do show selectivity, and can form single phase and zoned carbonates that contain a different cation ratio to that found in the surrounding solution.^705^ The alga *Bryopsis maxima* shows elevated Sr/Ca and Ba/Ca ratios as compared to seawater,^706^ and a carbonate-accumulating cyanobacterium *Gloeomargarita lithophora* showed evidence of molecular machinery that selectively enriches the heavier alkaline earth metals,^706,707^ indicating that heavy ion selectivity in the accumulation of alkaline earth elements in plants is possible.

Fractionation of alkaline earth metals does not occur abiotically in carbonates,^179,707^ but it does in sulfates. This means that the trap mechanism would be specific to sulfate mineralization, and the organisms that preferentially form strontium or barium carbonates, like *G. lithophora*, use selective alkaline earth ion pumps, or some other cation or mineral selection mechanism. Unravelling these processes requires genetic studies to identify the biomolecules involved in ion transport and biomineral formation, as has been done for diatoms (Section 3.6.1) and magnetosomes (Sections 4.2.1 and 7.4).

### 7.2 Calcium Phosphate Nucleation within Collagen Fibrils

Bone is a hierarchically organized composite material constructed from type-I collagen and carbonated HAp (Sections 3.5 and 5.2.1). Here, we discuss how HAp nucleates and grows within collagen fibrils. In bone, HAp nucleation occurs within the intermolecular channels in the 2 × 2–4 × 20–36 nm gap regions found in assembled collagen fibrils.^581,708–711^ It has been proposed that the nucleation of HAp in these sites is promoted by charged amino acids present within the gap region, which are able to bind calcium and phosphate ions^383,577,708,712–714^ and cause a localized increase in supersaturation.^193^ Modeling and AFM studies also suggest that the density of water is lower within the gap region, which would facilitate ion desolvation and thus mineral nucleation.^553,712,714,715^ The preferential nucleation of HAp crystals within the gap region creates a staggered arrangement of the HAp platelets and contributes to the characteristic banding pattern observed.^716,717^

Notably, it is difficult to achieve HAp mineralization within collagen fibrils in vitro, which can be attributed to the very small volumes in which the crystals nucleate.^718^ Ions present within the fibrils would be rapidly consumed when a crystal nucleates, and their growth limited by the rate of ion diffusion into the collagen.^710,719^ Therefore, when unmineralized collagen is incubated in a supersaturated calcium phosphate solution, mineral solely forms on the exterior of the fibrils.^193^ Intrafibrillar mineralization only occurs when mineralization in the exterior bulk solution is inhibited, for example by adding polyelectrolytes.^184^ These inhibitors are too large to enter the intrafibrillar spaces and so are excluded,^194,279^ leading to preferential mineral deposition within the collagen structure (see Section 3.5). This inhibition strategy has also been used to achieve intrafibrillar mineralization of noncollagenous fibers such as cellulose,^720^ polypeptides,^721^ and block copolymers.^722^

Notably, there are some key differences when collagen is mineralized in vivo as compared with in vitro. When collagen fibrils are mineralized in vitro they are usually highly mineralized in both the gap and overlap regions,^184,193^ whereas those mineralized in vivo are usually only heavily mineralized in the gap regions.^723^ The origin of this difference is unclear, but it may be that researchers are not quite capturing the complexity of the natural bone mineralization environment in order to achieve true biomimicry of mineral localization to the gap regions. The staggered arrangement of the HAp crystals and higher mineral density in the gap regions can be generated in vitro by adding phosphorylated noncollagenous proteins to the mineralization solution,^724,725^ by cross-linking assembled collagen fibrils to polyvinyl phosphonic acid to phosphorylate the primary amines,^724^ or by mineralizing the collagen fibrils during their self-assembly in the presence of pAA (molecular weight 2 kDa).^726^ The first example may be the closest mimic of the in vivo scenario, as noncollagenous proteins involved in bone mineralization processes (such as osteopontin) are also phosphorylated.^727^ The detailed mechanisms underpinning the formation of the staggered HAp arrangement still needs to be determined.

It has also been widely debated whether ACP or calcium phosphate PILP acts as a precursor to the formation of HAP crystals in collagen.^184^ While ACP has been observed during in vivo bone mineralization, the presence of PILP has not yet been confirmed (see Sections 3.2 and 3.5).^253^ In vitro experiments indicate that soluble polymers can both stabilize ACP and facilitate intrafibrillar HAp formation^728^ but as yet there is no evidence that the biomolecules involved in bone mineralization facilitate the process by stabilizing ACP or PILP. Very recently, one such biomolecule called poly­(adenosine diphosphate-ribose) (PAR) was found to bind strongly to the C-terminal telopeptides of type I collagen.^729^ In vitro mineralization experiments have also shown that PAR binds with calcium ions to form droplets, and that this complex can bind to collagen fibrils (probably at the telopeptide sequences) to deliver calcium ions to the gap regions, leading to HAp formation.^729^ No ACP phase was directly observed during this study, although the calcium-PAR droplets share some similarity with PILP phases.

Assuming that PILP or ACP clusters act as precursors to intrafibrillar HAp crystals, they first need to infiltrate the fibril. This could be driven by capillary forces,^728^ electrostatic forces,^193^ or a balance between osmotic and electrostatic forces (the Gibbs–Donnan equilibrium).^194^ Due to the challenges associated with characterizing in vivo mechanisms, these infiltration and transformation processes have not yet been observed in vivo. However, ACP infiltration into collagen fibrils has been unambiguously observed in 3D by cryo-ET in an in vitro mineralization experiment in the presence of copper ions from the TEM grid.^728^ Unfortunately no intrafibrillar HAp platelets were formed, as the collagen mineralization was disrupted by the presence copper ions. Recently, stochastic optical reconstruction also showed that ACP was able to infiltrate collagen fibrils when they were immersed in dimethyl sulfoxide containing 1 mg mL^–1^ of pAA-stabilized ACP nanoparticles.^730^ The ACP then transformed into HAp platelets when the fibrils were transferred to an aqueous buffer containing 2 mg mL^–1^ of pAA-stabilized ACP. A comprehensive understanding of the mechanisms underlying the mineralization of collagen is therefore yet to be achieved, as so far non-natural additives have been required to observe amorphous precursor infiltration to the fibrils.

### 7.3 Ferritins: Proteinaceous Nanoreactors

Ferritin is an iron storage protein that is found across almost all forms of life, including mammals, plants, and bacteria^731–734^ and has evolved to store iron in a nontoxic, bioavailable form.^735,736^ Comprising a roughly spherical, hollow shell that contains a poorly ordered phosphate and ferrihydrite (5Fe_2_O_3_·9H_2_O) mineral core,^737^ mineralized cages are referred to as ferritin or holoferritin, and empty proteins as apoferritin (Figure 34). The typical apoferritin molecule is constructed from 24 subunits, each of which comprises a 4-helix bundle, and the 3D form of ferritin is highly conserved in eukaryotes and prokaryotes, despite significant variation in the amino acid sequence.^738^ Ferritins comprising 12 subunits are also found in prokaryotes.^738,739^ Ferritin is highly efficient in promoting the formation of ferrihydrite, where in vitro incubation of apoferritin in the presence of Fe­(II) at neutral pH under oxidative conditions leads to exclusive mineral formation within the protein.^740^

**Figure 34:**
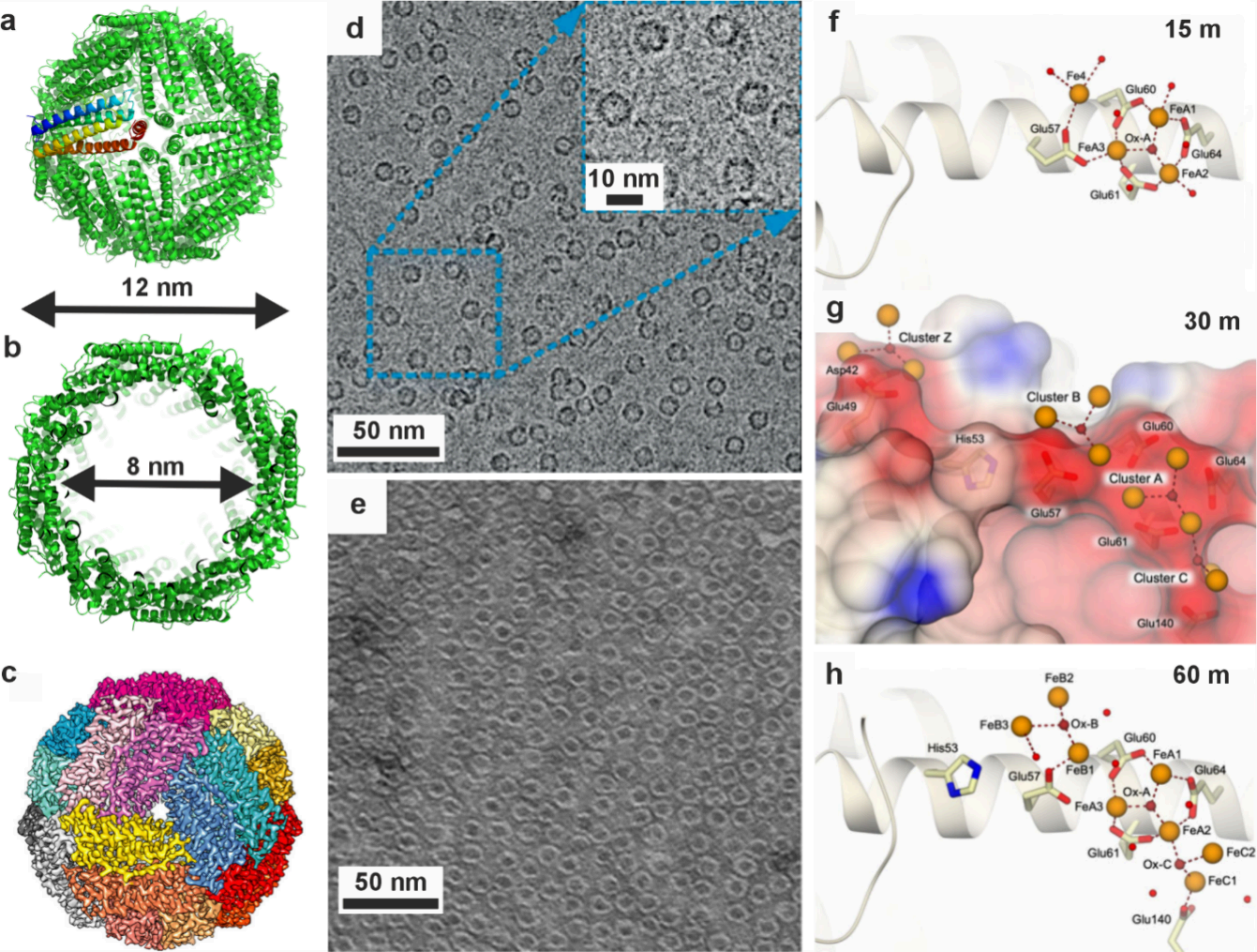
**Apoferritin and ferritin: X-ray and cryo-TEM structures and cryo and negative-stain TEM images.** (a) Exterior and (b) section view of the human L-chain ferritin structure showing ≈8 nm internal lumen (exterior diameter 12 nm), produced in PyMOL^29^ from the PDB 2FFX X-ray crystal structure.^30^ (c) 3.0 Å reconstruction from the (d) cryo-TEM image of equine spleen apoferritin. Reproduced with permission from Feng et al.^741^ Copyright 2017 Elsevier Ltd. Panel (c) shows the external surface viewed along one of the eight 3-fold axes. (e) Negatively stained TEM image of ferritin from *Pseudomonas aeruginosa*, where internal mineralized core appears dark. Reproduced with permission from Dehner et al.^742^ Copyright 2013 SBIC. (f–h) Snapshots of the Fe^3+^ binding to the inner surface of recombinant human L-chain ferritin. Reproduced with permission from Ciambellotti et al.^743^ Copyright 2020 Wiley-VCH Verlag GmbH & Co. KGaA, Weinheim. For these structures, Fe^3+^ was diffused into apoferritin protein crystals, and structures were solved to 2.1–2.3 Å. Binding of iron observed after (f) 15 min (PDB 6TSJ), (g) 30 min (PDB 6TSA), and (h) 60 min (PDB 6TSF) of Fe^3+^ exposure. (g) Electrostatic surface representation calculated from the X-ray structure of after 30 min Fe^3+^ exposure (red negative and blue positive charge) shows iron ions favor binding at the negatively charged regions of the protein surface.

Focusing on the best-studied animal 24-mer ferritin, it typically has outer and inner diameters of 12 and 8 nm, respectively, and the core can contain up to ≈4500 iron atoms that can be rapidly accessed by the organism as required.^733^ The confinement offered by the protein shell may contribute to the formation of ferrihydrite, which is poorly crystalline and a known precursor to hematite and goethite.^744^ It may also stabilize ferrihydrite against transformation to more crystalline iron minerals, as has been observed in other confined systems, such as within a chitosan network,^745^ mesoporous chrysotile asbestos^746^ and mesoporous silica.^747^

The 24 subunits are organized with octahedral symmetry such that there are eight hydrophilic channels (3-fold symmetric) and six hydrophobic channels (4-fold symmetric) that traverse the protein shall and can allow the passage of ions and molecules. The subunits are also classified as either H- or L-chain,^733^ where the ratio of H- to L-chains is greater in ferritins located in tissues such as the heart that require fast iron metabolism, and L-chains are more abundant in ferritins in tissues that benefit from long-term iron storage, like the liver.^743,748^ Ferritins that are rich in L-chains form more crystalline cores than their H-chain-rich counterparts.^748^ A recent study has also shown that the presence of a mineral core reduces the stability of the protein cage,^749^ which indicates that the protein structure is altered by the mineral core.

The mechanism by which the mineral core forms and dissolves has received considerable attention. Soluble ferrous iron enters the cage through the 3-fold channels,^750,751^ while the 4-fold hydrophobic channels are thought to be involved in the proton and/or oxygen transfer that are required to maintain the correct pH and redox potential within the lumen.^750,751^ The ferritin cage is therefore able to concentrate iron ions, which increases supersaturation, and together with control over the pH and phosphate, this promotes the formation of ferrihydrite. Apoferritin also possesses ferroxidase and nucleation sites that act in concert to drive mineral formation. The ferroxidase center is found only on the H-chain, where it is embedded at the center of the subunit 4-helix bundle.^734,738,752^ It is responsible for the oxidation of Fe­(II) to Fe­(III) during hydrolysis,^732,753,754^ as described by eq 3:^748,755^

3 $${\left[\mathrm{Fe}{\left(\mathrm{II}\right)}_{2\left(\mathrm{FC}\right)}\mathrm{-P}\right]}^{z+4}+O_{2}+4H_{2}O\to 2{\left[\mathrm{Fe}\left(\mathrm{III}\right){\mathrm{OOH}}_{\left(\mathrm{core}\right)}\mathrm{-P}\right]}^{z}+H_{2}O_{2}+4H^{+}$$

*P* indicates protein-bound iron, the subscripts *FC* and *core* indicate binding to the ferroxidase center and mineral core, respectively, and *z* is the net charge on the protein. The protons generated in the process are shuttled away to maintain the higher pH that favors iron storage over dissolution, and the ferroxidase center is regenerated to its apo-form by transfer of the oxidized species to a separate nucleation center.^748^ NMR has shown that there is a 20 Å channel within the subunit that guides the Fe­(III) ions toward the internal cavity and promotes the formation of multimers.^756^ 8 Fe­(III) ions can occupy this channel, and the close proximity of multiple exit channels in the ferritin cavity is proposed to promote nucleation. Once the core reaches a certain size (of around 800 metal atoms), the mineral surface becomes the dominant mechanism for oxidation and autocatalytically oxidizes Fe­(II) to Fe­(III).^733,748,757^

Following the oxidation of Fe­(II) and transfer of Fe­(III) species to the inner cavity, apoferritin accelerates the nucleation of ferrihydrite. The nucleation site has long been associated with the L-chain, and early work using site-directed mutagenesis and chemical modification suggested that this is attributed to the acidic glutamate residues Glu57, Glu60, Glu61 and Glu64.^30,758^ A recent study in which high resolution single crystal XRD was used to analyze the structure of human L-chain homopolymer recombinant ferritin soaked in ferrous iron solutions supported this analysis. This showed that glutamic acid residues (Glu60, Glu61 and Glu64) were involved in binding iron to the internal surface of the ferritin lumen, and identified a tri-iron cluster at the putative nucleation site.^743,759^ Further glutamate residues (Glu57 and Glu140) assist in building this cluster.^743,759^ More recently, a cryo-TEM single particle reconstruction study of human L-chain ferritin was able to resolve the protein mineral interface of partially mineralized structures.^900^ The 2.85 Å (PDB 9BPJ) very nicely resolved the side chains interacting with the mineral, and showed differences in the side chain positions between this mineralized structure and the tri-iron octa-iron bound X-ray structures (PDB 5LG8 and 6TS1). This shows that these residues change conformation upon binding and accumulating iron precursors, and then change again as these transform to the ferrihydrite mineral phase. This demonstrates that the protein-mineral interface is dynamic when facilitating biomineral nucleation and growth.

Additional information about the nucleation mechanism has been obtained from high-resolution TEM studies. High angle annular dark field images from individual L-chain rich ferritin cores located in tissue sections of human liver were recorded using spherical aberration corrected STEM under controlled electron doses.^760^ Three-dimensional reconstruction of the core morphologies showed that each core comprises up to eight subunits, with no crystallographic relationship between them. This is consistent with nucleation occurring close to the eight 3-fold channels, as suggested by NMR studies,^756^ or at 8 symmetric nucleation positions on the interior of the lumen. A recent single-particle cryo-TEM study of the structure of bacterioferritin at different stages of mineralization also revealed the formation of small particles near the termini of the 3-fold channels, and data suggested that these subsequently merge to give a single core.^761^ As such, mineral nucleation in ferritins may occur in different places in different ferritins, either associated with iron binding motifs on the interior lumen, or adjacent to pores.

Extensive work has employed the ferritin cage as a nanoreactor in which to deposit nonferrihydrite minerals, often for applications in nanotechnology.^751,762–773^ We here highlight some of the studies that have given insight into the mechanisms by which native ferritin generates ferrihydrite cores. Deposition of MnOOH^774–776^ and Fe_3_O_4_ ^762^ provide examples where mineralization is accompanied by a change in oxidation state of the metal.^769,777^ Incubation of apoferritin with Mn­(II) at pH 8.9 results in the formation of amorphous Mn­(III) oxyhydroxide cores within the protein, together with some nonspecific precipitation of crystalline Mn_3_O_4_.^774–776^ Notably, the size of the mineral cores is independent of the metal loading (between ≈1000–3000 Mn ions/ferritin molecule), which contrasts with the systematically smaller cores seen at lower loadings in the Fe system.^776^ A similar behavior is seen when recombinant human L-chain ferritin (which does not possess ferroxidase sites) is reconstituted with Fe.^737,749,759^ This further emphasizes the role of the ferroxidase site, where its absence results in an “all-or-nothing” behavior in which the ferritin molecules are either empty or filled to maximum capacity. Mineral nucleation is slow due to the reduced activity of the protein, but once formed, the cores readily grow, filling the protein cage. This demonstrates the importance of nucleation vs growth in the activity of ferritin.

Magnetite cores have also been generated within ferritin under reconstitution conditions of pH 8.5 and 60 °C, where Fe­(II) was added to the protein in increments with slow oxidation.^762^ These conditions also generate magnetite in the absence of the protein. That magnetite preferentially formed within the protein again suggests that the ferroxidase site functions to oxidize Fe­(II) under these conditions, promoting magnetite core formation.

An investigation into the reconstitution of ferritin with alkaline earth metal carbonates (CaCO_3_, BaCO_3_, SrCO_3_) and calcium phosphate further emphasized the importance of the combined action of the ferroxidase and nucleation sites in promoting ferrihydrite formation.^770^ Simple incubation of apoferritin in a cation solution (Ca^2+^, Sr^2+^ or Ba^2+^) and exposure to aqueous Na_2_CO_3_ or CO_2_ resulted in precipitation in the bulk solution rather than within the protein.^770^ However, addition of the crystallization inhibitors poly­(methacrylic acid) (PMMA) or polyphosphate to the reaction solution caused the mineral to form uniquely within the protein shell.^770^ These agents are too large to enter the protein cavity, such that crystallization is only possible within the protein, similar to the mechanism proposed for HAp mineralization within collagen gaps (Sections 3.5 and 7.2). Importantly, amino acid residues present on the interior of the ferritin cage must actively chelate and concentrate the metal ions, driving the formation of crystal nuclei within the lumen.

### 7.4 Magnetosome Nanoreactors

As discussed in Section 4.2, magnetotactic bacteria contain proteo-lipid organelles that are mineralized with magnetic nanoparticles with precise control. Again, we focus on magnetite forming species and explore how the structure and confinement of the magnetosome controls mineralization within these nanoreactors.

Like ferritin, magnetotactic bacteria are also excellent model systems for understanding biomineralization processes, where the genetic code of a number of strains has been sequenced.^778–780^ This has identified about 30 key genes that generate proteins associated with magnetosome production.^781,782^ The confinement of the magnetosome allows magnetotactic bacteria to precisely control the formation of the magnetite crystals. This is achieved by (i) creating the chemical conditions required to precipitate magnetite, where this is achieved by pumping ions into the lumen to create a supersaturated solution, and pumping protons to control the pH and redox potential, (ii) ensuring that a single nucleation event occurs, and (iii) controlling the growth of the magnetic particles to give crystals with defined sizes and shapes.^397^

Studies involving the mutation and knockout of magnetosome genes, as well as in vitro work, have vastly increased our understanding of the roles that individual proteins play in magnetite formation. As discussed in Section 4.2, recombinant or extracted versions of these proteins are not able to fully replicate magnetosome particle sizes and shapes ex vivo in bulk solution. One major factor is the absence of the confinement of the magnetosome membrane, which highlights the importance of organization and confinement in mineralizing magnetosomes. The magnetosome vesicle is constructed prior to mineralization by invagination from the inner cell membrane,^405,406,783,784^ and magnetosome membrane proteins are sorted to the magnetosome either before, during, or after this process. Iron is then transported into the vesicle, resulting in the formation of magnetite (Figure 9).^397,406,422,785^ Here, we focus on the magnetosome of *Magnetospirillum* strains MSR-1 and AMB-1, which generate cuboctahedral particles, and outline the roles of individual components in building the magnetosome nanoreactor and controlling magnetite formation.

The principal proteins active in this process are shown in Figure 35, the majority of which are coassembled with the magnetosome organelle to perform specific functions and create the nanoreactor that directs the formation of magnetite crystals. Notably, the complement of proteins may vary depending on the stage of maturation.^781,786^ In MSR-1 mutants in which the ≈30 magnetosome genes have been knocked out, proper magnetosome formation can be restored with just seven *mam* genes,^784^ with MamB (a putative iron transporter)^787^ being the most crucial protein for proper magnetosome formation.^784,788,789^ The full list of protein names, functions, and references are in Table 1, with some major proteins and their functions in the magnetosome discussed below.

**Figure 35:**
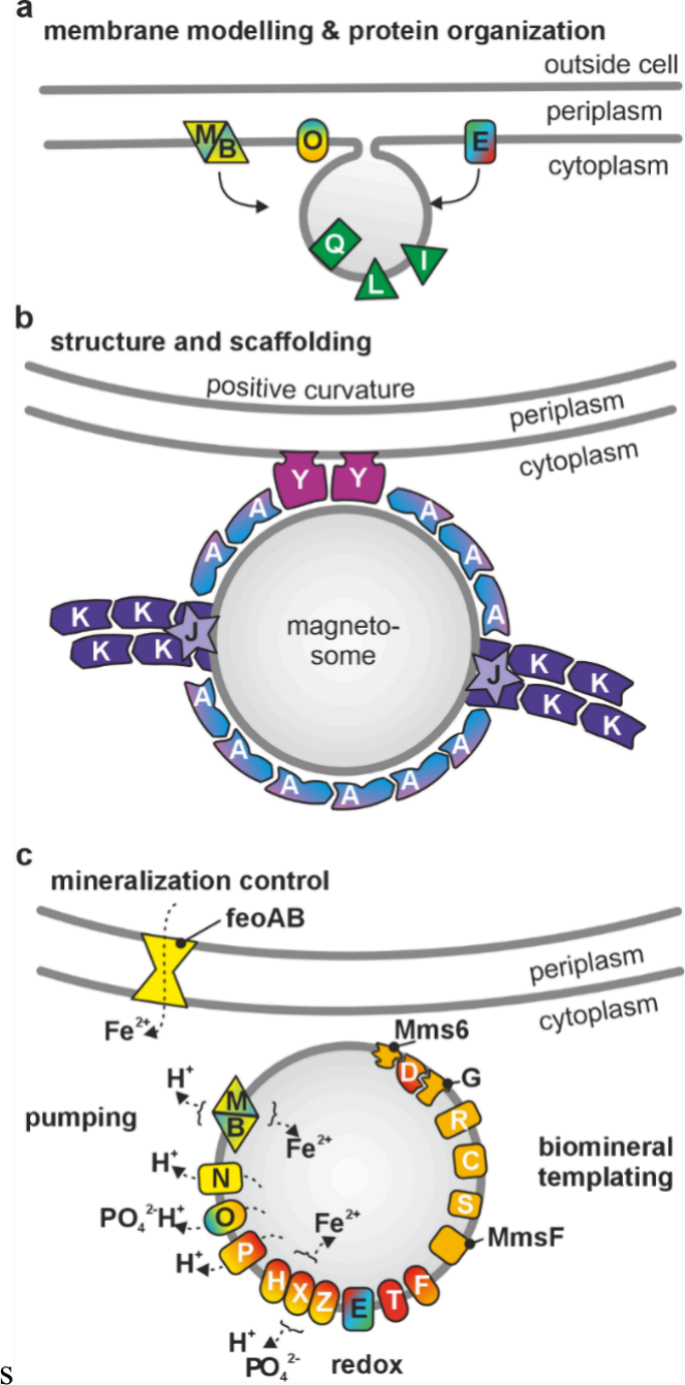
**Diagram showing positions and functions of proteins involved in magnetosome biogenesis**, based on analysis in Uebe and Schuler 2016 ^397^ and Barber-Zucker and Zarivach^781^ and references therein. (a) Proteins involved in membrane invagination and modeling. (b) Structural proteins. (c) Proteins involved in mineralization control (pumps, redox, nucleation and mineral templating). Proteins are colored to indicate their role in magnetosome biomineralization in *M. magneticum* AMB-1 and *M. gryphiswaldense* MSR-1 and are all named “Mam: unless indicated otherwise. Some proteins are thought to have more than one role, so they appear in more than one section and have more than one color. Green, membrane bending and/or modification; blue, protein recruitment and processing; purple, structural scaffolding and positioning; yellow, pumping to accumulate reactants (Fe^2+^), adjust pH (H^+^), and/or remove inhibitors (PO_4_^3–^); red, redox control of oxidation of Fe^2+^ to Fe^3+^; and orange, biotemplating (e.g., crystal nucleation, size or shape templating).

**Table 1:** List of Proteins Involved in Magnetosome Formation, Their Function, and References^a^

Protein name	Function
MamA	Self-assembles into a curved structure^790,791^ that coats the external surface of the developing magnetosome.^251^ Provides structure to empty magnetosome nanoreactor, and acts as a docking locus for the organization of other magnetosome biomineralization proteins.^251,791–793^
MamE	Critical role: magnetic particles are not formed if it is fully deleted, and the crystal size is reduced if the catalytic center of MamE is deleted.^786,794^ Involved in particle formation and maturation, probably recruiting proteins for the former, and the catalytic center participates in the latter.^794^
MamB, M	MamB and MamM co-assemble to form a cation diffusion facilitator protein to transport Fe^2+^ into the magnetosome lumen and pump protons out.^787^
MamN	Proton efflux.^787,788,793,795,796^
MamP, H, X, Z	Iron transport and redox control.^788,795,797,798^
MamP, X, T, E	Contain heme-binding domains called magnetochromes that are involved in iron oxidation.^787,797,799^
MamP	Oxidizes ferrous iron to form ferrihydrite as a precursor to magnetite.^798^
MamI	Help iron oxide precursors transform into magnetite.^397,788,800^
MamE, M, O	Controlling magnetite nucleation^397^
MamS	Ensures single nucleation event occurs in each magnetosome.^785,788,795^
MamR	Control crystal size and number (not clear how),^786,788,795^ *Δmamr* mutants produce shorter chains of magnetosomes containing smaller particles.^787,801^ A putative DNA binding domain^795^ indicates that this protein may facilitate the assembly or regulation of other biomineralization proteins on the magnetosome.
MamC (Mms13), D (7), G (5); MmsF; Mms6	Small partial membrane proteins that are found in the *Alphaproteobacteria* strains appear to have crystal size and shape templating roles.^414,781,802^
MamC, D, G (Mms13, 7, 5); Mms6	Specify cuboctahedral morphology by binding to specific crystal faces of magnetite.^347,429,802–808^
MamD, G (Mms5, 7); Mms6	Thought to self-assemble and anchor to the magnetosome membrane,^787,795,803,808^ where they can interact with facets of the developing mineral.

a Some proteins may appear more than once as some have more than one function. If proteins have more than one name, the Mms version is in brackets.

Magnetotactic bacteria must accumulate iron, and a high iron concentration and basic pH are necessary for the formation of the magnetite crystals.^789^ Cation diffusion facilitator proteins transport iron in and pump protons out.^787^ This is necessary as magnetite precipitation produces more protons than it consumes,^809^ as per eq 4:

4 $$2{\mathrm{Fe}}^{3+}+{\mathrm{Fe}}^{2+}+4H_{2}O\to {\mathrm{Fe}}_{3}O_{4}+8H^{+}$$

Prior to and during magnetite formation, the redox of the magnetosome must also be carefully controlled in order to generate the mixed valence iron oxide magnetite. The aqueous chemistry of iron is complex,^744^ so precise redox and pH control are required. The nucleation of magnetite is also highly controlled within the magnetosome. Possible precursors to magnetite have been detected, and these include crystalline, amorphous, oxidized, or reduced phases.^418,810–814^ Single nucleation events have been observed on the magnetosome inner surface in magnetospirilla^401,434,436^ and MV-1 species,^422^ and a number of proteins have been implicated in controlling this nucleation process. The size and number of crystals formed in each cell is also carefully controlled, with magnetosome proteins implicated in controlling this part of the process also identified (see Table 1). Experiments with mutants in which specific proteins have been eliminated have confirmed the importance of particular proteins in determining the size and shape of the magnetosome crystals.^414,433,803,815^

This shows that magnetosomes employ a large number of proteins to ensure the formation of magnetite. Many of these proteins only generate magnetite when they are organized in the magnetosome and so are not able to regulate mineralization in isolation in vitro. Some control the transport of iron, protons and electrons to create the right conditions for magnetite formation within the confines of the magnetosome lumen, but there is not one protein alone that is able to do the job of the magnetosome. There have been some successful experiments where the ability to make magnetite containing magnetosomes has been transferred to a number of non-magnetotactic strains.^416,816^ Many magnetotactic bacterial strains are difficult to cultivate as they have slow growth rates, and require carefully controlled media composition, redox and pH. As such, it is highly desirable to identify new species that can be easily cultivated, which would make it easier to scale up magnetosome production for medical or industrial applications, or to which other desirable functions (e.g., photosynthesis^416^) could be added.

Liposomes and polymersomes are self-assembled structures that have been used as mimics of magnetosome reactors. Polymersomes of poly­(ethylene oxide)-polybutadiene were used to encapsulate a basic NaOH solution and were then electroporated in the presence of Fe^2+^ and Fe^3+^ to introduce iron ions into the vesicles.^817^ Small paramagnetic nanoparticles precipitated adjacent to, or within the polymersome membrane,^817^ but did not grow to fill the entire volume. Constructing polymersomes with a negatively charged interior resulted in the formation of electron dense magnetic particles that formed in the polymersome lumen rather than within the membrane.^818^ When a similar process was used with liposomes prepared from 1,2-distearoyl-*sn*-glycero-3-phosphocholine and higher concentrations of the iron salts and base, a magnetic iron oxide particle was formed in each vesicle.^819^ This demonstrates that the chemistry of the lumen within magnetosomes plays a key role in the formation of magnetite particles.

We are not aware of any work in which protein pumps have been used to create artificial magnetosomes, where it is challenging to ensure that the pumps are oriented correctly (protons out, iron in). However, a demonstration that fusion proteins can be used to control the orientation of light-driven proton pumps^820^ suggests that it should be possible to create a proteo-liposome that mimics a magnetosome. This would make it possible to increase the pH within a liposome encapsulating iron, and to correctly orient an iron symporter, thereby creating an artificial magnetosome that could be used in biotechnological applications^821–824^ and to test the function of magnetosome proteins. Variation in the size of the liposomes would also make it possible to systematically study the effects of confinement on the mineralization process.

## 8 Summary and Perspective

The organization and function of biological systems is built around compartmentalization. However, the role that confinement plays in biomineralization processes is still poorly understood. This review has provided a discussion of the ways in which organisms use confinement to control mineralization, where these range from the well-accepted, such as the role of compartmentalization in concentrating and transporting precursors to the site of mineralization, and isolating the mineralization site within the organism, to more speculative ideas including the effects of confinement on crystal orientation and polymorph.

### 8.1 Confinement as a Means of Controlling Crystallization

Our current knowledge of the effects of confinement on crystallization principally comes from studies of abiotic systems, where interest derives from their importance to many real-world crystallization processes.^65^ Crystallization within porous media is fundamental to geochemical phenomena such as frost heave and the formation of ore deposits,^825,826^ and can cause massive damage to structural materials.^530^ The freezing of water in nanopores is a key element of ice nucleation in the atmosphere,^827,828^ while templating by hard and soft environments is often used to create nanomaterials.^122^ Confined systems including isolated droplets and arrays of droplets have also been exploited to study crystallization processes,^82,829,830^ where they offer impurity-free environments and vast arrays of droplets can be exploited to determine nucleation kinetics. That nucleation is retarded in small volumes has also allowed confined systems to be used to study the polymorphism of molecular organic crystals and pharmaceuticals.^66,67^ Finally, a wide range of well-defined systems that offer confinement over length scales ranging from the micrometer to the nanoscale have been exploited to investigate the effects of confinement on the crystallization of a wide range of organic and inorganic compounds.^66,67^

Together, these studies have created a valuable framework for predicting and understanding the effects of confinement on crystal nucleation and growth. Significant effects are observed at nucleation. Nucleation rates can be vastly reduced in small volumes due to the lower probability of nucleation and the elimination of impurities and competing nuclei.^831–833^ A nucleus forming within a small, finite volume will also consume ions/molecules as they grow, which continuously reduces the supersaturation and therefore the driving force for nucleation.^85^ The influence of confinement on morphology is well-understood, where single crystals and polycrystalline materials grown within rigid templates are typically molded by the template.^122,123,524^ The existence of a thin film of solution between the crystal and the template wall ensures that the crystal can grow to fill the entire mold, resulting in single crystals with complex noncrystallographic morphologies and curved surfaces that could not be generated using soluble additives in bulk solution.^527^ There are also many examples of confinement effects leading to control over crystal orientation, where competitive growth of crystals with anisotropic structures within anisotropic environments can lead to preferred orientations,^127,834^ as can preferential nucleation of a crystal face at the pore wall.^118,132^

Confinement can also have dramatic effects on the crystal structure and polymorphs. Crystallization within environments such as carbon nanotubes that have dimensions comparable to those of critical nuclei (generally a few nanometers) typically produces crystals with distorted lattices, structures not seen under analogous bulk conditions, or even entirely new structures.^100,102^ Many organic compounds form as amorphous or metastable crystalline phases within small pores, where polymorphs with critical nuclei larger than the pore diameter are prevented from forming.^109,835^ Confinement effects are also seen at length-scales that vastly exceed the size of a critical nucleus,^99,114^ where metastable polymorphs are frequently formed and can exhibit lifetimes that are orders of magnitude greater than in bulk solution.^93,96^ The kinetic origins of these effects arise from the creation of very high supersaturations in small volumes and reduced diffusion rates within small pores.^117^ Surfaces also become of increasing importance in small volumes and may play a role in determining which polymorph forms first.^98^ The long lifetime of metastable polymorphs can in turn be ascribed to the reduced probability of a more stable polymorph forming within the same small volume and destabilizing the metastable phase.^118^

### 8.2 Compartmentalization in Biomineralization

These basic principles also apply to biomineralization processes. However, organisms have a toolbox that allows them to exploit confinement to a far greater degree than is possible in synthetic/abiotic systems. (1) The impurities that are impossible to eliminate in synthetic systems are completely excluded in biological systems, enabling organisms to create ultraclean environments in which they are uniquely able to control crystallization. (2) The presence of ion pumps and passive channels in membranes allow organisms to precisely tune the composition of the crystallization solution over time. As such, a continuous supply of precursor materials can be provided to a crystal until it grows to fill an entire isolated volume. (3) Precursor species can be introduced into the mineralization compartment at specific locations, directing crystal growth toward these ion sources. (4) Proteins/macromolecules can be positioned at specific sites in the mineralization compartment, defining the number and position of nucleation sites or binding to growing crystal faces to control crystal morphologies. (5) Crystallization in synthetic systems invariably takes place in the presence of counterions, often at significant concentrations. The types and concentrations of counterions can again be controlled in biological systems. (6) Organisms can continue to evolve both the mineralization compartment and the growing mineral simultaneously, restructuring the environment as the mineral grows.

Confinement therefore provides organisms with a unique opportunity to gain exquisite control over biomineralization processes to create highly oriented biominerals with complex morphologies, gaining spatial control over their compositions, potentially stabilizing metastable polymorphs and triggering their transformation or dissolution as desired.

### 8.3 Challenges and Opportunities: Confinement in Biomineralization

A huge amount of work remains to be done to elucidate the ways in which organisms exploit confinement to control mineralization processes. Ideally, we would wish to perform time-resolved studies and follow the development of a system from nucleation—where characteristics such as location, polymorph, and orientation are defined—to the product biomineral. We would also wish to study both the mineral and the associated organic framework, and the relationship between the two. Imaging-based techniques are required to study the development of individual mineral components, and entire systems should be studied over multiple length-scales in their native, hydrated state. Looking historically, simple optical microscopy provided sufficient resolution to watch the growth of sea urchin larval spicules from micrometer-sized rhombohedral crystals to triradiate structures within organic compartments,^496,503^ but few systems can be studied in this way, and the resolution of optical microscopy is limited.

An example of an accessible nanoscale biomineralization system is provided by the 2D bacterial *S*-layer of *Geobacillus stearothermophilus* DSM 13240, which was studied with TEM and AFM.^836^ This study identified 3–5 nm pores in which a hydrated calcium carbonate nucleated, before transforming into different calcium carbonate polymorphs via a number of solid-state and dissolution-reprecipitation processes.^836^ Localizing mineralization on this surface is thought to help the organism survive in environments containing toxic, high concentrations of calcium, conferring an evolutionary advantage, and is an example of forced biomineralization.^837^

Developments in cryogenic sample preparation for SEM and TEM have revolutionized the study of biomineralizing organisms, where these can preserve both the mineral and organic components, and enable the relationship between these to be visualized.They also eliminate the need to embed samples in resin, which is often associated with artefacts. Cryo-FIB-SEM allows 3D images of frozen samples to be created by imaging sequential cuts through a sample and offers resolutions of ≈10 nm.^838^ Cryo-TEM offers superior resolution (≈1 Å) and cryo-ET can be used to reconstruct 3D images from TEM data.^839^ However, cryo-TEM sample thickness is limited to ≈300 nm, such that only a limited volume of the organism can be studied at one time. The cryo-FIB sectioning process is also challenging as it is difficult to vitrify samples with large volumes prior to sectioning, and the organic tissues are usually sensitive to ion-beam irradiation, so can easily damage.^840^ Alternatively, cryo-ultramicrotomy can be used to thin-section the samples, but the differences in hardness between the organic tissues and the mineral can create artifacts during cutting.^841^

The need to characterize samples over multiple length scales is particularly challenging. For example, organic matrices (e.g., lipid bilayers) are structured on the nanoscale while mature biominerals can reach macroscopic dimensions, and many biominerals are structured over multiple length-scales (e.g., bone^553,573^ ). While spectroscopic methods such as in situ NMR and XRD can provide valuable information about the structural evolution of biominerals,^718,842^ electron and X-ray microscopy are as yet the only imaging techniques that can simultaneously resolve both organic (e.g., lipid bilayer vesicle membrane of ≈5 nm) and inorganic features (e.g., amorphous or crystalline mineral) at the nanoscale.^481,843,844^ The latest research in advanced imaging techniques in biomineralization was highlighted by a recent Faraday Discussion.^845^ The most informative studies have therefore been conducted on small, comparatively simple mineralizing organisms. These include algae and bacteria, as these contain small mineral components that can be imaged in association with a confining organic matrix,^70,405^ and they are not buried in layers of material as is the case for larger life-forms.

Developments in correlative imaging-based techniques are thus extremely exciting, where these promise the ability to image and analyze both the organic and inorganic components and to explore the association between the two.^69,151^ These include correlative light and electron microscopy (CLEM),^846–848^ which combines fluorescence microscopy with high-resolution electron microscopy and enables the location of specific, fluorescently labeled proteins with known or putative functions (pumping, templating, etc.) to be identified, and their location with respect to the mineral or organic structures be determined. Proteins can also be selectively labeled with heavy metals to map their locations using EELS in combination with STEM,^849^ and it is possible that high resolution techniques such as single particle cryo-TEM can be adapted to image the organic/mineral interface as has been demonstrated for ferritin.^900,761^ Going beyond imaging-based methods, advances in manipulating the genetics of organisms, like the precision offered by CRISPR-Cas9 editing tools,^850^ have revolutionized our ability to explore biological function by targeting the genetics of an organism.^838^ Genetic manipulation could be used to investigate how changes in processes related to confinement, such as the construction of confined environments, localization of biotemplating molecules, and the transport of precursor species, influence biomineralization.

### 8.4 Challenges and Opportunities: Applying Biogenic Confinement Strategies to Synthetic Systems

There are also many opportunities to take inspiration from biomineralization processes to use confinement to control mineralization in synthetic systems. As described in this review, there is strong evidence that basic biogenic design strategies—such as defining the geometry of the environment in which crystals grow—can be adapted to control mineralization in synthetic systems. There are many examples where confined systems such as vesicles, membrane pores and thin organic films (e.g., SAMs, Langmuir monolayers, or polysaccharide layers) have been used very effectively to control features including morphology, polymorph, single crystal/polycrystallinity, and orientation. These are typically very simple by design, and there is undoubtedly scope to construct environments that can potentially control multiple features at one time. However, these systems can often not be scaled-up, and few can deliver materials to rival native biominerals.

There is therefore significant interest in exploiting biomineralizing organisms to synthesize minerals. A simple example is provided by ferritin (Section 7.3), which is designed by nature to synthesize and store the iron oxyhydroxide ferrihydrite. Ferritins have been used to biotemplate a range of non-native semiconductor, metallic, magnetic and catalytic materials,^769,851–853^ where this is facilitated by the excellent thermal and chemical stability of the protein cage and its intrinsic design. The metal-binding sites present on the internal surface of the protein cage and the ion-shuttling pores ensure that nucleation is favored within the protein, enabling a wide range of nanomaterials to be formed within the confines of the lumen. Inspired by the activity of ferritin, other protein cages that do not naturally mineralize, such as virus capsids^854–856^ and the E2 core protein from the pyruvate dehydrogenase multienzyme complex of *Geobacillus stearothermophilus*,^857^ have been explored as biomineral nanoreactors, where mineralization can be promoted by techniques including genetic modification^858,859^ or the introduction of peptides into the cage.^857^

Recent advances in de novo protein design and assembly^777,860–863^ also promise the ability to design and construct protein cages that can promote the formation of nanoparticles of target minerals with specific sizes, shapes and crystallinity. This vastly increases the scope of using protein nanocages to synthesize nanoparticles, where it becomes possible to generate very specific sizes and shapes that cannot be created using cages extracted from biological systems. Our knowledge of the biomineralization mechanisms of ferritin, and how this protein acts catalytically in the oxidation of Fe^2+^ to Fe^3+^ and in promoting nucleation is clearly invaluable in designing such proteins. While initial steps have been made in designing proteins capable of templating mineral formation,^864–866^ there is a vast scope to further develop this field.

There is also enormous potential in using organisms to work for us, as evidenced by the growth of areas including synthetic biology and engineered living materials.^867–870^ So-called “bionic materials” with enhanced properties have been created by adding nanoparticles to the growth medium of organisms.^871,872^ Although principally explored for the enhancement of the mechanical properties of spider silk,^871^ there is potential to use this strategy to enhance the properties of biominerals, where initial experiments have been conducted to attempt to incorporate magnetic nanoparticles in the calcite skeletons of foraminifera, creating novel composite materials.^872^ This is an interesting approach, where surface-functionalization of the nanoparticles could lead to superior uptake.^869,873^

Bacteria and other organisms such as fungi are also being widely explored to synthesize nanoparticles and have been used to synthesize a vast range of materials.^867,874–879^ Metal-tolerant bacteria have been shown to biomineralize gold and other precious metals^880,881^ when incubated in media containing metal ions,^882^ or after extraction from mining waste soils.^883–885^ While this process is often extracellular,^886^ it can also occur within the organism.^887^ A recent study showed that *Escherichia coli* formed fluorescent semiconductor nanoparticles within the confines of the periplasm when supplemented with metal ions (Cd^2+^) and a sulfur source (cysteine).^888^ The periplasm of *E. coli* contains biomolecules that could facilitate this process, for example, one of the confining membranes supports electron transport machinery for the cell.^888^ This creates a 2D confined environment (20–50 nm high) into which *E. coli* deposits the CdS semiconductor nanoparticles. When the confinement was removed by cell lysis, the metastable mineral clusters recrystallized to form larger, more crystalline nanoparticles.^888^

In many of these cases, mineral formation occurs as a byproduct of a detoxification process. The organism removes toxic ions from solution by precipitating minerals, often by adapting existing compartments, machinery and nucleating surfaces to help mineral formation and storage. For example, bacterial cells under the appropriate metal stressor commonly precipitate minerals within the periplasmic space between the inner and outer membrane, which has been shown for platinum and palladium,^889^ gold,^890^ silver,^891^ copper sulfide,^892^ cadmium sulfide,^888^ lead phosphate,^893^ and iron phosphate.^894^ If this confers an evolutionary advantage to the organism, then these abilities are passed on or even enhanced. For example, the evolution of ferritin is thought to have occurred in response to increased atmospheric oxygen levels about 2 billion years ago.^895^ This enabled organisms to detoxify ferric iron from their cytoplasm, allowing the safe storage of iron as ferrihydrite until the iron is needed, e.g. for enhancing enzyme active sites.

Powered by developments in molecular biology, there are also exciting opportunities to use genetic engineering approaches to enhance the ability of organisms to create minerals.^896^ Considering first organisms that have not evolved to produce minerals, recombinant *E. coli* coexpressing metallothionein and phytochelatin synthase proteins have been used to synthesize a wide range of nanoparticles with controlled size, shape and batch-to-batch reproducibility.^897^ The yeast *Pichia pastoris* was genetically engineered to overexpress the cytochrome b5 reductase enzyme and efficiently produced silver and selenium nanoparticles.^898^ Sequencing the genetic code of biomineralizing organisms also promises the ability to directly modify the minerals they produce. Examples are still limited to simple organisms and have been best demonstrated for magnetotactic bacteria. For example, controlled expression of the mms7 gene in *M. magneticum* strain AMB-1 resulted in a change in the morphology of the magnetite crystal shape from dumbbell-like to spherical^815^ and overexpression of magnetosome biosynthesis genes in *M. gryphiswaldense* MSR-1 led to a high yield of larger crystals.^417^

It has also proven possible to endow nonbiomineralizing bacteria with the ability to generate magnetite nanocrystals by identifying the key genes needed for magnetosome biosynthesis in *M. gryphiswaldense*, and transferring them to other bacterial hosts.^416,816^ The resulting new magnetosome-producing strains are easier to culture, such that they can potentially be used to synthesize magnetite nanocrystals. Interesting work is also being conducted with diatoms, whose genomes have been sequenced.^80,899^ Genetic manipulation of these organisms is already providing new understanding of the mechanisms by which mineralization occurs, and it is just a matter of time before we are able to use these approaches to control features such as mineral morphologies.

### 8.5 Concluding Remarks

Throughout this review, we have focused on processes that have evolved in organisms to direct the formation of biominerals and have demonstrated the vital role that confinement plays in enabling and controlling these processes. These vary from fundamental roles including the sequestration and delivery of precursor materials, isolation of the mineralization site, and the creation of anisotropic growth environments to direct effects on mineralization. Confinement often plays a key role in defining mineral shape, where this is particularly important in creating complex morphologies that could not be generated using soluble additives alone. It also allows exceptional control over the number and location of crystals within an environment, where the latter has an impact on competitive growth, and thus the creation of more complex structures and the development of orientation. As yet, effects on polymorph remain speculative, although there is clear evidence that confinement can stabilize metastable phases, giving organisms the chance to control or prevent their transformation to more stable phases if required. Continued progress in understanding the strategies that nature uses to control biomineralization – including the critical role played by confinement – will ultimately aid in the development of treatments for illnesses related to mineral formation such as abnormalities in the development of bones and teeth, and in the prevention of pathological mineralization conditions including atherosclerosis, kidney stones and gout. It can also inspire the development of environmentally friendly methods for synthesizing minerals with characteristics to rival those of biological systems, which can deliver new generations of materials and contribute to a more sustainable future.

## Abbreviations

ACC: amorphous calcium carbonate
ACP: amorphous calcium phosphate
AFM: atomic force microscopy
AG: anhydrous guanine
CAP: coccolith-associated polysaccharide
CLEM: correlative light and electron microscopy
cryo-ET: cryogenic electron tomography
cryo-SEM: cryogenic scanning electron microscopy
cryo-TEM: cryogenic transmission electron microscopy
CV: coccolith vesicle
DNA: deoxyribonucleic acid
ECM: extra-cellular matrix
EDX: energy dispersive X-ray spectroscopy
EELS: electron energy loss spectroscopy
EV: extracellular vesicle
FCC: face-centered cubic
FFT: fast Fourier transformation
FIB: focused ion beam
HAp: hydroxyapatite
LCPA: long-chain polyamine
MTB: magnetotactic bacteria
NMR: nuclear magnetic resonance
pAA: poly­(acrylic acid)
pAH: poly­(allylamine hydrochloride)
PAR: poly­(adenosine diphosphate-ribose)
pAsp: poly­(aspartic acid)
PIC: polarization-dependent image contrast
PILP: polymer induced liquid precursor
PLL: poly-l-lysine
PMC: primary mesenchymal cell
PVA: poly­(vinyl alcohol)
ROY: 5-methyl-2-[(2-nitrophenyl)­amino]-3-thiophenecabonitrile
rVEGF: recombinant vascular endothelial growth factor
SAED: selected area electron diffraction
SAM: self-assembled monolayer
SDV: silica deposition vesicle
SEM: scanning electron microscopy
STEM: scanning transmission electron microscopy
STV: silicon transport vesicle
TE: track-etched
TEM: transmission electron microscopy
TP: Tomes’ process
TPMS: triply periodic minimal surfaces
XANES: X-ray absorption near-edge structure spectroscopy
XFM: X-ray fluorescence microscopy
X-PEEM: X-ray photoemission electron spectromicroscopy
XRD: X-ray diffraction
